# Monocyte-derived macrophages orchestrate multiple cell-type interactions to repair necrotic liver lesions in disease models

**DOI:** 10.1172/JCI166954

**Published:** 2023-08-01

**Authors:** Dechun Feng, Xiaogang Xiang, Yukun Guan, Adrien Guillot, Hongkun Lu, Chingwen Chang, Yong He, Hua Wang, Hongna Pan, Cynthia Ju, Sean P. Colgan, Frank Tacke, Xin Wei Wang, George Kunos, Bin Gao

**Affiliations:** 1 Laboratory of Liver Diseases, National Institute on Alcohol Abuse and Alcoholism (NIAAA), NIH, Bethesda, Maryland, USA.; 2 Department of Hepatology and Gastroenterology, Charité Universitätsmedizin Berlin, Berlin, Germany.; 3 Laboratory of Human Carcinogenesis and; 4 Liver Cancer Program, Center for Cancer Research, National Cancer Institute, NIH, Bethesda, Maryland, USA.; 5 Department of Anesthesiology, Critical Care and Pain Medicine, McGovern Medical School, University of Texas Health Science Center at Houston, Houston, Texas, USA.; 6 Department of Medicine, School of Medicine, University of Colorado Anschutz Medical Campus, Aurora, Colorado, USA.; 7 Laboratory of Physiologic Studies, NIAAA, NIH, Bethesda, Maryland, USA.

**Keywords:** Gastroenterology, Hepatology, Hepatitis, Macrophages

## Abstract

The liver can fully regenerate after partial resection, and its underlying mechanisms have been extensively studied. The liver can also rapidly regenerate after injury, with most studies focusing on hepatocyte proliferation; however, how hepatic necrotic lesions during acute or chronic liver diseases are eliminated and repaired remains obscure. Here, we demonstrate that monocyte-derived macrophages (MoMFs) were rapidly recruited to and encapsulated necrotic areas during immune-mediated liver injury and that this feature was essential in repairing necrotic lesions. At the early stage of injury, infiltrating MoMFs activated the Jagged1/notch homolog protein 2 (JAG1/NOTCH2) axis to induce cell death–resistant SRY-box transcription factor 9^+^ (SOX9^+^) hepatocytes near the necrotic lesions, which acted as a barrier from further injury. Subsequently, necrotic environment (hypoxia and dead cells) induced a cluster of complement 1q–positive (*C1q*^+^) MoMFs that promoted necrotic removal and liver repair, while *Pdgfb*^+^ MoMFs activated hepatic stellate cells (HSCs) to express α–smooth muscle actin and induce a strong contraction signal (YAP, pMLC) to squeeze and finally eliminate the necrotic lesions. In conclusion, MoMFs play a key role in repairing the necrotic lesions, not only by removing necrotic tissues, but also by inducing cell death–resistant hepatocytes to form a perinecrotic capsule and by activating α-smooth muscle actin–expressing HSCs to facilitate necrotic lesion resolution.

## Graphical abstract

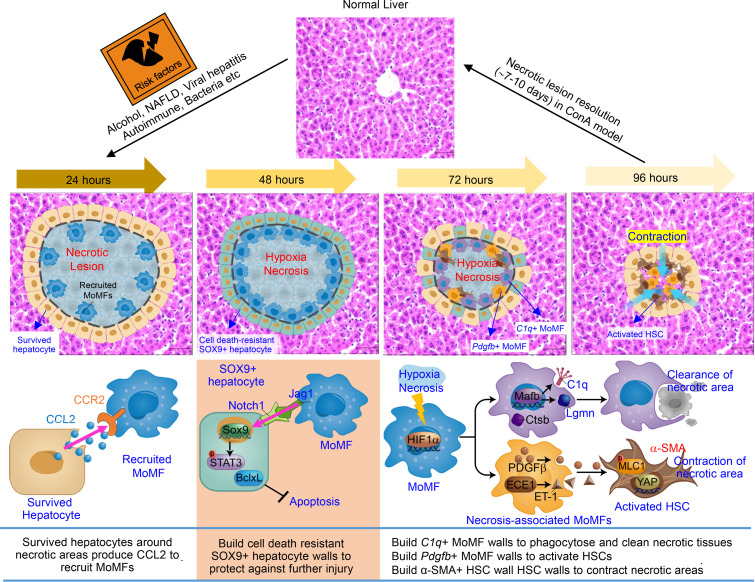

## Introduction

The liver has a strong capacity for regenerating after tissue loss or injury. Two-thirds partial hepatectomy (PHx) in rodents has been extensively used as a model for studying the mechanisms of liver regeneration (1). Studies using the PHx model have identified a large number of growth factors and cytokines and their downstream signals that promote hepatocyte proliferation (1–3). Liver regeneration has also been studied in a wide variety of acute liver-injury models that are associated with liver necrotic lesions (2–5), which are not observed in the PHx model. In addition, liver necrotic lesions are frequently observed in human liver diseases, such as viral hepatitis, alcohol-associated liver disease, nonalcoholic fatty liver disease, autoimmune liver disease, and drug-induced liver injury (4, 5). Although necrotic lesions of the liver had been characterized almost 8 decades ago (6), how they are eliminated and repaired remains largely unknown. For example, how are the hepatocytes adjacent to the necrotic lesions protected from further injury? How are the dead cells and debris removed from the necrotic lesions? How are the necrotic lesions eliminated? Do hepatocytes or liver progenitor cells (LPCs) migrate inside the necrotic lesions to repair the lesions?

Inflammation plays important roles not only in contributing to liver injury, but also in promoting repair and fibrosis resolution (3). Thus, in the current study, we used a well-established immune-mediated acute liver-injury model induced by concanavalin A (ConA) (7) to study the resolution of necrotic lesions and their repair by inflammatory cells. Injection of ConA, a T cell mitogen, rapidly activates T cells and subsequently activates other types of immune cells (NKT cells, neutrophils, Kupffer cells) that release a variety of proinflammatory cytokines (TNF-α, IFN-γ, IL-4, IL-5, etc.), causing liver necrosis that manifests in massive necrotic lesions (8, 9). After injection of a nonlethal dose of ConA, substantial liver necrosis occurs, which is rapidly resolved within a few days. Massive hepatocyte proliferation is observed and likely plays an important role in repairing the liver in the ConA model (10). However, how the necrotic lesions are resolved in this model remains unknown. To begin to answer this question, we performed multiplex immunofluorescence staining using a variety of immune cell markers and nonparenchymal cell markers, which revealed that monocyte-derived macrophages (MoMFs) are the major cell type that encircles the necrotic area throughout the resolution of liver necrosis. The role of MoMFs in repairing liver necrotic lesions was further investigated by establishing subsets of MoMFs based on their unique gene expression profiles, as revealed by single-cell sequencing.

The normal liver contains a large number of resident macrophages called Kupffer cells and a much smaller number of infiltrating MoMFs. Significant intrahepatic infiltration of MoMFs occurs in almost all liver-injury models via the interaction between C-C motif chemokine ligand 2 (CCL2) and its receptor, CCR2 (11). Both Kupffer cells and infiltrating MoMFs have been characterized not only as proinflammatory cells, but also as having a key role in tissue repair, depending on the stage of injury and recovery process (11–13). For example, depletion of liver macrophages before injection of acetaminophen (APAP) or inhibition of MoMF recruitment early after APAP reduced APAP-induced liver injury, whereas depletion of macrophages after APAP injection accelerated liver injury (14–16). Similarly, depletion of liver macrophages before ConA injection diminished ConA-induced liver injury (17), while their depletion after ConA injection exacerbated liver injury, as demonstrated in the current study. Blockage of MoMF recruitment also markedly exacerbated ConA-induced liver injury (18, 19). Whereas most previous studies have focused on the immunoregulatory and phagocytic functions of infiltrated MoMFs in liver injury and repair, here we demonstrate that in ConA-induced immune-mediated liver injury, MoMFs play a key role, not only in phagocytosing necrotic tissues, but also in orchestrating the induction of SRY-box transcription factor 9^+^ (SOX9^+^) hepatocytes and the activation of hepatic stellate cells (HSCs) to promote the resolution and repair of necrotic lesions. We have identified 2 clusters of these MoMFs by single-cell RNA (scRNA) sequencing, one expressing complement 1q (*C1q*) and the other expressing *Pdgfb*. Further analyses revealed that *C1q*^+^ MoMFs express several genes related to phagocytosis and protein digestion and thus play an important role in removing necrotic tissues, while *Pdgfb*^+^ MoMFs promote the activation and contraction of HSCs that surround the necrotic areas and contribute to the elimination of necrotic lesions.

## Results

### MoMFs are aggregated and encapsulate the necrotic lesions following immune-mediated liver injury.

**Figure 1:**
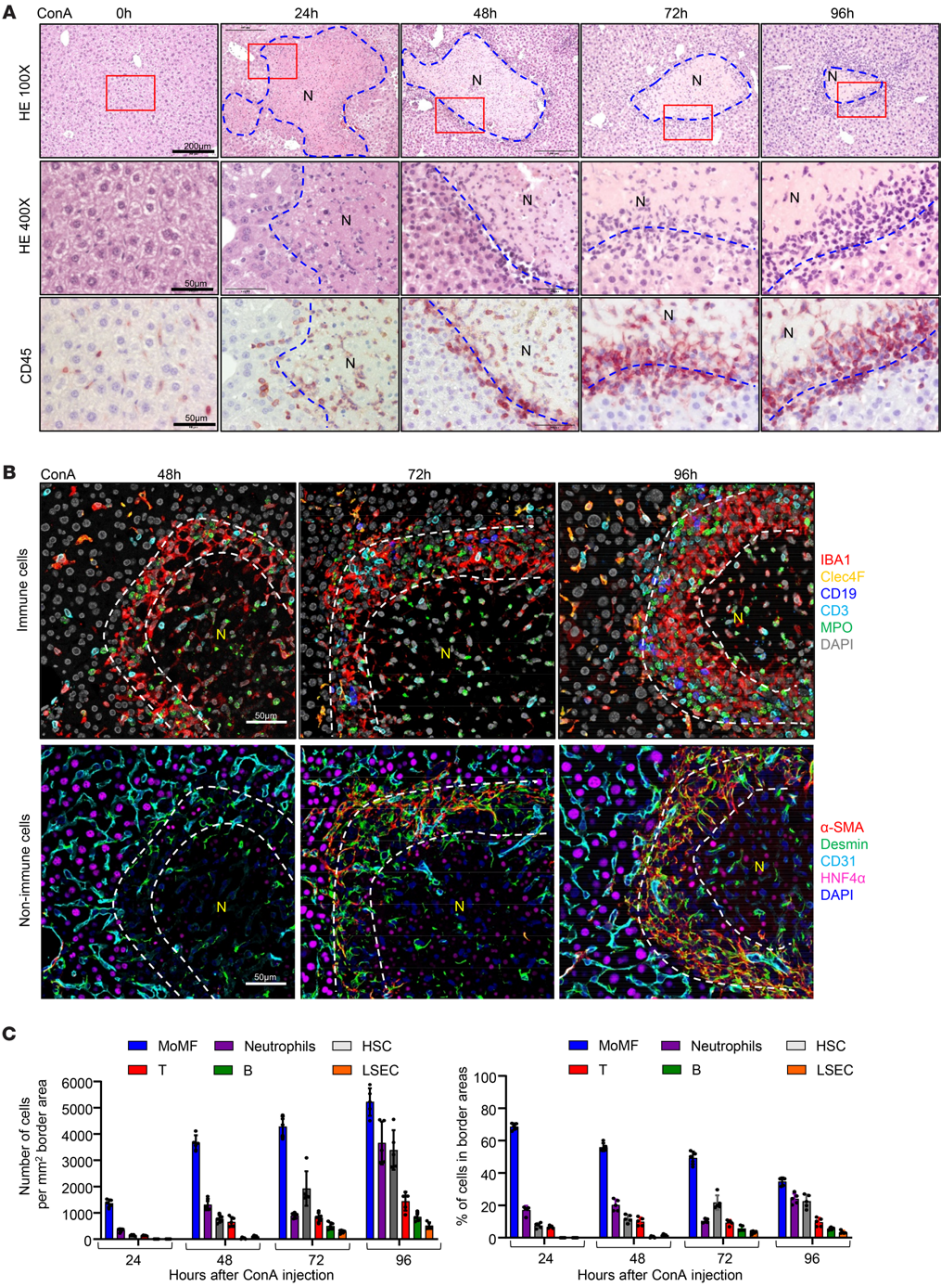
MoMFs and aHSCs are the major cell types encapsulating necrotic lesions after ConA-induced liver injury. C57BL/6 mice were treated with 12 mg/kg ConA. Liver samples were collected 24, 48, 72, and 96 hours after ConA treatment. (**A**) Liver sections were stained with CD45 antibody and H&E. Representative images are shown (*n* = 5). (**B** and **C**) Multiplex immunofluorescent staining of several cell markers was performed on liver sections with necrotic lesions. Representative images are shown in **B** (*n* = 5). Quantification of number for each cell type identified in the border areas (indicated by dash lines) of necrotic regions is shown in **C**. The number and percentage of each type of cells are represented as means ± SD (*n* = 5). N, necrotic area.

To study the mechanisms by which necrotic lesions are resolved after acute liver injury, we utilized the ConA-induced immune-mediated acute liver-injury model, in which massive liver tissue damage, including hepatocyte necrosis, develops within 24 hours after ConA injection, resulting in rapid elevation of blood transaminase (ALT) (Supplemental Figure 1A; supplemental material available online with this article; https://doi.org/10.1172/JCI166954DS1) and large necrotic lesions (Figure 1A). These lesions then are gradually reduced to smaller sizes by 96 hours (Figure 1A) and eventually disappear within 7 days. During the development of injury and its gradual resolution, we also noticed aggregation of nonparenchymal or inflammatory cells surrounding the necrotic areas, which separated undamaged hepatocytes from the necrotic areas, as demonstrated by H&E staining (Figure 1A). Immunostaining analyses revealed that many of these aggregated cells were CD45^+^ immune cells, forming a “ring-like” structure that had become thicker during the resolution of the necrotic lesion (Figure 1A).

Further studies using multiplex immunofluorescence staining with different cell markers revealed 3 major cell types surrounding the necrotic lesions. As illustrated in Figure 1, B and C, and Supplemental Figure 1B, MoMFs (IBA1^+^CLEC4F^–^: IBA1, a general macrophage marker; CLEC4F, a Kupffer cell marker) were detected as early as 24 hours after ConA injection and aggregated abundantly at later time points. Transplantation of GFP bone marrow experiments (Supplemental Figure 1C) revealed a large number of bone marrow–derived GFP^+^ cells deposited on the border area, further supporting that most IBA1^+^ cells surrounding the necrotic areas are bone marrow–derived MoMFs. Activated HSCs (aHSCs) (Desmin^+^α-SMA^+^) and neutrophils (MPO^+^) were mainly detected at late time points (approximately 72–96 hours after ConA injection) (Figure 1, B and C). IBA1^+^CLEC4F^+^ double-positive Kupffer cells were mainly detected in nondamaged parenchymal regions, but few Kupffer cells were detected inside necrotic areas and their border regions (Figure 1B and Supplemental Figure 1B), suggesting that the Kupffer cells originally located in the necrotic areas are probably destroyed and new Kupffer cells do not migrate to the necrotic areas, which is in agreement with a previous study demonstrating that Kupffer cells do not migrate (20). The number of Kupffer cells in nondamaged areas was stable during ConA-induced liver injury (Supplemental Figure 1D). In addition, we also observed the proliferation of Ki67^+^IBA1^+^CLEC4F^+^ Kupffer cells in nondamaged areas (Supplemental Figure 1E). Finally, although they do not migrate after injury, Kupffer cells (IBA1^+^Clec4F^+^) have been shown to play an important role in initiating ConA-induced liver injury by producing a variety of proinflammatory cytokines (17, 21). To further define the functions of Kupffer cells, we isolated Kupffer cells from ConA-treated liver, and we performed reverse-transcription–quantitative PCR (RT-qPCR) analysis and found that several of the markers related to activation and phagocytosis of Kupffer cells were upregulated 6 hours after ConA injection, but remained unchanged during the later repair stage 48 to 72 hours after ConA injection (Supplemental Figure 1F).

To define the mechanisms underlying the recruitment of MoMFs, we performed several experiments using the ConA model in *Ccr2*^–/–^, *Cx3cr1*^–/–^, and *Ccl2^Hep–/–^* mice (Supplemental Figure 2, A–D). Our data revealed that at the early stages of injury, hepatocytes that surround necrotic lesions produce the MoMF chemokine CCL2, which induced MoMF accumulation via CCR2 and not the C-X3-C motif chemokine receptor 1 (CX3CR1) (see detailed results in Supplemental Figure 2, A–D). Interestingly, deletion of *Ccr2* markedly reduced the number of MoMFs without affecting the number of Kupffer cells (Supplemental Figure 2C). In addition, we detected high expression levels of activated NF-κB (nuclear p65 staining), which is known to upregulate CCL2 (22) and which may contribute to CCL2 production, in the hepatocytes surrounding the necrotic border area (Supplemental Figure 2E).

### MoMFs trigger the formation of a cell death–resistant SOX9^+^ hepatocyte layer encapsulating the necrotic areas via the JAG1/NOTCH2 pathway at early time points.

**Figure 2:**
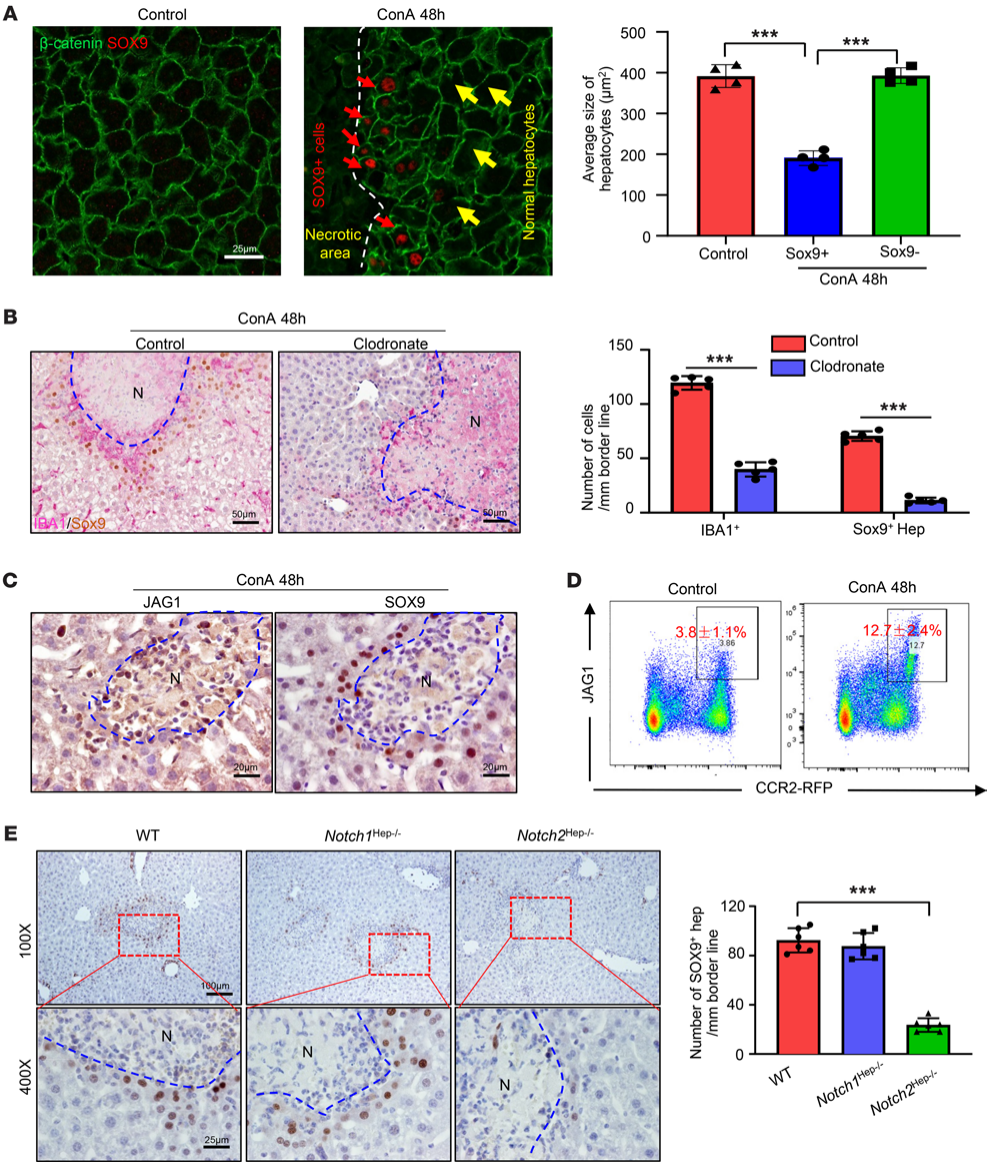
MoMF-derived JAG1 signaling is required for the generation of SOX9^+^ hepatocytes in the early stages of liver repair after injury. (**A**) Representative immunofluorescent staining of SOX9 and β-catenin of liver tissues from ConA-treated mice (48 hours after treatment, *n* = 4). Red arrows indicate SOX9^+^ hepatocytes in the ConA-injured liver; yellow arrows indicate normal hepatocytes. β-Catenin shows membrane of hepatocytes. The average sizes of hepatocytes were quantified based on β-catenin staining. (**B**) Mice were treated with ConA for 24 hours, followed by injecting clodronate liposomes or control liposomes. Liver samples were collected 48 hours after ConA treatment. SOX9 and IBA1 double staining were performed on these samples (*n* = 5). (**C**) Mice were treated with ConA for 48 hours, followed by staining of liver tissues with JAG1 and SOX9 antibodies (*n* = 5). (**D**) Heterozygous CCR2-RFP mice (CCR2^+^ MoMFs are labeled with RFP) were treated with ConA. Liver MNCs were isolated and subjected to flow cytometry analyses of JAG1 and RFP (*n* = 5). (**E**) WT and hepatocyte-specific *Notch1-* and *Notch2*-knockout mice were treated with ConA for 48 hours. SOX9 protein in the liver tissues was stained, and the number of SOX9^+^ hepatocytes was quantified (*n* = 6). Representative images are shown in **A**, **B**, **C**, and **E**. Values in **A**, **B**, **D**, and **E** are represented as means ± SD. Statistical significance was assessed using 2-tailed Student’s *t* test for comparing 2 groups (**B**) and 1-way ANOVA followed by Tukey’s post hoc test for multiple groups (**A** and **E**). ***P < 0.001. Dashed lines indicate the borderlines of necrotic areas.

To define whether LPCs/stem cells contribute to necrotic lesion repair, we performed immunostaining with a SOX9 antibody. SOX9 is a marker for LPCs and biliary epithelial cells, but is not expressed in mature hepatocytes (23), playing a critical role in controlling specification of hepatoblasts to bile duct cells (BDCs) via the activation of notch homolog protein (NOTCH) (24, 25). Our immunostaining experiments only detected SOX9-positive staining in BDCs in normal liver; however, in the livers from ConA-treated mice, BDCs and small hepatocytes surrounding necrotic areas stained positively with SOX9, with peak effects occurring 48 and 72 hours after ConA injection (Figure 2A and Supplemental Figure 3A). By using several hepatocyte and LPC/BDC markers, we demonstrated that these SOX9^+^ cells resemble hepatocytes rather than progenitor/BDCs; thus, hereinafter we refer to these cells as SOX9^+^ hepatocytes (see detailed results in Supplemental Figure 3, B–F).

Interestingly, a large number of IBA1^+^CLEC4F^–^ MoMFs and SOX9^+^ hepatocytes were coaggregated surrounding the necrotic areas (Supplemental Figure 3B). To determine whether MoMFs contribute to induction SOX9^+^ hepatocytes, we performed macrophage depletion by using clodronate-containing liposomes 8 hours after ConA injection. Indeed, macrophage depletion remarkably reduced the number of SOX9^+^ cells (Figure 2B). Clodronate-containing liposomes depleted all IBA1^+^ cells, including both Kupffer cells and MoMFs (Figure 2B) as well as circulating monocytes, without reducing neutrophils (Supplemental Figure 4A). To further define the role of MoMFs in inducing SOX9^+^ hepatocytes, we used *Ccr2^–/–^* mice, which are deficient in MoMF recruitment receptor CCR2 and had markedly reduced MoMF aggregation in the necrotic borders (Supplemental Figure 2). As illustrated in Supplemental Figure 4B, the number of SOX9^+^ hepatocytes was reduced by 90% in *Ccr2*^–/–^ mice compared with that in WT mice. In contrast, *Cx3cr1*^–/–^ mice, which had similar MoMF aggregation on necrotic borders (Supplemental Figure 2), had numbers of SOX9^+^ hepatocytes similar to those of control mice after ConA injection (Supplemental Figure 4B).

To explore the possible signaling pathways that control SOX9 expression, we purified CD45^+^CD11b^+^CCR2^+^Ly6G^–^ MoMFs and quantified the mRNA levels of several factors involved in regulating SOX9 expression. Among them, Jagged1 (JAG1) expression was significantly upregulated after ConA treatment (Supplemental Figure 5A). JAG1, a cell-surface protein that interacts with neurogenic locus NOTCH receptors, has been implicated in the lineage specification of LPCs/BDCs by promoting SOX9 expression via the activation of NOTCH in a cell-cell contact manner (26). The mRNA expression of NOTCH target genes, such as *Hes1* and *Hes5* as well as *Sox9*, in the liver was also significantly upregulated by ConA treatment (Supplemental Figure 5B). Immunohistochemical staining visualized strong JAG1 expression on immune cells around the necrotic area and adjacent to the SOX9^+^ hepatocytes in ConA-treated mice, while very weak JAG1 staining was detected in normal livers or in nondamaged areas (including Kupffer cells) of the livers from ConA-treated mice (Figure 2C), suggesting that JAG1 is highly upregulated in MoMFs but not in Kupffer cells after ConA injection. Furthermore, in CCR2 GFP heterozygous mice, JAG1 expression was localized to CCR2^+^ MoMFs and was significantly increased after ConA injection (Figure 2D). Furthermore, flow cytometric analyses revealed that JAG1 was mainly localized in MoMFs, but not in T cells, B cells, NK cells, or neutrophils before or after ConA injection (Supplemental Figure 6). Interestingly, the number of JAG1^+^ MoMFs was elevated in the liver but not in the spleen 24 to 72 hours after ConA injection, and these cells expressed various macrophage/monocyte markers (Supplemental Figure 6).

Next, we explored the role of JAG1/NOTCH signaling in inducing SOX9^+^ hepatocytes using mice with hepatocyte-specific deletion of *Notchs*, the genes encoding receptors for JAG1 (*Notch1^Hep–/–^* and *Notch2^Hep–/–^*). As illustrated in Figure 2E, the number of SOX9^+^ hepatocytes was markedly reduced in *Notch2^Hep–/–^* but not in *Notch1^Hep–/–^* mice compared with control mice. Reduced numbers of SOX9^+^ hepatocytes were also observed in mice treated with the NOTCH signaling inhibitor dibenzazepine (DBZ) or following the ablation of JAG1^+^ MoMF by intermedilysin (ILY) treatment (Supplemental Figure 7, A and B). These data suggest that MoMF-derived JAG1 promotes SOX9^+^ hepatocyte generation via the activation of NOTCH.

### SOX9^+^ hepatocytes are derived from mature hepatocytes and limit liver injury by activating survival signaling without contributing to hepatocyte proliferation.

We next performed cell-fate tracing experiments to determine the origin of the SOX9^+^ hepatocytes by using Sox9 Cre^ERT^Rosa26-EYFP reporter mice, in which approximately 70% of CK19^+^ BDCs/LPCs and a few periportal cells were labeled with EYFP (Supplemental Figure 8, A and B). We found that all SOX9^+^ cells were EYFP negative in Sox9 Cre^ERT^Rosa26-EYFP reporter mice after ConA injection (Supplemental Figure 8C). We also used AAV-TBG-Cre^+^Rosa-EYFP reporter mice, in which more than 96% hepatocytes but no other cells in the liver were labeled with EYFP (Supplemental Figure 9, A and B) (27, 28). In this case, all SOX9^+^ hepatocytes were EYFP positive in AAV*-*TBG-Cre^+^Rosa-EYFP reporter mice after ConA injection (Supplemental Figure 9C). Collectively, our data suggest that SOX9^+^ hepatocytes are derived from mature hepatocytes, but not from LPCs/BDCs.

**Figure 3:**
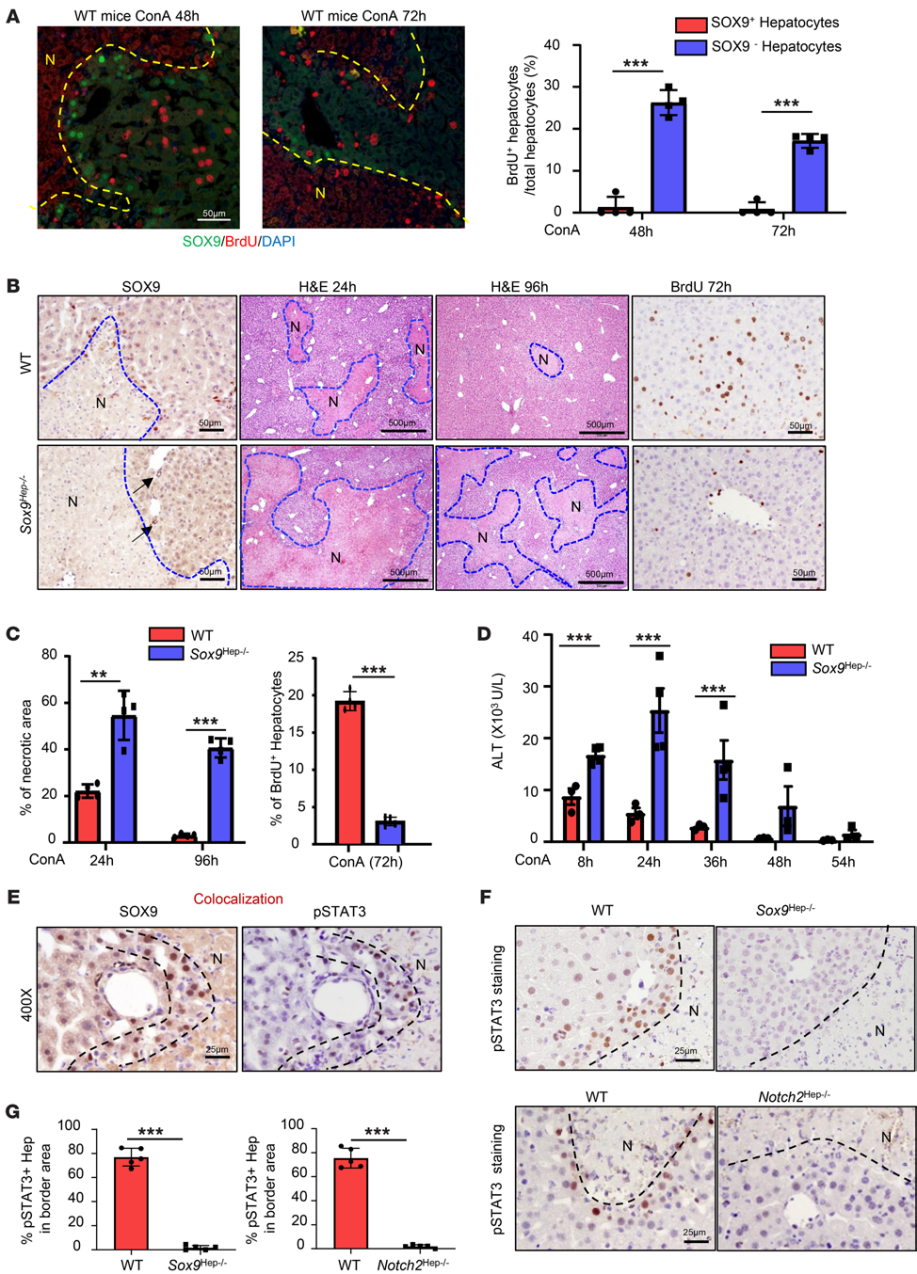
Evidence for the resistance of SOX9^+^ hepatocytes to cell death. (**A**) C57BL/6 mice were treated with ConA for 48 and 72 hours; BrdU was given 2 hours before sacrifice. BrdU and SOX9 double staining of liver tissues (see representative images on the left) (*n* = 4). Percentages of BrdU^+^ hepatocytes were quantified (right). (**B**) WT and *Sox9^Hep–/–^* mice were treated with ConA for different times; BrdU was given 2 hours before sacrifice. Representative SOX9, H&E, and BrdU staining of liver tissues is shown (*n* = 4–5). Arrows indicate SOX9^+^ BDCs. (**C**) Percentages of necrotic area and percentages of Brdu^+^ hepatocytes from **B** were quantified. (**D**) Serum ALT levels were analyzed (*n* = 3–4). (**E**) C57BL/6 mice were treated with ConA for 48 hours. SOX9 and pSTAT3 staining of serial sections of liver tissues. Representative images are shown (*n* = 5). (**F**) WT, *Sox9^Hep–/–^*, and *Notch2^Hep–/–^* mice were treated with ConA for 48 hours. pSTAT3 was stained for the liver tissues (*n* = 5). (**G**) Percentages of pSTAT3^+^ hepatocytes from **F** were quantified (*n* = 5). Dashed lines indicate the borderlines or border areas of necrotic regions. Values in **A**, **C**, **D**, and **G** are represented as means ± SD. Statistical significance was assessed using 2-tailed Student’s *t* test for comparing 2 groups (**A**, **C**, **D** and **G**). **P < 0.01; ***P < 0.001.

Previous studies reported that SOX9^+^ hepatocytes located in the periportal area had greater regenerative potential compared with hepatocytes elsewhere in the liver (29, 30). To our surprise, in the ConA model, most proliferating hepatocytes were detected in nondamaged parenchymal regions where hepatocytes did not express SOX9, while no proliferation of SOX9^+^ hepatocytes was detected as demonstrated by BrdU staining (Figure 3A) and Ki67 staining (Supplemental Figure 10), suggesting that SOX9^–^ but not SOX9^+^ hepatocytes proliferate to replace the dead hepatocytes during ConA-induced injury. In addition, neither hepatocytes nor LPCs were detected in the necrotic areas during liver lesion resolution, as shown in Figure 1B, suggesting that proliferating SOX9^–^ hepatocytes do not penetrate the SOX9^+^ cell wall to replace the necrosis areas and that proliferating SOX9^–^ hepatocytes likely expand the parenchymal regions to squeeze the necrotic lesions.

To further define the role of SOX9 in hepatocytes in immune-mediated liver injury, we deleted *Sox9* in hepatocytes only by injecting AAV8-TBG-Cre into *Sox9^fl/fl^* mice (*Sox9^Hep–/–^*). SOX9^+^ hepatocytes were observed around the necrotic lesions in WT mice, but not in *Sox9^Hep–/–^* mice (Figure 3B). H&E staining and BrdU incorporation assay demonstrated that *Sox9^Hep–/–^* mice had much larger necrotic areas and impaired Brdu^+^ hepatocyte proliferation than WT mice after ConA injection (Figure 3, B and C). Consistent with the larger necrotic areas, *Sox9^Hep–/–^* mice had higher levels of serum ALT (Figure 3D) and a higher mortality rate compared with WT mice after ConA injection (Supplemental Figure 11A). It was interesting to note that WT mice had peak ALT levels 8 hours after ConA injection, while *Sox9^Hep–/–^* mice had much higher levels and prolonged elevation of ALT (Figure 3D), suggesting that SOX9^+^ hepatocytes play an important role in preventing the liver from further damage after ConA injection.

To understand the underlying mechanisms by which SOX9 promotes cell survival, we checked the antiapoptotic proteins, such as STAT3 and BCL-xL. We found that most SOX9^+^ cells encapsulating necrotic areas were stained positively with pSTAT3 (Figure 3E) and deletion of *Sox9* or *Notch2* in hepatocytes abolished pSTAT3 staining (Figure 3, F and G). Similarly, strong BCL-xL staining was detected and overlapped with the SOX9-stained areas, and deletion of *Sox9* or *Notch2* in hepatocytes reduced BCL-xL staining (Supplemental Figure 11, B and C). In addition, treatment with the Notch signaling inhibitor DBZ in ConA-treated mice reduced hepatocyte proliferation, enlarged the necrotic area, exacerbated serum ALT elevation, and reduced pSTAT3 and BCL-xL expression (Supplemental Figure 12, A–C). ILY-mediated ablation of JAG1^+^ cells, which reduced SOX9 expression (Supplemental Figure 7B), also markedly reduced pSTAT3 and BCL-xL expression in small hepatocytes around necrotic areas (Supplemental Figure 12C).

### aHSCs encapsulate the necrotic areas with strong cell contraction signal activation during the resolution phase.

**Figure 4:**
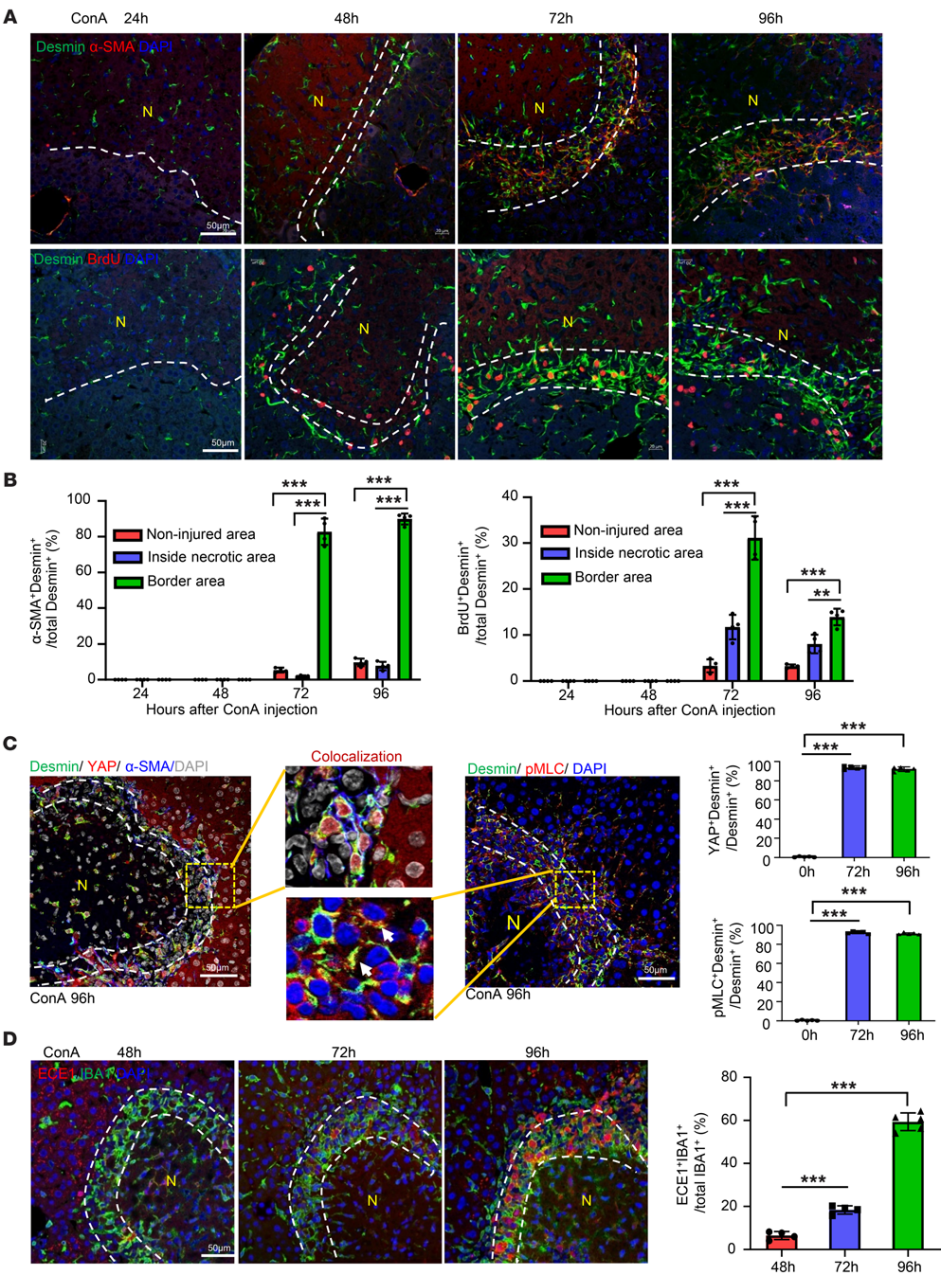
aHSCs aggregate in the border areas of necrosis at the late-stage recovery of live injury. (**A** and **B**) C57BL/6 mice were treated with ConA. BrdU was given 2 hours before sacrifice. Liver tissues were collected and stained with desmin/α-SMA and desmin/BrdU. Representative images are shown in **A** (*n* = 4). Quantification of aHSCs and proliferating HSCs in noninjured area, inside necrotic area, and border area in **A** is shown in **B**. (**C**) Liver tissues from ConA-treated mice were stained with desmin/YAP/α-SMA or desmin/PMLC. Representative triple- or double-staining images are shown (*n* = 5). Quantitation of the percentages of YAP^+^Desmin^+^ and pMLC^+^Desmin^+^ in border areas was performed. (**D**) Liver tissues from ConA-treated mice were stained with IBA1/ECE1 (*n* = 4–5). Dashed lines indicate the border areas of necrotic regions. Quantitation of ECE1^+^IBA1^+^/total IBA1^+^ cells in border areas was performed. Values in **B**–**D** are represented as means ± SD. Statistical significance was assessed using 1-way ANOVA followed by Tukey’s post hoc test for multiple groups (**B**–**D**). **P < 0.01; ***P < 0.001.

MoMFs and HSCs are the 2 major cell populations making up the ring-like structure during the resolution phase (approximately 72 to 96 hours after ConA injection; Figure 1, B and C), which was also characterized by strong α-SMA staining and increased BrdU incorporation in desmin^+^ HSCs (Figure 4, A and B), suggesting that the accumulation of MoMF may be coupled with HSC proliferation and activation. Interestingly, aHSCs also expressed high levels of nuclear YAP and phosphorylated myosin light chain (pMLC) (Figure 4C), 2 key signals for smooth muscle cell contraction (31, 32). To better understand the mechanisms underlying HSC contraction, we examined the expression of the endothelin-converting enzyme-1 (ECE1), a membrane-bound metalloprotease (33) that proteolytically converts big endothelin-1 (ET-1) into active ET-1, a key factor for inducing HSC contraction by activating pMLC (32). As illustrated in Figure 4D, strong ECE1 protein expression was detected in the necrotic border areas overlaying IBA^+^ MoMFs, especially at the later time points (96 hours). ET-1 staining was detectable in liver sinusoid cells in naive mice; however, in liver from ConA-treated mice, ET-1 staining was extended to include necrotic areas (Supplemental Figure 13).

### MoMFs play an important role in inducing the formation of an aHSC layer and promote necrotic area resolution.

**Figure 5:**
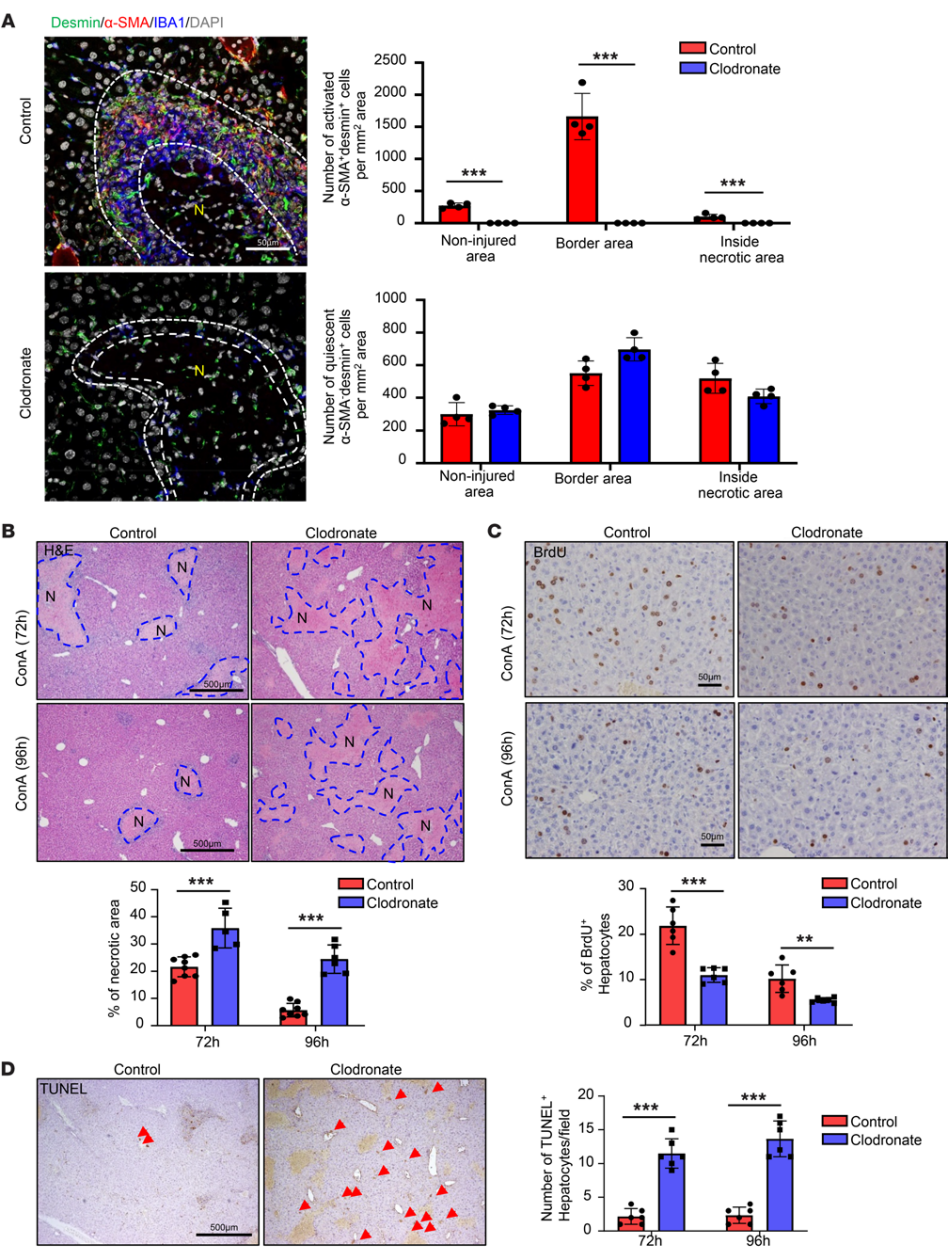
Depletion of MoMFs after injury abolishes HSC aggregation and exacerbates ConA-induced liver injury. C57BL/6 mice were treated with ConA. Clodronate liposome or control liposome was given to these mice 24, 48, and 72 hours after ConA injections. Mice were euthanized 96 hours after ConA injection. BrdU was given 2 hours before sacrifice. Liver tissues were stained with IBA desmin and α-SMA (**A**, *n* = 4), H&E (**B**, *n* = 5-8), BrdU (**C**, *n* = 6), and TUNEL (**D**, *n* = 6). Arrows indicate TUNEL^+^ hepatocytes. Representative images are shown. Dashed lines indicate the border areas of necrotic regions. Values are represented as means ± SD. Statistical significance was assessed using 2-tailed Student’s *t* test for comparing 2 groups (**A**–**D**). **P < 0.01; ***P < 0.001.

To determine whether the expansion and activation of HSCs were dependent on MoMFs, we depleted MoMFs with clodronate-containing liposomes 48 hours after ConA injection and performed immunostaining. As illustrated in Figure 5A and Supplemental Figure 14A, depletion of IBA1^+^ MoMFs by clodronate was associated with a near-complete loss of activated α-SMA^+^desmin^+^ HSCs without affecting quiescent HSCs (α-SMA^–^desmin^+^) (Figure 5A). Furthermore, the resolution of liver damage and initiation of liver regeneration were significantly delayed by clodronate treatment, as evidenced by larger necrotic areas, reduced hepatocyte proliferation (BrdU^+^), but increased hepatocyte death (TUNEL^+^) compared with control liposome treatment (Figure 5, B–D). Because clodronate-containing liposomes depleted both Kupffer cells and MoMFs (as shown in Figure 2B), we used *Ccr2^–/–^* mice that had markedly reduced MoMFs without affecting Kupffer cells (as shown in Supplemental Figure 2C) to further define the role of MoMFs in inducing the activation of αSMA^+^ HSCs. Our data revealed that deletion of *Ccr2* markedly reduced the number of α-SMA^+^ HSCs while deletion of the *Cx3cr1* did not affect them (Supplemental Figure 14B). Taken together, all these data suggest that the ring-like structure composed of MoMFs and HSCs is essential for efficient liver repair after acute injury.

### Identification of 2 clusters of necrosis-associated MoMF populations by scRNA-Seq, including C1q^+^ MoMFs and Pdgfb^+^ MoMFs.

**Figure 6:**
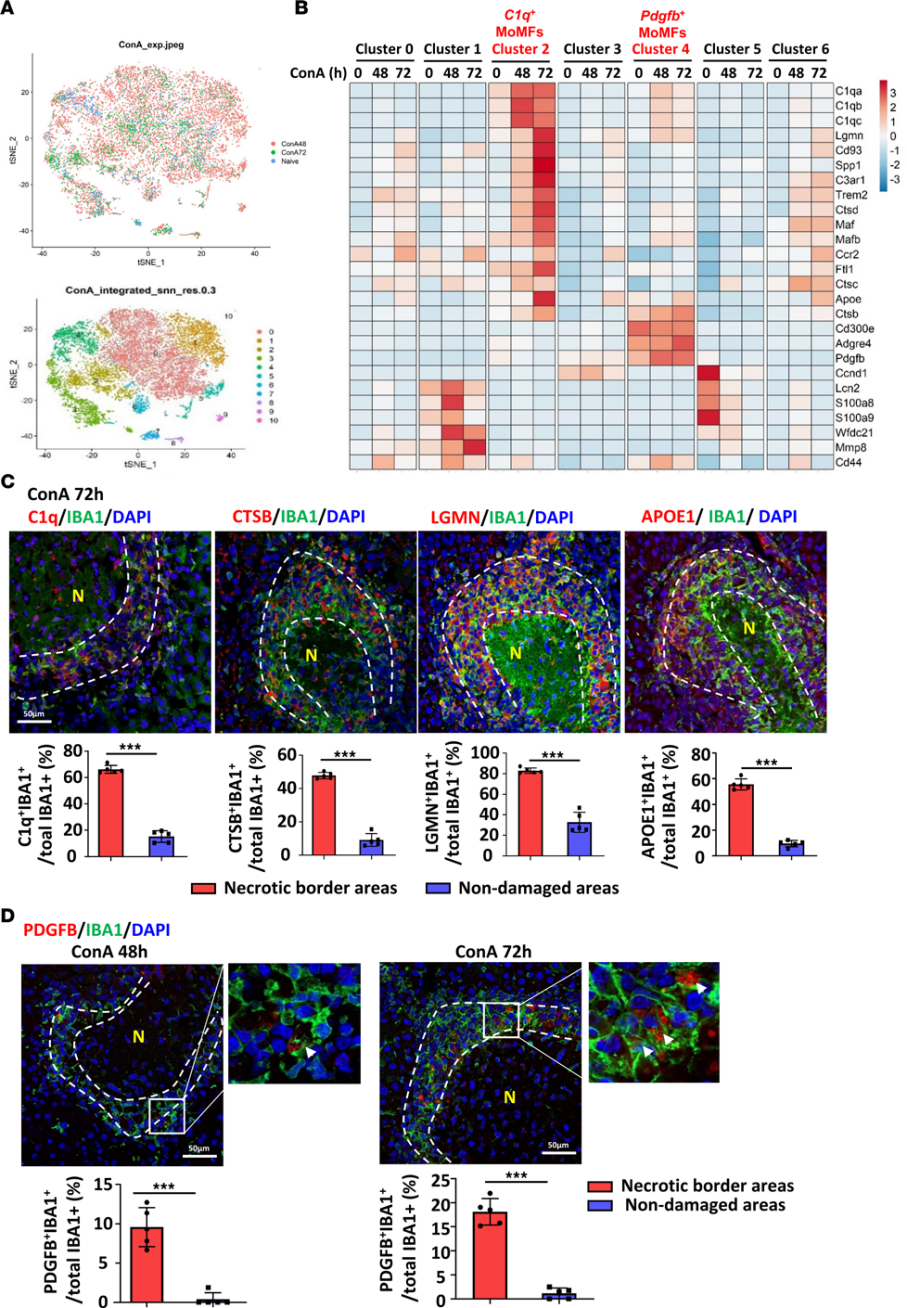
scRNA-Seq identifies 2 clusters of necrosis-associated MoMFs: *C1q*^+^ and *Pdgfb*^+^ MoMFs. (**A** and **B**) Liver MoMFs were isolated from ConA-treated mice (0-, 48-, and 72-hour time points). These cells were subjected to the 10x Genomics Chromium platform for scRNA-Seq. t-SNE plots of cells from naive (1,106 cells), ConA 48 hours after injection (ConA48) (8,541 cells), and ConA 72 hours after injection (3,575 cells) are shown in **A**. Heatmap of the signature genes of *C1q*^+^ macrophages (cluster 2) and *Pdgfb*^+^ macrophages (cluster 4) is shown in **B**. (**C** and **D**) C57BL/6 mice were treated with ConA for 48 or 72 hours. Liver tissues were doubly stained with C1Q/IBA1, CTSB/IBA1, LGMN/IBA1, and APOE/IBA1 (**C**, *n* = 5), and PDGFB/IBA1 (**D**, *n* = 5). Dashed lines indicate the border areas of necrotic regions. Arrowheads indicate IBA^+^ cells with PDGFB expression. Representative images and quantitation are shown. Values in **C** and **D** are represented as means ± SD. Statistical significance was assessed using 2-tailed Student’s *t* test for comparing 2 groups (**C** and **D**). ***P < 0.001.

We then asked whether the MoMFs in the ring-like structure have multiple subsets that may play different roles in facilitating liver-injury repair, so we performed scRNA-Seq by focusing on macrophages. MoMFs (CD45^+^CD11b^+^Ccr2^+^Ly6G^–^) were sorted from the liver 0, 48, and 72 hours after ConA injection (Supplemental Figure 15), and these cells were analyzed by scRNA-Seq. We detected 13,451 genes expressed in 1,106 cells from naive mouse liver MoMFs and 17,361 genes in 8,541 cells and 16,302 genes in 3,575 cells from mouse liver MoMFs 48 and 72 hours after ConA injection, respectively (Figure 6A and Supplemental Figure 16A). We identified 11 clusters using 30 principal components (PCs) under the resolution of 0.3. The majority of the cells are MoMFs (cluster 0 to cluster 6) with some contamination by other immune cells (cluster 7 to 10; T cells, NK cells, NKT cells, innate lymphoid cells [ILCs], and neutrophils, etc.) (Supplemental Figure 16, B and C). Of particular interest were 2 MoMF clusters that were increased by ConA treatment, namely *C1q*^+^ MoMFs (cluster 2) and *Pdgfb*^+^ MoMFs (cluster 4) (Figure 6B and Supplemental Figure 16, D and E).

C1q is well known as playing a key role in removing dead cells (34). Cluster 2 *C1q*^+^ cells also expressed high levels of several genes related to clearance of dead cells and debris such as cathepsin B (*Ctsb*), legumain (*Lgmn*), and apolipoprotein E (*Apoe*) (Figure 6B). The expression of proteins encoded by these genes in IBA1^+^ MoMFs in necrotic border areas was verified by immunofluorescent double staining, while the expression of these proteins in IBA1^+^ Kupffer cells in nondamaged areas was low (Figure 6C).

Among genes of cluster 4, *Pdgfb* encodes PDGFb, a well-characterized growth factor and activator for HSCs (35). Cluster 4 *Pdgfb*^+^ MoMFs also express moderate levels of *C1qa*, *C1qb*, and *C1qc* (Figure 6B) Double-immunofluorescent staining revealed that most PDGFb^+^ cells were IBA1^+^ MoMFs located in perinecrotic ring-like structures, while few PDGFb^+^IBA^+^ Kupffer cells were detected in nondamaged areas (Figure 6D).

Previous studies have reported several populations of MoMFs, including lipid-associated macrophages (LAMs) (36), nonalcoholic steatohepatitis–associated macrophages (NAMs) (37), and scar-associated macrophages (SAMs) (38), that share certain characteristics, such as expression of *Cd9*, *Trem2*, and *Osteopontin* (*Spp1*). Interestingly, *C1q*^+^ MoMFs (cluster 2) expressed some but not all signature genes of these macrophage populations (Supplemental Figure 16F). For example, NAMs/LAMs had more overlapped signature genes with *C1q*^+^ MoMFs than SAMs; however, C*1q*^+^ MoMFs lack several NAM/LAM signature genes (*Cd9*, *Cd36*, *Fabp4*, *Mmp12*, *H2Ab1*) and lack several SAM signature genes (*Il1b*, *Cxcr4*, *Pdgfb*, *Vegfa*, *Hes1*) (Supplemental Figure 16F).

### Hypoxia-reprogrammed C1q^+^ MoMFs promote dead cell clearance and liver lesion resolution.

**Figure 7:**
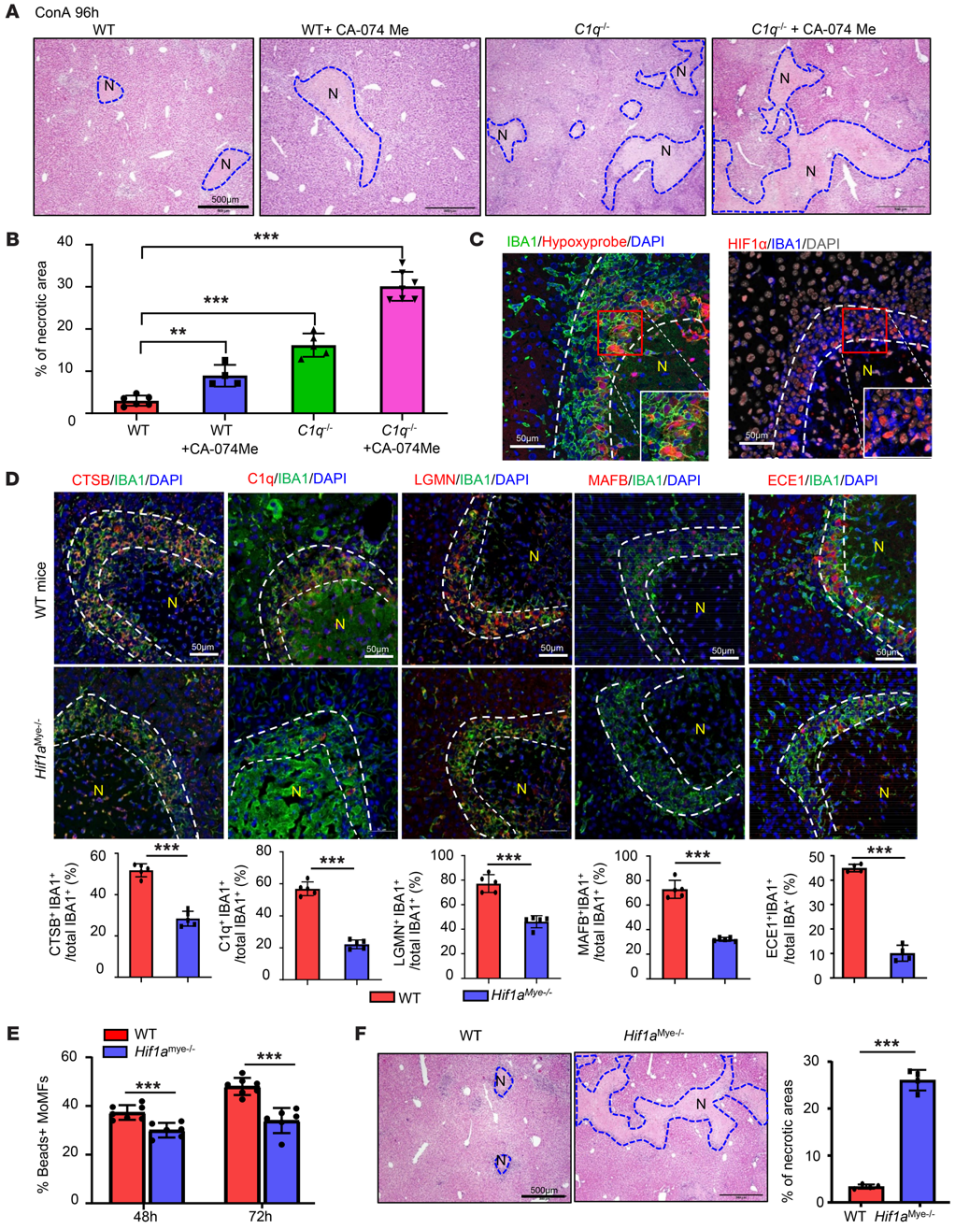
Hypoxia triggers reprogramming of MoMFs in necrotic areas, contributing to the late stages of liver-injury resolution. (**A** and **B**) WT and *C1q^–/–^* mice were treated with ConA, followed by treatment with PBS or CA-074 Me 48 and 72 hours after ConA injection. Liver tissues were collected 96 hours after ConA injection. Representative H&E staining of liver tissue is shown (**A**, *n* = 4-7). Quantification of necrotic area is shown in **B**. (**C**) C57BL/6 mice were treated with ConA for 72 hours. Liver tissues were collected for IBA1/hypoxia probe staining and HIF1α/IBA1 double staining. Representative images are shown (*n* = 5). (**D**) WT and *Hif1a^mye–/–^* mice were treated with ConA for 72 hours. Liver tissues were collected for double staining with various antibodies, as indicated (*n* = 5). Quantification of the percentage of positive cells is shown. (**E**) MoMFs from ConA-treated mice were isolated for bead uptake assay (*n* = 6–7). (**F**) WT and *Hif1a^mye–/–^* mice were treated with ConA for 96 hours. Liver tissues were collected for H&E staining, and quantification of necrotic areas is shown on the right (*n* = 4). Dashed lines indicate the borders of necrotic areas. Values in **B**, **D**, **E**, and **F** are represented as means ± SD. Statistical significance was assessed using 2-tailed Student’s *t* test for comparing 2 groups (**D**–**F**) and 1-way ANOVA followed by Tukey’s post hoc test for multiple groups (**B**). **P < 0.01; ***P < 0.001.

The cluster 2 *C1q*^+^ MoMFs located in ring-like structures express complements and proteinases, such as *C1q* and *Ctsb*. To test how the proteins encoded by these genes contribute to liver damage recovery, we used *C1q^–/–^* mice and Ctsb inhibitor. Compared with WT mice, *C1q^–/–^* mice or WT mice treated with a Ctsb inhibitor (CA-074 Me) showed delayed necrosis resolution, as reflected by the larger necrotic areas detected in these mice compared with those in vehicle-treated WT mice (Figure 7, A and B). CA-074 Me treatment further delayed liver repair in *C1q^–/–^* mice, with the largest necrotic areas compared with those in all other groups (Figure 7, A and B).

Because necrotic areas are strongly associated with hypoxia, we hypothesized that hypoxia plays a role in inducing the formation of the cluster of *C1q*^+^ MoMFs. Indeed, hypoxia in MoMFs as well as other cells in the necrotic area was visualized by hypoxyprobe, and HIF1α was strongly activated in these IBA1^+^ MoMFs in necrotic border areas, but not in IBA1^+^ Kupffer cells in nondamaged areas, as detected by immunostaining (Figure 7C).

Expression of CTSB and ECE1, 2 well-known targets of HIF1α (39, 40) was significantly reduced in IBA1^+^ MoMFs from myeloid cell–specific *Hifa*-knockout (*Hif1a^mye–/–^*) mice compared with those from WT mice after ConA treatment (Figure 7D). Expression of C1q and LGMN, which are not direct targets of HIF1α, was also markedly reduced in MoMFs from *Hif1a^mye–/–^* mice (Figure 7D). Expression of MAFB, a transcription factor that is an upstream regulator of C1q (41), was markedly reduced in MoMFs from *Hif1a^mye–/–^* mice (Figure 7D).

To address whether hypoxia is sufficient to induce the phenotype change of MoMFs, we treated bone marrow–derived macrophages (BMDMs) in vitro with an HIF inducer, CoCl_2_ (100 μM) (42) and/or dead hepatocytes to mimic a necrotic environment. As shown in Supplemental Figure 17, CoCl_2_ treatment alone upregulated most of the signature genes of clusters 2 and 4, while the addition of dead hepatocytes into CoCl_2_-treated BMDMs further upregulated the expression levels of several genes, including *C1qa*, *C1qb*, and *C1qc*. Treatment with dead hepatocytes alone also upregulated several genes in BMDMs.

MoMFs from *Hif1a^mye–/–^* mice had reduced expression of several genes related to clearance of dead cells, which may affect the phagocytosis function of MoMFs. Accordingly, liver MoMFs isolated from ConA- treated *Hif1a^mye–/–^* mice showed impaired phagocytosis compared with those from WT mice (Figure 7E). Finally, *Hif1a^mye–/–^* mice displayed delayed necrosis resolution compared with WT mice after ConA treatment (Figure 7F). Collectively, these data suggest that hypoxia-driven *C1q*^+^ MoMFs facilitate dead cell removal and promote liver lesion repair.

### PDGFB derived from MoMFs in necrotic area promotes HSC activation and contraction, thereby accelerating liver lesion resolution.

**Figure 8:**
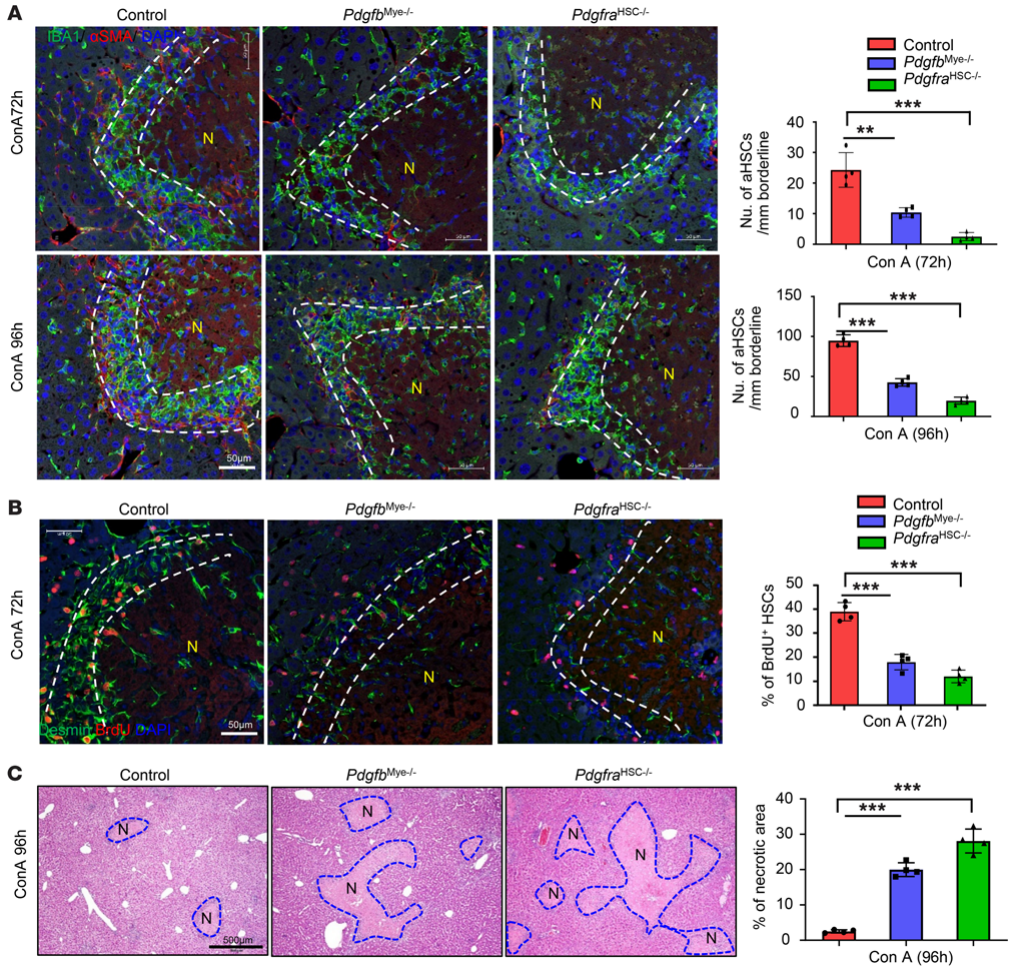
Myeloid cell–derived PDGFB promotes HSC proliferation and activation. WT, *Pdgfb^mye–/–^*, and *Pdgfra^HSC–/–^* mice were treated with ConA for 72 or 96 hours. BrdU was given 2 hours before sacrifice. Liver tissues were stained with (**A**) IBA1 and α-SMA (*n* = 4), (**B**) BrdU and desmin (*n* = 4), and (**C**) H&E (*n* = 4). Numbers of aHSCs and BrdU^+^ HSCs in the border of necrotic areas were quantified and are shown on the right. Dashed lines indicate the border areas of necrotic regions. Values in **A**–**C** are represented as means ± SD. Statistical significance was assessed using 1-way ANOVA followed by Tukey’s post hoc test for multiple groups (**A**–**C**). **P < 0.01; ***P < 0.001.

PDGF acting via PDGFR is well known to activate HSCs (35). The above data show that PDGFB is expressed at high levels in MoMFs that surround the necrotic areas. Interestingly, the PDGFB receptor PDGFR was also detected at high levels in HSCs in the same perinecrotic zone (Supplemental Figure 18A). To determine the contribution of MoMF-derived PDGFB in HSC activation, we generated myeloid cell–specific *Pdgfb*-knockout (*Pdgfb^mye–/–^*) mice and HSC-specific PDGF receptor *Pdgfra*-knockout (*Pdgfra^HSC–/–^*) mice. As illustrated in Figure 8A, the number of activated α-SMA^+^ HSCs that overlapped with IBA1^+^ MoMFs in the perinecrotic area was significantly lower in *Pdgfb^mye–/–^* and *Pdgfra^HSC–/–^* mice than in WT mice at the late stage of liver-injury repair. The number of total desmin^+^ HSCs was also lower in both knockout mice than in WT mice (Supplemental Figure 18B). Finally, the number of Desmin^+^BrdU^+^ proliferating HSCs was much lower in *Pdgfb^mye–/–^* and *Pdgfra^HSC–/–^* mice than in WT mice (Figure 8B). In contrast, the number of IBA1^+^CLEC4F^–^ MoMFs on the border areas was comparable in WT, *Pdgfb^mye–/–^*, and *Pdgfra^HSC–/–^* mice, and the number of IBA1^+^CLEC4F^+^ Kupffer cells in nondamaged areas was also comparable in all 3 groups (Supplemental Figure 19). Moreover, we evaluated the efficiency of liver damage resolution and HSC contraction signals in these mice after ConA treatment. The necrotic areas were much larger in *Pdgfb^mye–/–^* and *Pdgfra^HSC–/–^* mice than in WT mice after ConA treatment (Figure 8C). Furthermore, immunostaining of the contraction signals YAP and pMLC was much weaker in *Pdgfb^mye–/–^* and *Pdgfra^HSC–/–^* mice than in WT littermates after ConA treatment (Supplemental Figure 20). Finally, we determined whether induction of *pdgfb*^+^ MoMFs was due to hypoxia by immunostaining of PDGFβ in ConA-treated WT and *Hif1a^mye–/–^* mice. As illustrated in Supplemental Figure 21, reduced PDGFb expression was found in MoMFs from *Hif1a^mye–/–^* mice, which is consistent with several reports that PDGFβ is upregulated by hypoxia (43, 44). These data suggest that hypoxia promotes the recruitment of MoMFs in the perinecrotic zone, thus playing a key role in inducing HSC activation/contraction.

### Aggregation of IBA1^+^ MoMFs, SOX9^+^ hepatocytes, and αSMA^+^ HSCs surrounding necrotic lesions is also detected in other liver-injury models.

To determine whether the interplay of MoMFs, SOX9^+^ hepatocytes, and aHSCs also applies to other models of liver injury, we performed immunohistochemistry analyses of IBA1, SOX9, and α-SMA in several liver-injury models induced by *Klebsiella pneumoniae* (*KP*) infection, ischemia/reperfusion (I/R), or hepatic toxins (carbon tetrachloride [CCl_4_], and APAP). As illustrated in Supplemental Figure 22, significant aggregation of IBA^+^ MoMFs, SOX9^+^ hepatocytes, and α-SMA^+^ HSCs was detected surrounding the liver necrotic areas in the *KP* infection model and I/R model. However, such aggregation was less evident in CCl_4_ and APAP models, in which IBA1^+^ macrophages and αSMA^+^ HSCs were found inside the necrotic area. IBA1^+^ macrophages/Kupffer cells were also detected in noninjured areas of CCl_4_ and APAP models. Collectively, the interplay of MoMFs, SOX9^+^ hepatocytes, and aHSCs likely also plays an important role in promoting necrotic liver lesion resolution in KP or I/R models, while direct hepatotoxin-induced (such as CCl_4_, APAP) liver lesion repair uses a different mechanism due to hepatocyte dominant injury in these drug-induced injury models (9). These hepatotoxins themselves are not toxic; rather their metabolites generated via the CYP2E1 promoted hepatocyte injury. CYP2E1s are mainly expressed in hepatocytes near the central vein, resulting in necrosis around the central vein. In contrast, hepatocytes near the portal area, which express very low levels of CYP2E1, are resistant to APAP-induced liver injury (45). Thus, no cell–death resistant SOX9^+^ hepatocytes are required for preventing further injury.

## Discussion

**Figure 9:**
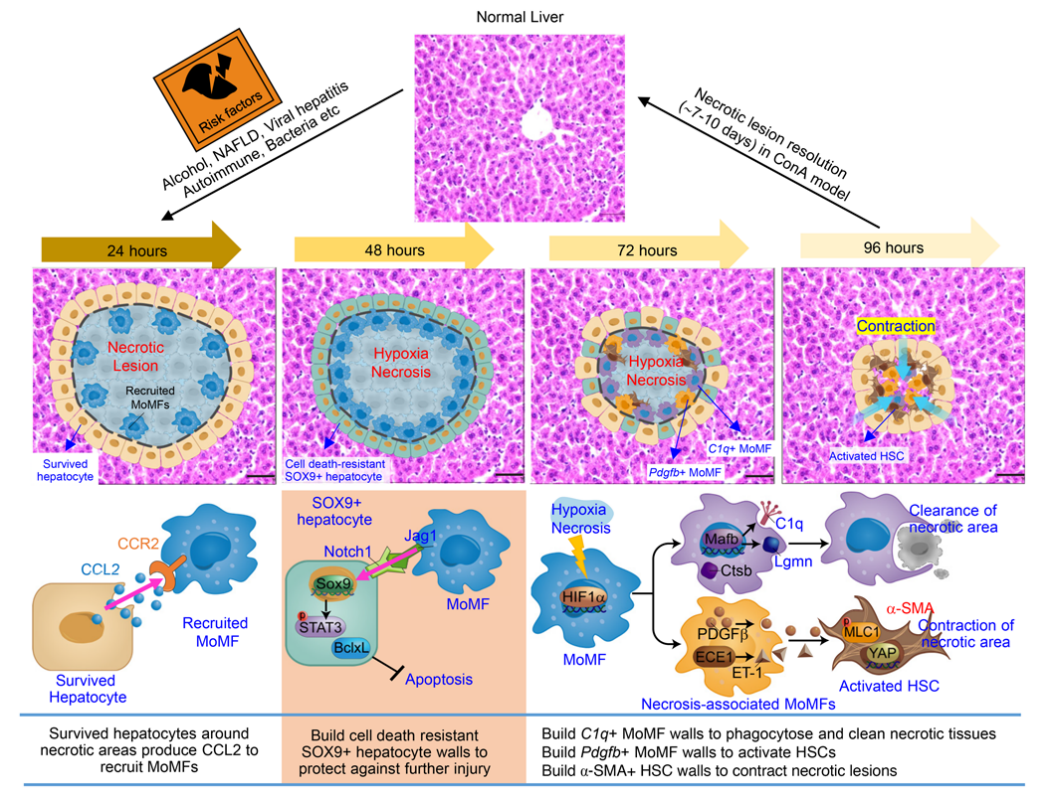
A model depicting the dynamic changes of MoMFs and their interaction with other cells, promoting liver necrotic lesion resolution. The liver has a great ability to repair and eliminate necrotic lesions after acute immune-mediated liver injury. At the early stage of liver injury, JAG1^+^ MoMFs, which are induced by hepatocyte-derived CCL2, help build cell death–resistant SOX9^+^ hepatocyte walls to encapsulate the necrotic areas and subsequently protect the nondamaged hepatocytes from further injury. At the later stage, necrotic environment (hypoxia and dead hepatocytes) induces *C1q^+^* MoMFs and *Pdgfb*^+^ MoMFs to encapsulate the necrotic lesions. *C1q^+^* MoMFs play an important role in removing dead cells and necrotic tissues, while *Pdgfb*^+^ MoMFs induce activation of α-SMA^+^ HSCs that squeeze the capsule of the necrotic lesions to facilitate their elimination. Hepatocytes in nondamaged areas proliferate and expand, which may further help contract the capsule of the necrotic lesions. NAFLD, nonalcoholic fatty liver disease.

Necrotic lesions of the liver may develop in response to a variety of pathologic insults, but the cellular mechanisms involved in their resolution and repair have remained largely unknown. Research in the last three decades has revealed that macrophages are highly adaptable in their ability to respond to a variety of threats to the organism by differentiating into subsets with unique gene expression profiles, exerting multiple functions, including promoting liver-injury repair (11–13, 20). In the current study, we found that MoMFs are rapidly recruited to the necrotic lesions after immune-mediated acute liver injury. We further characterized the dynamic changes of MoMFs and their interactions with several other cell types in the liver necrotic lesions and identified at least 3 specific roles played by MoMFs in resolving necrotic lesions, which are summarized in a working model in Figure 9.

The first reparative role of MoMFs in this injury model is to induce a wall of cell death–resistant SOX9^+^ hepatocytes that encapsulate the necrotic lesions, thereby protecting undamaged hepatocytes from further injury. At the early stage of liver injury, MoMFs rapidly aggregate along the perimeter of the necrotic areas. This rapid recruitment is dependent on the interaction of hepatocyte-derived CCL2 with macrophage CCR2 because liver-specific deletion of *Ccl2* or global deletion of its receptor *Ccr2* markedly reduced the recruitment of SOX9^+^ hepatocytes. The important roles of CCL2/CCR2 in protecting against ConA-induced liver injury have been previously well documented (18, 19). Interestingly, only the hepatocytes around the perimeter of the necrotic area express high levels of CCL2, which may be due to the activation of NF-κB stimulated by danger-associated molecule patterns (DAMPs) (46) released by the necrotic tissue. Indeed, activation of NF-κB, which upregulates CCL2 (22), is detected at higher levels in perinecrotic hepatocytes compared with hepatocytes in undamaged areas. Furthermore, the important role of MoMFs in inducing SOX9^+^ hepatocytes is strongly supported by the notion that the number of SOX9^+^ hepatocytes was markedly reduced after macrophage depletion or in *Ccr2*^–/–^ mice. SOX9 is mainly expressed in biliary ductular cells and LPCs, but not in mature hepatocytes (23), playing a critical role in controlling specification of hepatoblasts to BDCs via the activation of NOTCH (24, 25). In the current study, we found that the NOTCH signaling activated by its ligand JAG1 is also involved in the induction of SOX9^+^ hepatocytes in the ConA liver-injury model.

The characteristics and functions of SOX9^+^ hepatocytes were further investigated in our study, which suggests that SOX9^+^ hepatocytes play an important role in protecting against immune-mediated liver injury but do not contribute to liver regeneration. First, we found that SOX9^+^ hepatocytes around necrotic regions did not proliferate, while SOX9^–^ hepatocytes in nondamaged areas proliferated, indicating SOX9^+^ hepatocytes do not contribute to hepatocyte expansion in this ConA-induced liver-injury model. Second, we found that SOX9^+^ hepatocytes expressed high levels of activated STAT3 and its downstream antiapoptotic protein BCL-xL. Previous studies have indicated that NOTCH signaling and its downstream target Hes5 promote the activation of STAT3 by facilitating complex formation between JAK2 and STAT3 (47). This is consistent with our observation of high NOTCH and pSTAT3 levels in SOX9^+^ hepatocytes. Third, the resistance to apoptosis of SOX9^+^ hepatocytes was further confirmed by our finding of prolonged liver injury in *Sox9^Hep–/–^* mice following ConA injection. The protective effect of SOX9 is likely mediated via the induction of pSTAT3 and BCL2, as SOX9^+^ hepatocytes express higher levels of pSTAT3 and BCL-2 than SOX9^–^ hepatocytes, which is in agreement with a previous report that SOX9^+^ LPCs are resistant to cytokine-induced apoptosis (48). To summarize, the rapidly infiltrating macrophages express high levels of JAG1 to induce SOX9^+^ hepatocytes that form a “shell-like” capsule surrounding the necrotic lesions, thereby protecting against further injury.

The second important reparative role of MoMFs in ConA-induced liver injury is to remove the necrotic tissues to help resolve the lesions. Phagocytosis is the major function of macrophages, so it is expected that MoMFs play a critical role in clearing the necrotic cells, as their depletion markedly delayed necrotic lesion resolution. By using scRNA-Seq, we have identified a cluster of *C1q*^+^ macrophages that express several genes related to removal of necrotic tissues, such as *C1q*, *Cts*, *Lgmn*, and *Ftl1*. C1q can act as a bridging molecule that binds to apoptotic cells and promotes their phagocytosis by macrophages (49). Cathepsins (CTS) encoded by *Cts* genes belong to a proteinase family located in lysosomes and are considered very important in the degradation of both intracellular proteins and the extracellular matrix (50). LGMN, encoded by *Lgmn*, is another lysosomal proteinase that can activate cathepsins, degrade the extracellular matrix, and promote the clearance of apoptotic cells (51). Intriguingly, immunofluorescent staining analyses demonstrated that *C1q*^+^ macrophages were mainly located on the margins of necrotic lesions, and they coexpressed C1Q, CTS, and LGMN. Genetic deletion of *C1q* and/or inhibition of CTS markedly delayed liver necrosis resolution, suggesting an important role of *C1q*^+^ macrophages in removing necrotic tissues and promoting liver repair. Furthermore, we explored how *C1q*^+^ macrophages are induced during acute liver injury. Because *C1q*^+^ macrophages are located in the hypoxic milieu of the areas surrounding necrotic lesions, we hypothesized that hypoxia plays a role in inducing *C1q*^+^ macrophages. This hypothesis is indeed supported by our data demonstrating that Hydroxyprobe and HIF1α are markedly elevated in MoMFs located in perinecrotic areas and that deletion of *Hif1a* abolished C1q expression and delayed necrotic lesion resolution. Although C1q is markedly reduced in *Hif1a^mye–/–^* mice, sequence analysis did not identify HIF1α-binding sites on the *C1q* promoter, suggesting that *C1q* is not a direct target of HIF1α. Furthermore, our scRNA data revealed that *C1q*^+^ macrophages express MAFB, a key transcription factor that controls C1q expression (41), and expression of MAFB was markedly diminished in MoMFs in *Hif1a^mye–/–^* mice, suggesting that HIF1α controls C1q expression by inducing MAFB in MoMFs. In addition, in vitro experiments also support a role of necrotic environment (hypoxia and necrosis) in inducing *C1q*^+^ macrophages, as in vitro treatment of macrophages with an HIF1 inducer (CoCl_2_) and dead hepatocytes markedly upregulated most of the *C1q*^+^ MoMF signature genes. Finally, this *C1q*^+^ MoMF population expresses some but not all signature genes of several previously reported macrophage populations, such as LAMs (36), NAMs (37), and SAMs (38). These differences are likely due to the plasticity of macrophages that enables them to react to different microenvironments. In the ConA-induced liver-injury model we used in this study, massive necrosis occurred to generate large amounts of necrotic tissues in necrotic lesions. C*1q*^+^ MoMFs express many signature genes that play a crucial role in promoting clearance of necrotic tissues, but lack several lipid metabolism–associated signature genes (*Cd36*, *Fabl4*) that are observed in NAMs/LAMs from fatty liver disease and lack fibrosis-associated genes (*Il1b*, *Cxcr4*, *Pdgfb*, *Vegfa*, *Hes1*) that are observed in SAMs from cirrhotic livers. It is also notable that hypoxia and HIF2 have been shown to reprogram liver macrophages in acute liver responses to APAP (52). Collectively, necrosis-associated hypoxia activates the HIF1α/MAFB/C1q axis in MoMFs, resulting in accumulation of *C1q*^+^ MoMFs with strong phagocytic activity to remove necrotic tissues.

The third important reparative role of MoMFs is manifested at the later stages of liver injury when they activate α-SMA^+^ HSCs that squeeze the capsule of the necrotic lesions to facilitate their elimination. scRNA-Seq and immunohistochemical analyses identified a cluster of *Pdgfb*^+^ MoMFs that are aggregated and colocalized with α-SMA^+^ HSCs on the necrotic perimeter. *Pdgfb*^+^ MoMFs are critical for the formation of α-SMA^+^ HSCs, as knockout of *Pdgfb* in MoMFs or its receptor *Pdgfrb* in HSCs abolished HSC proliferation and activation. An important function of α-SMA^+^ HSCs is to squeeze the capsule of necrotic lesions, ultimately eliminating them. First, during necrotic lesion resolution, no hepatocytes or LPCs were detected inside the necrotic areas, while hepatocyte proliferation was evident in undamaged parenchymal regions, suggesting that hepatocytes in undamaged regions proliferate and expand, with the result of squeezing the necrotic lesions. Second, strong contraction signals (such as YAP and pMLC) (31, 32) were detected in α-SMA^+^ HSCs located in the perinecrotic areas. Third, ECE1 was detected at high levels in perinecrotic MoMFs, especially at late time points of injury. ECE1 is a key membrane-bound metalloendopeptidase that cleaves ET-1 to biologically active peptides that play a key role in inducing HSC contraction (32, 33). Taken together, these data show that MoMFs not only produce PDGFb to activate HSCs, but also express ECE1 that cleaves ET-1 into active form, which likely plays a key role in promoting α-SMA^+^ HSC contraction and subsequent elimination of necrotic lesions.

Kupffer cells, liver-resident macrophages, have been shown to play an important role in initiating ConA-induced liver injury by producing a variety of proinflammatory cytokines, as depletion of Kupffer cells before ConA injection diminished ConA-induced liver injury (17, 21). However, the functions of Kupffer cells after injury have not been well studied. Our current studies suggest that Kupffer cells play a less important role in promoting necrotic lesion resolution than infiltrating MoMFs. In necrotic areas, Kupffer cells were lost after injury, probably due to cell death, while in nondamaged areas, Kupffer cells do not migrate and do not undergo hypoxic changes similar to those of MoMFs, which are rapidly recruited to necrotic borders. Indeed, induction of most MoMF signature genes and proteins was not observed in Kupffer cells, as demonstrated by immunostaining and RT-qPCR analysis. However, as professional phagocytic cells, Kupffer cells may still contribute to removing necrotic tissues leaked from the necrotic areas after ConA injection, which will require further studies to confirm. In addition, both Kupffer cell proliferation and MoMF differentiation have been shown to contribute to Kupffer cell restoration in several liver injury models (53–55). We also observed substantial Kupffer cell proliferation in nondamaged areas after ConA injection, which likely contributes to the restoration of the lost Kupffer cells in necrotic areas in this acute liver-injury model. Future lineage-tracing experiments are needed to clarify whether MoMFs also contribute to Kupffer cell restoration in the ConA-induced liver-injury model.

In summary, here we have demonstrated that MoMFs play a key role in repairing liver necrotic lesions in a ConA-induced liver-injury model by orchestrating the sequential induction of multiple cell types with unique gene expression profiles, including SOX9^+^ hepatocytes and aHSCs. Interestingly, we also observed similar aggregation of MoMFs, SOX9^+^ hepatocytes, and aHSCs in liver-injury models induced by bacterial infection or ischemia/reperfusion, suggesting such interplay plays an important role in promoting liver necrotic lesion repair in these models. Future studies to identify the complex roles of MoMFs in repairing liver necrotic lesions may help the design of better macrophage-based therapies for the treatment of liver diseases. Indeed, such therapies have been tested for the treatment of liver diseases both in preclinical models and in patients (13, 56, 57).

## Methods

### Mouse strains.

C57BL/6J (catalog 000664), *Sox9-CreERT2* (catalog 018829), *Sox9^fl/fl^* (catalog 013106), *Notch1^fl/fl^* (catalog 007181), *Notch2^fl/fl^* (catalog 010525), *Ccr2RFP* (catalog 017586), *Ccl2RFP^fl/fl^* (catalog 016849), *Pdgfb^fl/fl^* (catalog 017622), *Pdgfra^fl/fl^* (catalog 006492), *LysMcre* (catalog 018956), *CD11cCre* (catalog 008068), *C1qa*^–/–^ (catalog 031675), *AlbCre* (catalog 003574), and ROSA-EYFP reporter (catalog 006148) mice were obtained from The Jackson Laboratory. *Hif1*α*^fl/fl^* mice were described previously (52). *LratCre* mice were provided by Robert F. Schwabe (Columbia University, New York, New York, USA) (58). *ihCD59* mice were described previously (59). All mice used in this study were 6- to 10-week-old males.

### ConA-induced liver-injury model.

ConA (Sigma-Aldrich) was dissolved in PBS and injected i.v. through the tail vein at a dose of 12 mg/kg. Mouse serum was obtained at different times after ConA injection. DBZ (Tocris Bioscience) dissolved in DMSO was suspended in PBS containing 0.5% (w/v) hydroxypropylmethylcellulose (Methocel E4M) (Sigma-Aldrich) and 0.01% Tween 80 (Sigma-Aldrich). DBZ was injected 8 and 24 hours after ConA treatment at a dose of 30 μmol/kg body weight. ALT activities were determined using an IDEXX Chemistry Analyzer System (IDEXX Laboratories). BrdU (50 mg/kg body weight) was given by i.p. injection 2 hours before sacrifice. The necrotic areas were quantified by ImageJ (NIH) (60).

### Treatment of mice.

In some ConA-treated mice, 5 mg/kg CA-074 Me (a specific inhibitor of cathepsin B) (Selleck Chemicals, diluted in 5% DMSO containing PBS) or vehicle was administrated by i.p injections daily and before sacrifice.

### Macrophage depletion.

To deplete liver macrophages, mice received 150 μL clodronate liposomes or control liposomes (FormuMax Scientific) daily by i.v. injections.

### Cell-fate tracing and hepatocyte-specific gene knockout.

To track BDCs/LPCs, we crossed *Sox9-CreERT2* mice with *ROSA-EYFP* reporter mice. To achieve Cre-LoxP recombination, mice were injected i.p. with tamoxifen (catalog T5648, Sigma-Aldrich) (dissolved in corn oil at a concentration of 10 mg/ml) at 50 mg/kg body weight every 3 days for 3 times. Mice were used for experiments 1 week after the last injection to ensure washout of tamoxifen. To label hepatocytes alone, ROSA-EYFP reporter mice were infected with AAV8-TBG-Cre (1.3 × 10^11^ genome copies per mouse, Penn Vector Core, Philadelphia, Pennsylvania, USA) by i.v. injection. Mice were treated with ConA 10 days after AAV8-Tbg-Cre injection. To specifically knock out genes in hepatocytes, floxed mice (*Sox9*, *Notch1*, *Notch2*) were infected with AAV8-TBG-Cre and were treated with ConA 10 days after AAV8-TBG-Cre injection.

### Isolation of hepatic mononuclear cells.

Mouse livers were removed and pressed through a 70 μm cell strainer. The liver cells were suspended in PBS and centrifuged at 50*g* for 5 minutes. Supernatants containing mononuclear cells (MNCs) were collected, washed in PBS, and resuspended in 40% Percoll medium (GE Healthcare). The cell suspension was gently overlaid onto 70% Percoll and was centrifuged for 30 minutes at 750*g*. MNCs were collected from the interphase, washed twice in PBS, counted, and resuspended in PBS for flow cytometry assays.

### Flow cytometry analysis.

Single-cell suspension of liver or spleen MNCs was washed in PBS containing 1% bovine serum albumin. The cells were surface stained with fluorochrome-conjugated monoclonal antibody for 30 minutes on ice. The antibodies used were anti-CD3 (clone 145-2C11), anti-CD19 (clone 1D3), anti-NK1.1(clone PK136), anti-Ly6G (clone 1A8-Ly6g), anti-CD11b (clone M1/70), anti-CD11c (clone N418), anti-CD80 (clone 16-10A1), anti-CD86 (clone GL1), anti–MHC-II (clone M5/114.15.2), anti-JAG1 (clone HMJ1-29), anti-CD115 (clone AFS98), anti-Ly6C (clone HK1.4), anti-F4/80 (clone BM8), anti-MERTK (clone DS5MMER), anti-CD209 (clone LWC06), and anti-CD206 (clone MR6F3) (eBioscience); anti-CX3CR1(clone SA011F11), ant-CCR5 (clone HM-CCR5), anti-CD64 (clone X54-5/7.1), anti-CD36 (clone HM36), anti-TIM4(clone RMT4-54), anti-CD9(clone MZ3), anti–CLEC-2(clone 7D9/CLEC-2), and anti-CCR2(clone SA203G11) (BioLegend); and anti–LAP–TGF-β (clone TW7-16B4) (BD Biosciences). Samples were acquired on a FACSCalibur flow cytometer (BD Biosciences) or Cytoflex flow cytometer (Beckman Coulter), and data were analyzed using FlowJo software, version 7.6.5 (TreeStar).

### JAG1^+^ cell enrichment.

Hepatic MNCs were incubated with PE-labeled JAG antibody (clone HMJ1-29, eBioscience) for 30 minutes on ice. Anti-PE magnetic beads (Miltenyi Biotec) were added to the stained cells. JAG1^+^ cells were enriched by using LS column (Miltenyi Biotec).

### scRNA-Seq.

The liver samples were homogenized with a gentleMACS Dissociator (Miltenyi), filtered through 70 μm cell strainers, and then centrifuged at 400*g* through a Percoll density gradient (40% and 70%). Immune cells were collected and further sorted with BD FACSMelody to isolate CD45^+^CD11b^+^Ccr2^+^Ly6G^–^ liver MoMFs.

The sorted MoMFs from mouse livers were washed twice with PBS. The cell concentration and viability were assessed using the LUNA-FL Dual Fluorescence Cell Counter (Logos Biosystems). The sorted MoMFs were loaded in the lanes according to the 10x Genomics 3′ Single Cell User Guide with a single capture lane per sample targeting recovery of 6,000 cells per lane. Cell partitioning was completed successfully with uniform emulsion consistency, and reverse-transcription PCR was run overnight. All subsequent steps of library preparation and quality control were performed using 10× Chromium Next GEM Single Cell 3′ Kit according to the manufacturer’s instructions. Sequencing was performed on an Illumina NextSeq 500 at the National Cancer Institute Center for Cancer Research Genomics Core (Bethesda, Maryland, USA). All samples had excellent sequencing yield. The Q30 bases RNA reads were above 86%. The FASTQ files and data processing were performed using 10x Genomics CellRanger pipeline (version 6.0.0). Sequenced reads were aligned to the 10x Genomics mouse reference sequence (refdata-gex-mm10-2020-A).

Seurat package (version 4.0) was used to further analyze scRNA-Seq data (61). After removing dying cells with high mitochondria genes, doublets, and cells with low quality, 13,222 cells that expressed more than 500 unique molecular identifier (UMI) counts were used for further analysis. UMI counts for each gene were normalized, and highly variable genes were identified via the SCTransform function using default parameters (62). The data were integrated with the IntegrateData function of the Seurat package. The first 30 PC and resolution 0.3 were used for the clustering via FindNeighbors and FindClusters functions. Differential gene expression was assessed using the FindMarker function. The t-SNE plots, violin plots, dot plot, feature plots, and heatmaps were generated by R.

### Immunohistochemical staining in the liver.

After heat-induced epitope retrieval in citrate buffer (pH 6.0), paraffin-embedded sections were stained with primary antibodies as listed in Supplemental Table 1 and visualized by DAB or AEC. For pSTAT3 staining, sections were stained after heat-induced epitope retrieval in 1 mM EDTA (pH 8.0). BrdU staining was determined by immunostaining with the BrdU In-Situ Detection Kit (catalog 551321, BD Biosciences)

### Immunofluorescence staining.

Mice were perfused with 4% PFA; tissues were obtained and fixed in 4% PFA for 2 additional hours and immersed in 30% sucrose for 2 days. Tissues were embedded in OCT compound and sectioned (10 μm). Sections were stained with primary antibodies listed in Supplemental Table 1. Fluorescent-labeled secondary antibodies (CST) were used to visualize the primary antibodies. The images were obtained by using an LSM 710 confocal microscope (Zeiss).

### Hypoxia visualization.

Tissue hypoxia was visualized by the Hypoxyprobe Biotin Kit according to the manufacturer’s instructions. Briefly, mice were treated with 60 mg/kg (i.p) pimonidazole HCl. Tissues were collected 1 hour later and stained with antibody provided in the kit.

### Multiplex immunofluorescence staining.

Multiplex immunofluorescence staining with more than 3 markers was performed as previously described (63). Acquired images were processed and analyzed using Image J FIJI (60) and the FIJI plugin HyperStackReg V5.682, Ilastik (version 1.3.3post3) (64), and CellProfiler (version 4.2.4) (65) for cell counting and quantification as previously described (66, 67) (Supplemental Figure 23).

### TUNEL staining.

TUNEL staining on liver sections was performed using the ApopTag Peroxidase In Situ Apoptosis Detection Kit (catalog S7100, Sigma-Aldrich) according to the manufacturer’s instruction.

### Phagocytosis assay.

The phagocytosis assay was performed by using a kit from Cayman Chemical. Briefly, after surface marker staining, MoMFs were incubated with FITC-labeled latex beads for 30 minutes at 37°C. After washing, the cells were analyzed using flow cytometry.

### RT-qPCR.

Total RNA was purified from MoMFs or liver tissues using TRIzol Reagent (Thermo Fisher Scientific) according to the manufacturer’s instructions. RNAs (1 μg of total) were reverse transcribed into cDNA using a High-Capacity cDNA Reverse Transcription Kit (Thermo Fisher Scientific). Expression levels of mRNA were measured by RT-qPCR using an ABI7500 RT-PCR system (Applied Biosystems). 18S rRNA was used as the housekeeping control gene. Primers used for RT-qPCR are listed in Supplemental Table 2.

### Statistics.

Results are expressed as means ± SD. All statistical analyses were performed using GraphPad Prism software (version 7.0a). To compare values obtained from 2 groups, Student’s *t* test was performed. Data from multiple groups were compared with 1-way ANOVA followed by Tukey’s post test as appropriate. *P* < 0.05 was considered significant.

### Study approval.

Animal care and experiments were conducted under the guidelines and protocols approved by the NIAAA Animal Care and Use Committee. All animals were cared for in accordance with NIH guidelines.

### Data availability.

scRNA-Seq data were deposited in the NCBI’s Gene Expression Omnibus database (GEO GSE229498). Values for all data points found in graphs can be found in the supporting data values file.

## Author contributions

DF and XX designed and performed most mouse model experiments and wrote the paper. YG conducted some mouse model experiments and analyzed the scRNA-Seq data. AG helped perform the multiplex immunofluorescent staining experiments and quantitate the images. CC and XWW performed scRNA-Seq experiments and analyzed scRNA-Seq data. HL, YH, HW, and HP conducted some immunofluorescent staining and mouse model experiments. CJ, SPC, FT, and GK helped with data analysis, provided relevant intellectual input, and edited the manuscript. BG obtained funding, supervised the whole project, and wrote the paper. All authors approved the final manuscript.

## Supplementary Material

Supplemental data

Supporting data values

## Version 1 06/20/2023

In-Press Preview

## Version 2 08/01/2023

Electronic publication
