# Supplementary material for: Monocyte-derived macrophages orchestrate multiple cell-type interactions to repair necrotic liver lesions in disease models
Source: J Clin Invest. 2023 Aug 1;133(15):e166954. doi: 10.1172/JCI166954 (PMC10378165; doi:10.1172/JCI166954)
Supplement: Supplemental data [file jci-133-166954-s037.pdf]

Supplemental Figure 1A-C

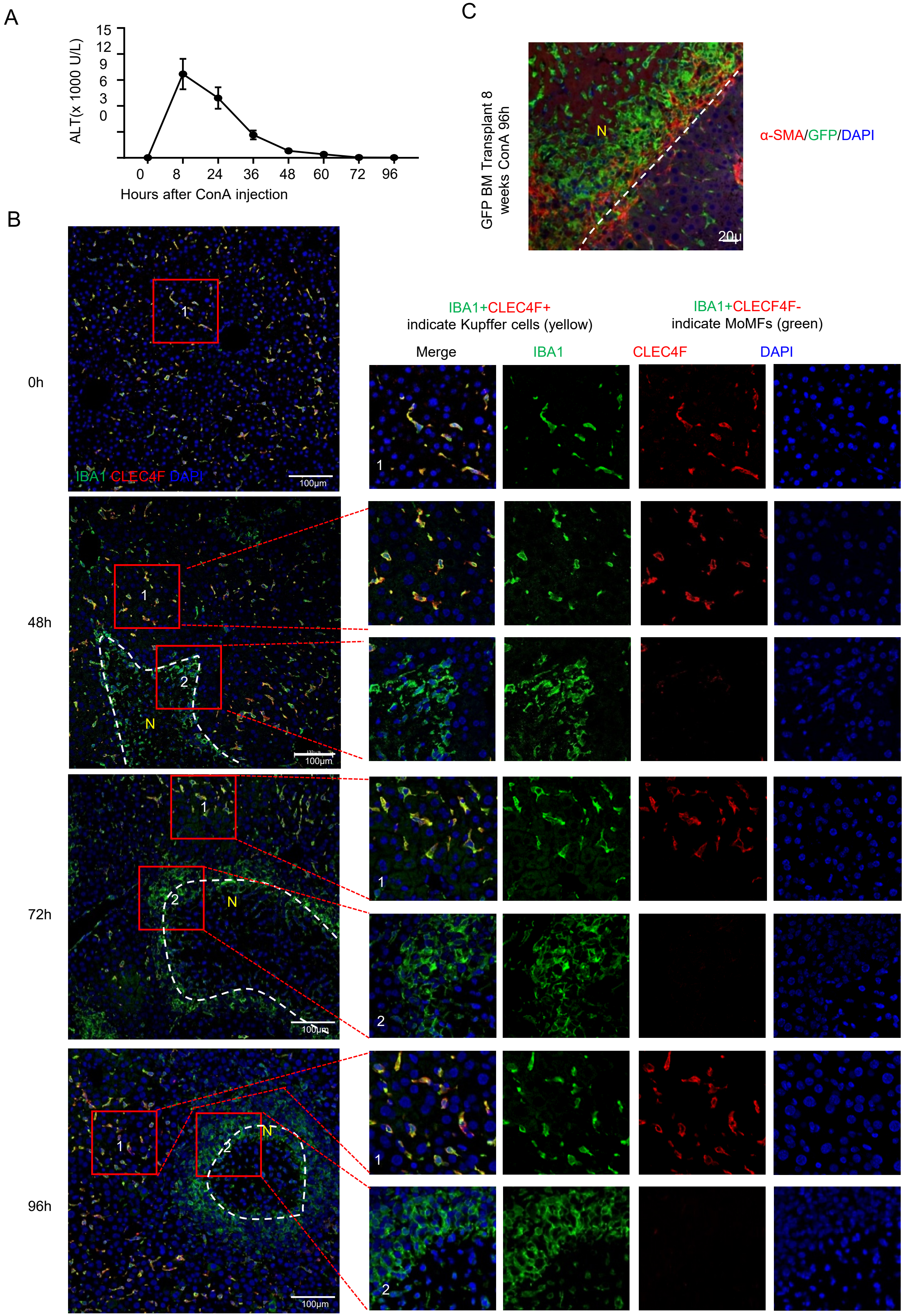

Supplemental Figure 1D-F

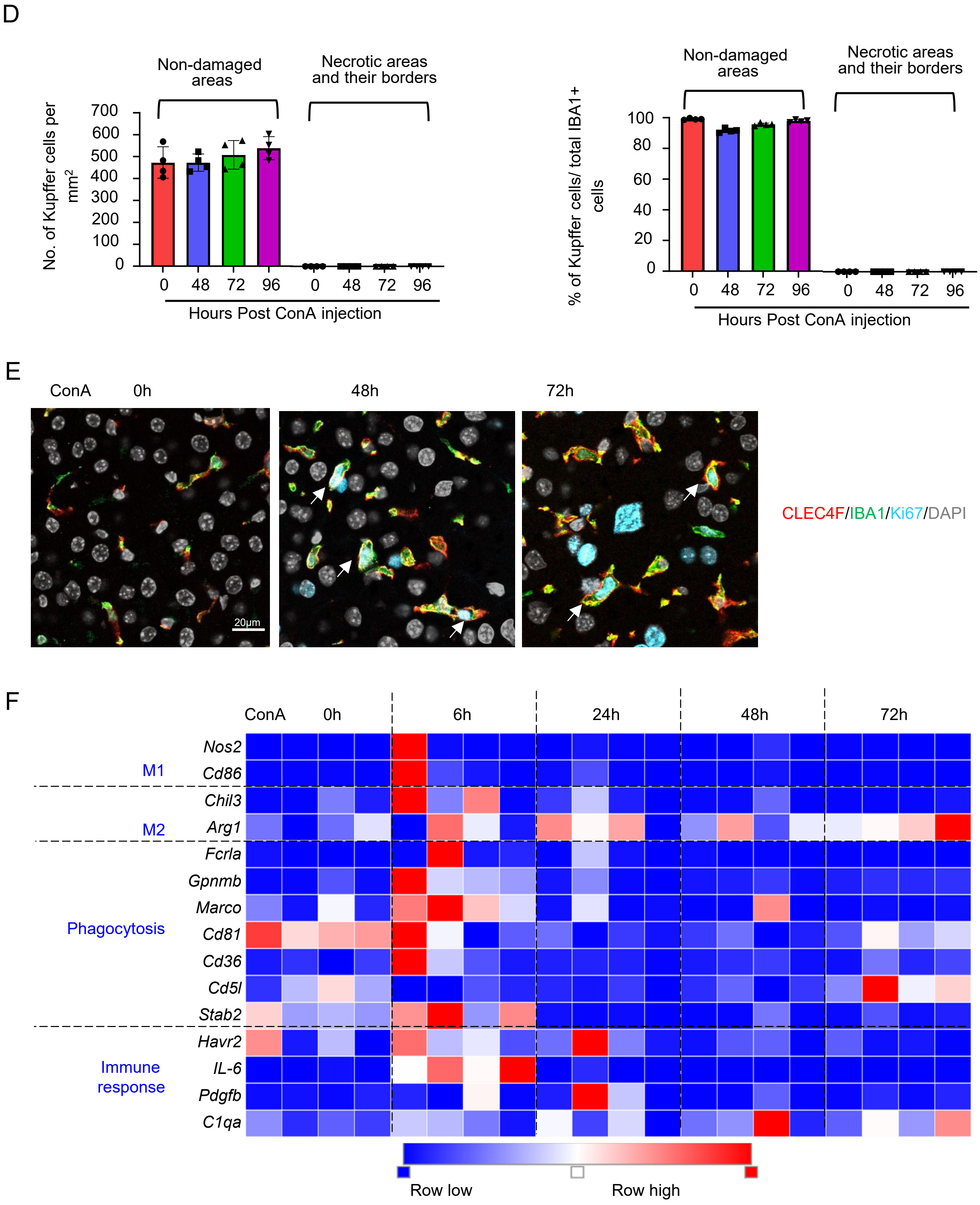

Supplemental Figure.1. **Monocyte-derived macrophages (MoMF) but not Kupffer cells aggregate surrounding necrotic areas.** (A) C57BL/6 mice were treated with 12 mg/kg ConA, serum were collected, and serum ALT levels were measured. (B) C57BL/6 mice were treated with ConA for various time points. IBA1 and CLEC4F double staining of liver tissues. Enlarged images (right panels) showed non-injured area (enlarged image 1) and border area (enlarged image 2). Notes: All IBA1+ cells located on the border area as well as inside necrotic area do not express Kupffer cell marker CLEC4F, suggesting these cells are MoMFs, while Kupffer cells in nondamaged areas express both IBA1+CLEC4F+ markers (yellow color). Most HSCs in the ring-like structure are fully activated (α-SMA+) 96 hours after ConA injection, while HSCs in non-necrotic areas are not activated (α-SMA-). Representative images from three to four mice in each group are shown. (C) C57BL/6 mice were transplanted with bone marrow from GFP positive mice. Six weeks later, these mice were treated with ConA for 48 hours. α-SMA and GFP double staining of liver tissue. Dash line indicates the border area of necrotic area. GFP+ cells indicate these cells are bone marrow-derived. Notes: Transplantation of GFP bone marrow experiments revealed a large number of bone marrow-derived GFP+ cells deposited on the border area, further supporting that most immune cell-enriched rings are bone marrow-derived cells. (D) The Kupffer cells in nondamaged areas and necrotic areas from panel B were quantitated. (E) Representative IBA1, CLEC4F and Ki67 triple staining of liver tissues from ConA-treated mice. Arrows indicated $Ki67^{+}$ proliferating $IBA1^{+}CLEC4F^{+}$ Kupffer cells in non-damaged area. (F) Kupffer cells were isolated from ConA-treated mice by using FACS sorter based on surface makers ($CD45^{+}CD11b^{low}$, $F480^{high}$ $CLEC2^{high}$). Heatmap shows mRNA levels of genes related to Kupffer cell phenotype including M1/M2 polarization, phagocytosis and immune response analyzed by quantitative real-time PCR.

"N" in the images indicates necrotic area. Values represent means ± SD (n=4-6).

Supplemental Figure 2A-C

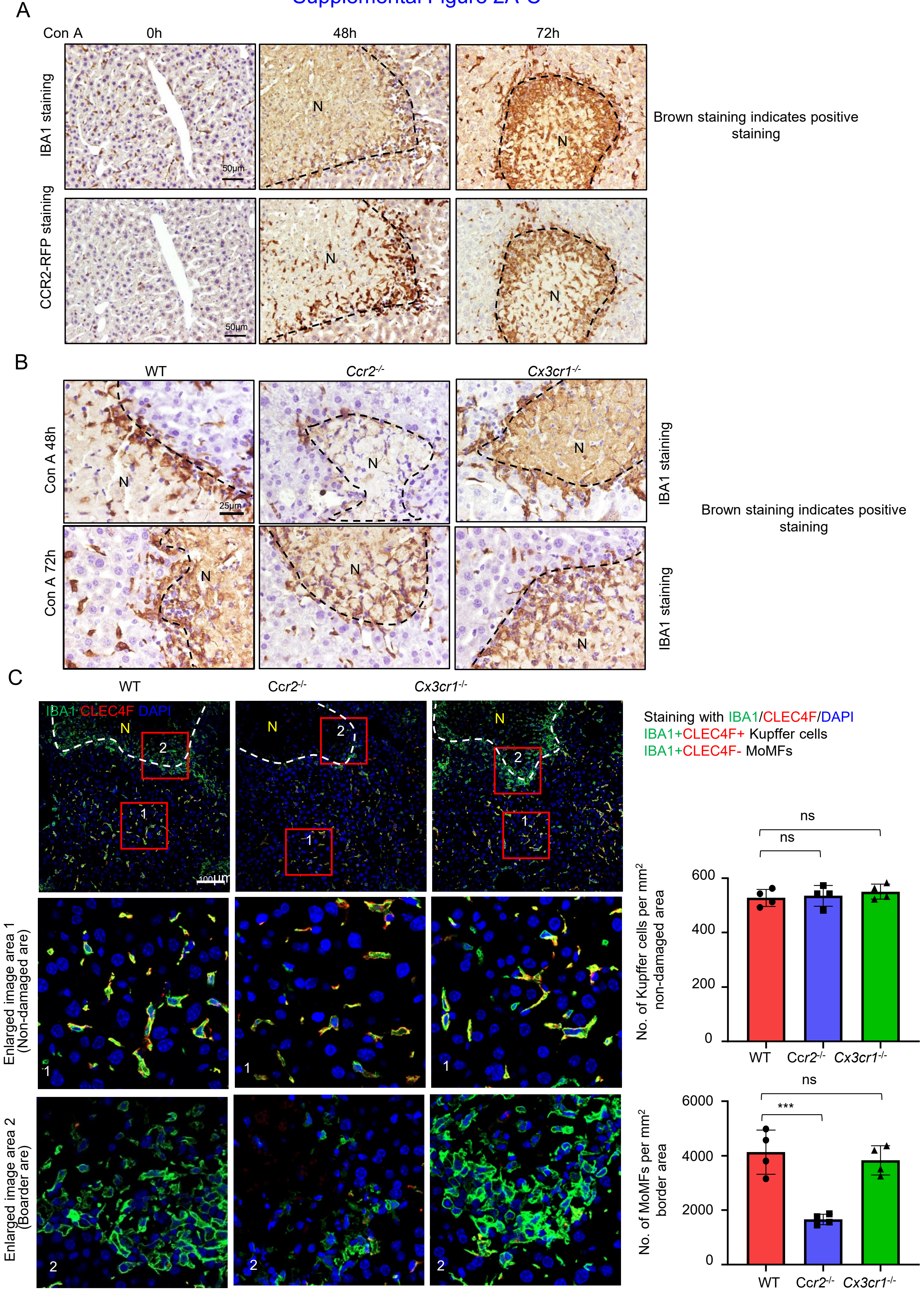

Supplemental Figure 2D-E

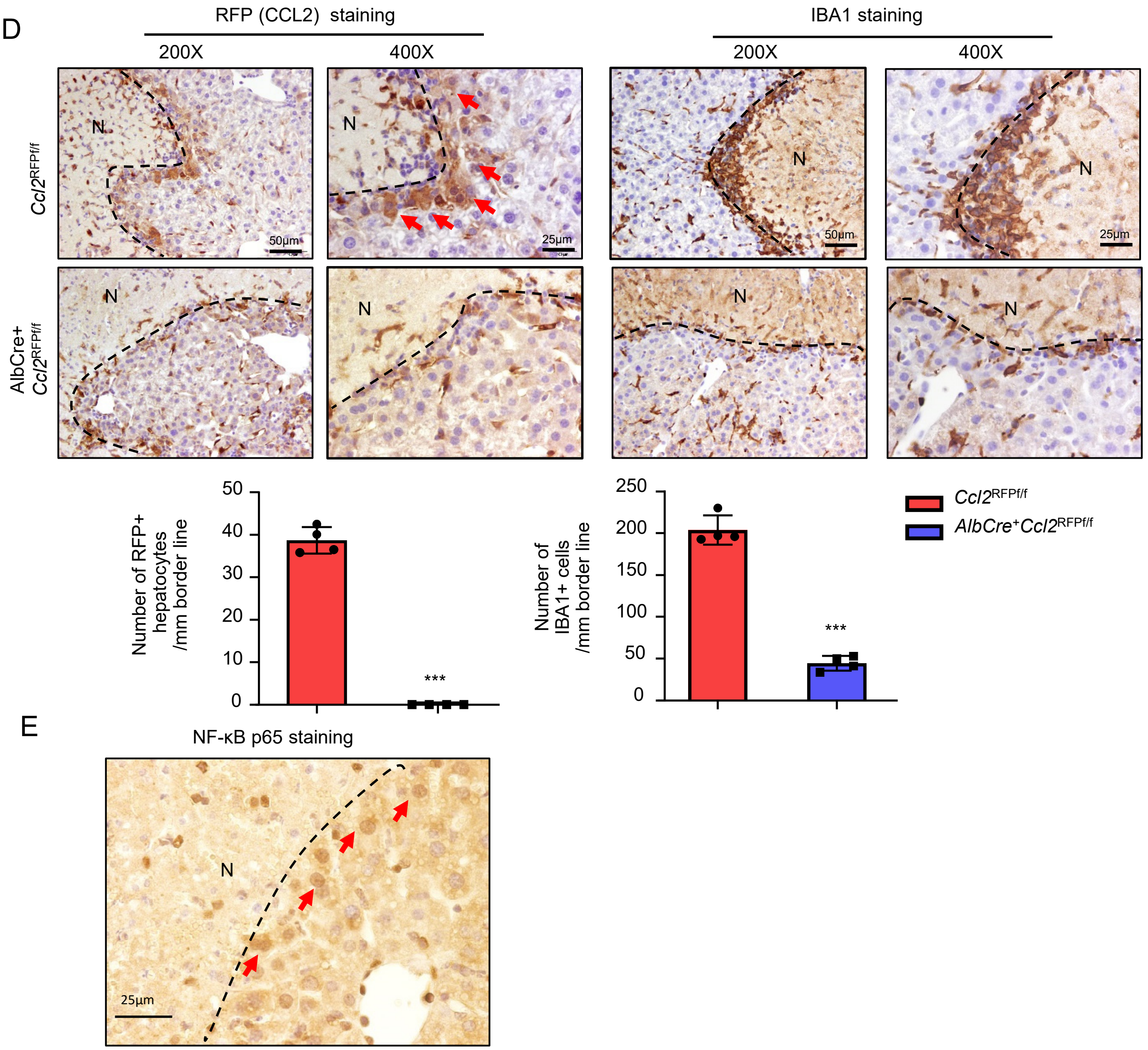

Supplemental Figure 2. **MoMF aggregation surrounding necrotic lesions is dependent on CCR2 and hepatocyte-derived CCL2.** (A) CCR2-RFP mice were treated with ConA for 48 and 72 hours. IBA1 and RFP staining of serial sections of liver tissues. RFP positive staining represents CCR2 expression. Dash line indicates the borderline of necrotic area. Magnification 200X. (B-C) WT, *Ccr2*$^{-/-}$ and *Cx3cr1*$^{-/-}$ mice were treated with ConA for 48 and 72 hours. Liver tissues were subjected to immunostaining with an IBA antibody (panel B), and immunofluorescent staining with IBA1 and CLECF4 antibodies (panel C). Representative images from four mice per group and quantitation are shown. (D) *Ccl2*$^{RFPf/f}$ and *AlbCre*$^{+}$*Ccl2*$^{RFPf/f}$ mice were treated with ConA for 48 hours. RFP and IBA1 staining of liver tissues. RFP positive staining represents CCL2 expression. The numbers of necrotic area RFP+ hepatocytes and IBA+ cells were quantified. Dash line indicates the border area of necrotic area, arrows indicate RFP+ hepatocytes according to morphology. RFP positive staining indicates CCL2 expression. *AlbCre*$^{+}$*Ccl2*$^{RFPf/f}$ mice had *Ccl2* gene deletion in hepatocytes. (E) C57BL/6 mice were treated with ConA for 24 hours. NF-κB p65 staining of liver tissue. Red arrows indicate NF-κB p65 positive nuclei. Magnification 200X and 400X. Representative images from 4 mice in each group are shown. Brown staining indicates positive staining. "N" in the images indicates necrotic area. Values represent means ± SD, n=4. Statistical significance was assessed using 2-tailed Student's t test for comparing 2 groups (D) and 1-way ANOVA followed by Tukey's post hoc test for multiple groups (C). ***P<0.001

Supplemental Figure 2.

Notes:

(**A**) By using heterozygous CCR2^{RFP} reporter mice, in which CCR2+ monocytes were labelled with RFP, we found most of IBA1^{+} cells surround the necrotic areas expressed RFP, supporting that the "ring-like" structure around the necrotic areas were formed by invading IBA1^{+}CCR2^{+} MoMFs. (**B-C**) we examined whether CCR2 and CX3CR1, two critical chemokine receptors for monocyte migration, contribute MoMF infiltration, we used *Ccr2*$^{-/-}$ and *Cx3cr1*$^{-/-}$ mice. Our data revealed that the formation of IBA1^{+} CCR2^{+} MoMFs was markedly diminished in *Ccr2*$^{-/-}$ but not in *Cx3cr1*$^{-/-}$ mice, suggesting CCR2 but not CX3CR1 contributes to the formation of "ring-like" structure. (**D**) we explored the source of CCL2, a well-known ligand for CCR2 by using heterozygous CCL2-RFP$^{f/-}$ reporter mice and immunohistochemical staining of RFP expression (representing CCL2 expression). A substantial population of hepatocytes surround necrotic areas strongly expressed CCL2-RFP, and a small population of non-parenchymal cells also expressed CCL2-RFP. In ConA treated hepatocyte specific *Ccl2* KO mice (*AlbCre*$^{+}$*Ccl2*$^{RFPf/f}$), some non-parenchymal cells but no hepatocytes were RFP+ in injured livers, indicating a specific CCL2 deletion in hepatocytes. Compared with *Ccl2*$^{RFPf/f}$ control mice, hepatocyte-specific *Ccl2* KO mice showed a signification reduction of IBA1^{+}CCR2^{+} MoMFs aggregation surrounded the necrotic areas. Representative images from multiple mice are shown. (**E**) In WT mice treated with ConA, the nuclei of hepatocytes close to necrotic area were stained positive with NF-κB p65 antibody, indicating NF-κB activation. NF-κB is a well documented signaling pathway that promotes CCL2 expression.

Supplemental Figure 3A:

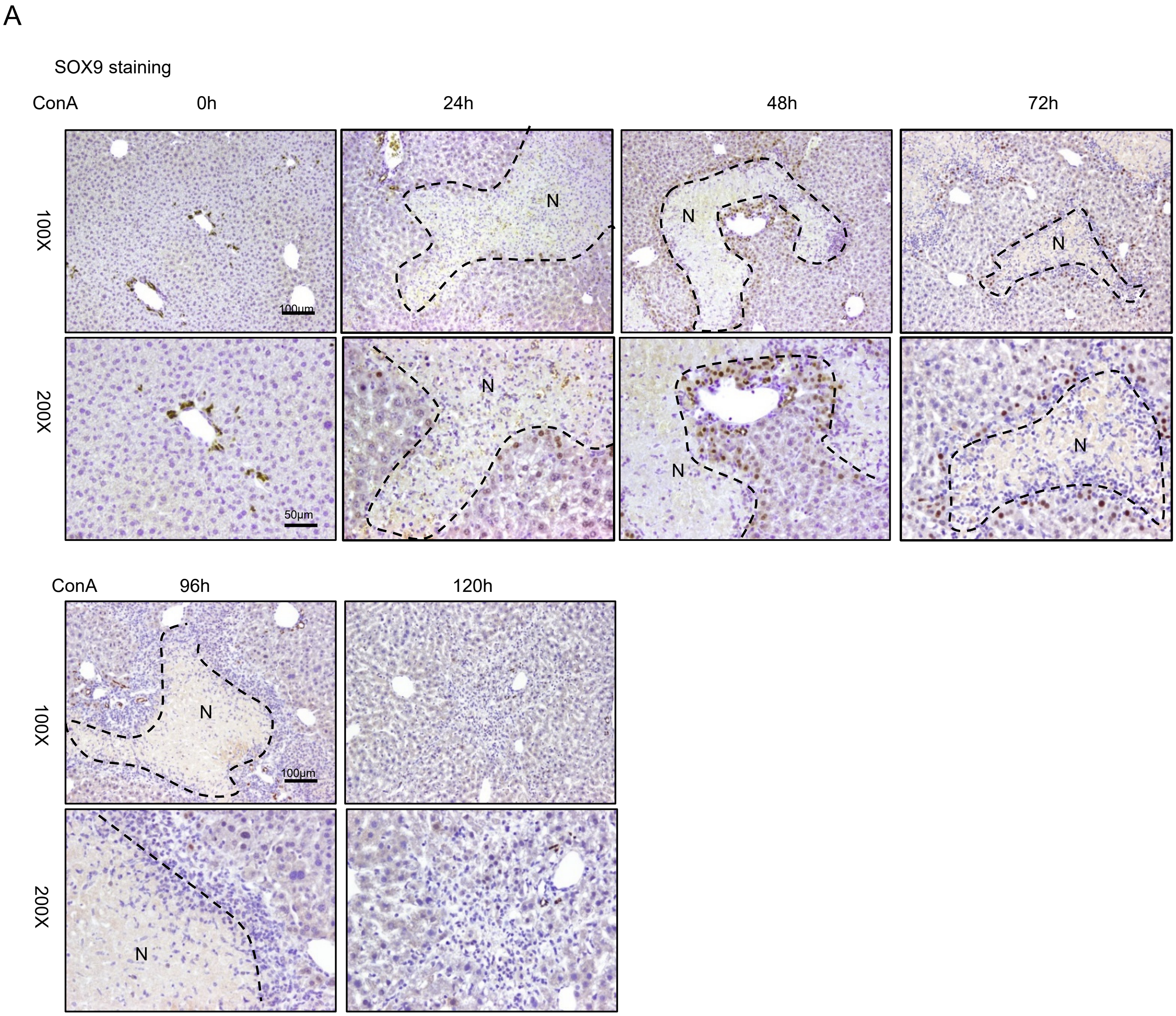

Supplemental Figure 3A. **Kinetics of SOX9⁺ cells in ConA induced liver injury.** (A) C57BL/6 mice were treated with ConA. SOX9 staining of liver tissues from the mice treated with ConA for various time points. Magnification 100X and 200X . Representative images from 3-4 mice in each group are shown.

Supplemental Figure 3B-F:

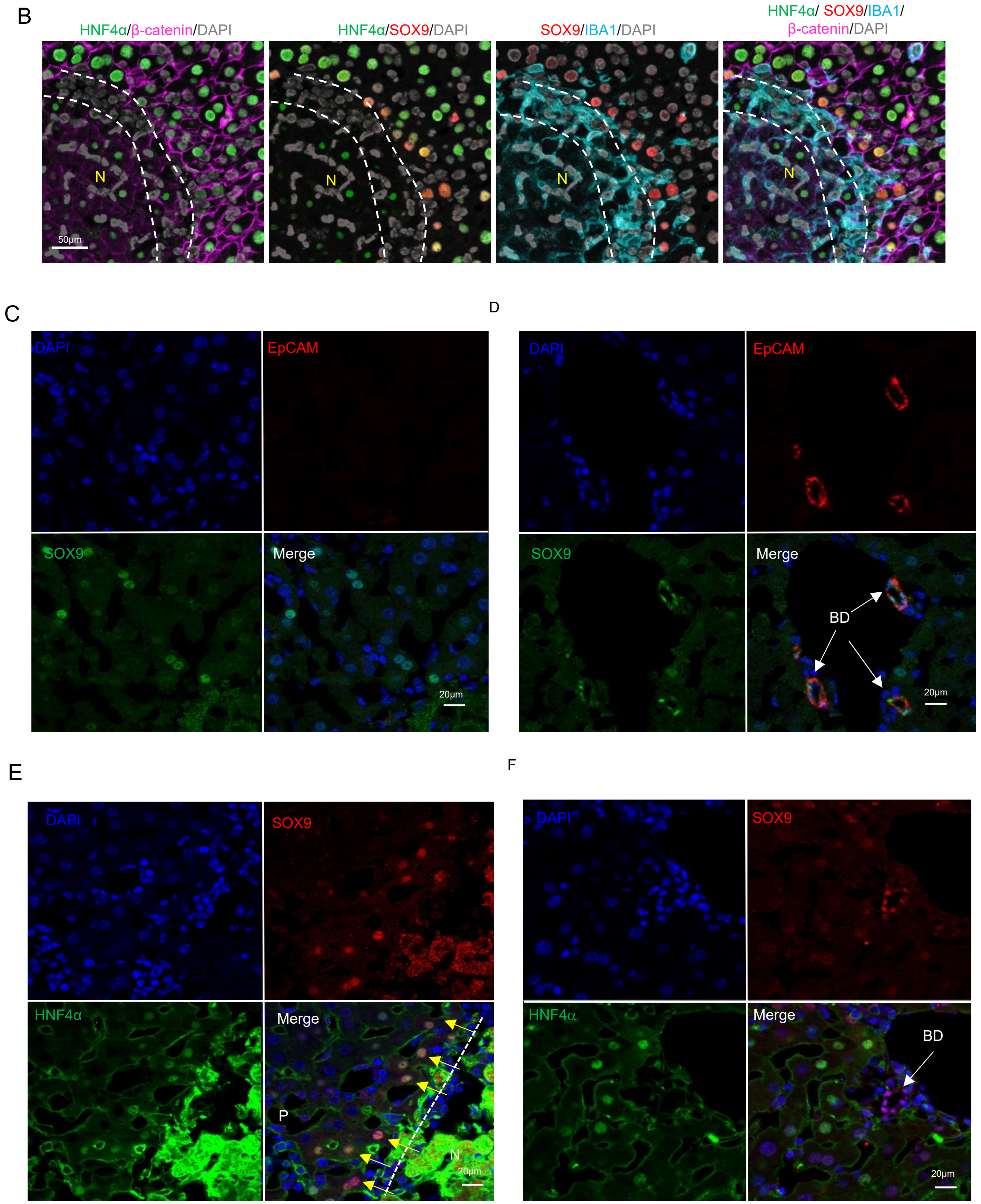

Supplemental Figure 3B-F. **SOX9$^+$ cells surrounding necrotic areas express hepatocyte marker but not bile duct cell/liver progenitor cell markers.** C57BL/6 mice were treated with ConA for 48 hours. Liver tissues were subjected to following staining. (**B**) Representative multiplex immunofluorescent staining of liver tissues with several antibodies. (**C**): SOX9 and EpCAM double staining on necrotic area. (**D**): SOX9 and EpCAM double staining on portal vein area. Arrows indicate bile duct. (**E**) SOX9 and HNF4α double staining on necrotic area. Yellow arrows indicate SOX9/HNF4α double positive hepatocytes. (**F**) SOX9 and HNF4α double staining on portal vein area. White arrow indicates bile duct. Dash line indicates the border area of necrotic area, Magnification 400X. Representative images from 4 mice in each group are shown.

Notes: To determine whether the smaller hepatocytes around necrotic areas are liver progenitor cells (LPCs) or bile duct cells (BDCs), we performed immunostaining of SOX9 (LPC/BDC marker), EpCAM (LPC/BDC marker), and HNF4α (mature hepatocyte marker). SOX9 expression was only detected in BDCs from normal livers (supporting S3A), while SOX9 expression was detected in both BDCs and small hepatocytes mainly surrounded necrotic areas from injured livers (Supplemental Figure 3A). The morphology of these SOX9$^+$ hepatocyte-like cells was obviously different from bile duct cells with round nuclear and no lumen while SOX9$^+$ bile duct cells form tube-like structures with lumen and have oval shape nuclear (Supplemental Figure 3B). These SOX9$^+$ hepatocyte-like cells expressed hepatocyte marker HNF4α but not LPC/BDC marker EpCAM; whereas SOX9$^+$ BDCs expressed EpCAM but not HNF4α (Supplemental Figure 3C-F), suggesting SOX9$^+$ hepatocyte-like cells more resembled hepatocytes than LPCs/BDCs.

Supplemental Figure 4

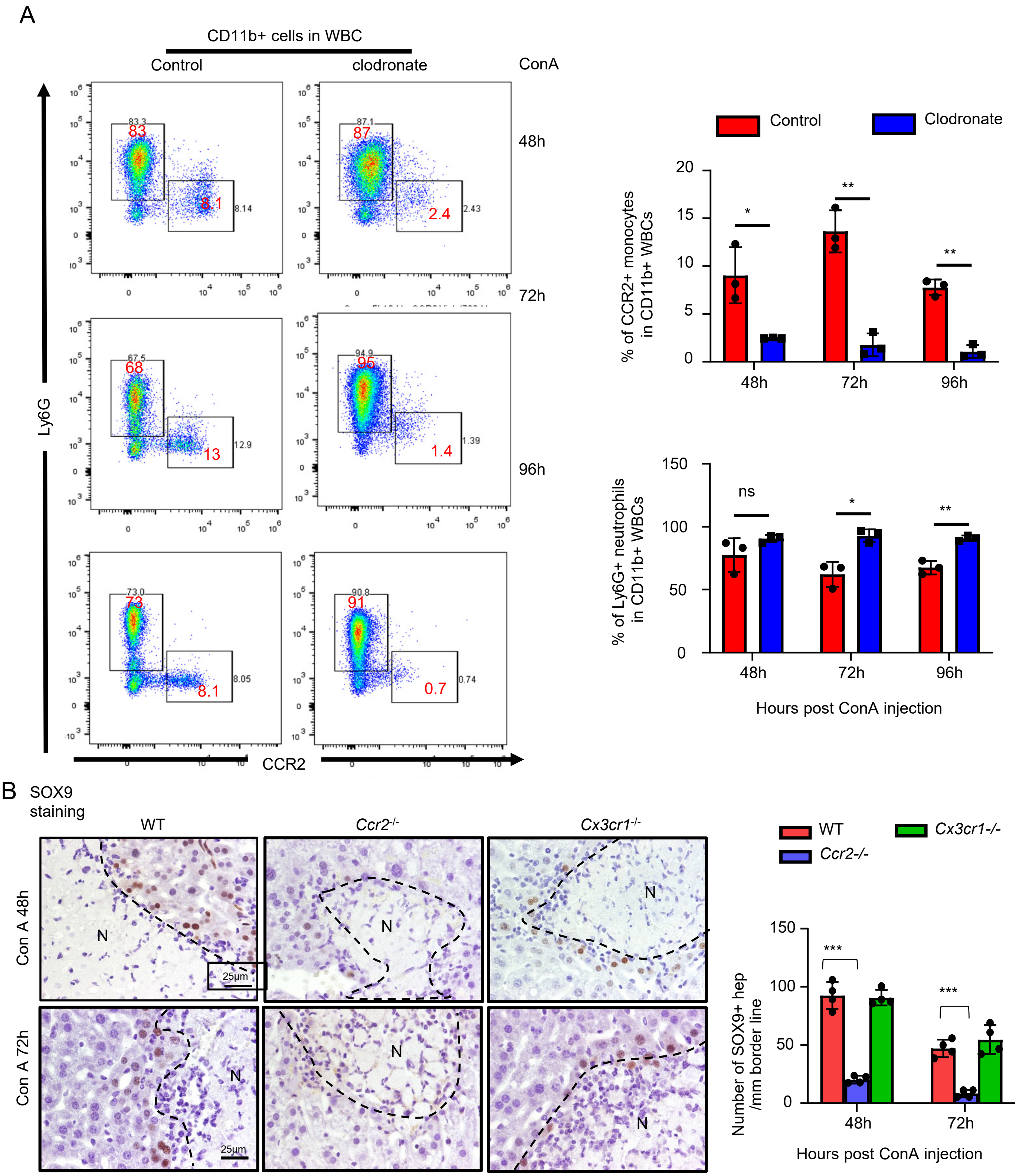

Supplemental Figure 4. **Contribution of macrophages to the induction of SOX9$^+$ hepatocytes in ConA induced liver injury.** (A) C57BL/6 mice were treated with ConA for 24 hours, then injected with clodronate liposome or control liposome every 24 hours for three injections. WBCs were collected and subjected to flow cytometry analyses of monocytes and neutrophils. The percentages of CCR2+ monocytes and Ly6G+ neutrophils were counted. (B) SOX9 staining of liver tissues from WT, *Ccr2*$^{-/-}$ and *Cx3cr1*$^{-/-}$ mice treated with ConA for 48 hours. Representative images are shown. The number of necrotic border area SOX9$^+$ hepatocytes in (B) were quantified. “N” indicates necrotic areas. Values represent means ± SD, n=3-4. Statistical significance was assessed using 2-tailed Student’s t test for comparing 2 groups (A) and 1-way ANOVA followed by Tukey’s post hoc test for multiple groups (B). ***P<0.001. ns: not significant

Supplemental Figure 5:

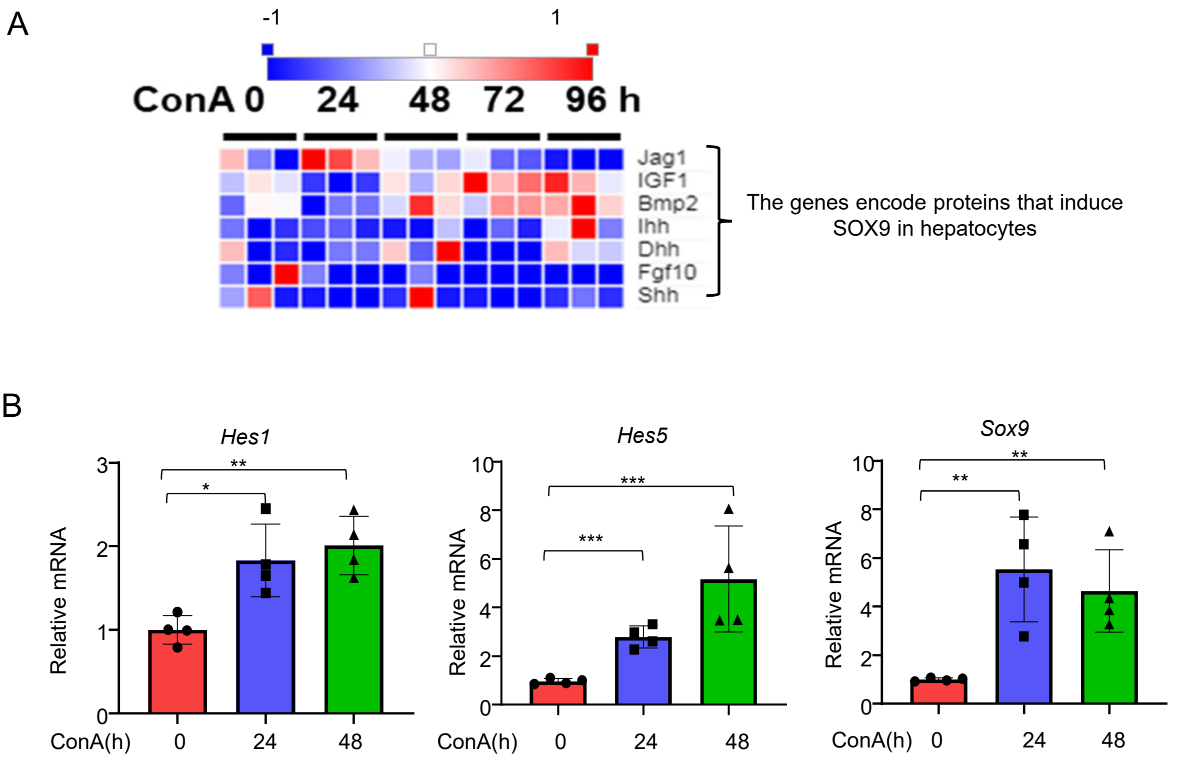

Supplemental Figure 5. **mRNA expression of *Jagged1* and *Notch* downstream genes in the liver with ConA injection.** (A) C57BL/6 mice were treated with ConA for 24~96 hours. mRNA were isolated from CD45+CD11b+CCR2+Ly6G^{-} MoMFs purified from the liver. The expression levels of known *Sox9* upstream genes (n=3). (B) mRNAs were extracted from the liver of mice treated with ConA for 24 and 48 hours. The expression levels of *Hes1, Hes5* and *Sox9* were measured by RT-qPCR (n=3-4).

Values represent means ± SD. Statistical significance was assessed 1-way ANOVA followed by Tukey's post hoc test for multiple groups (B). .**P*<0.05, ***P*<0.01, ****P*<0.001.

Supplemental Figure 6:

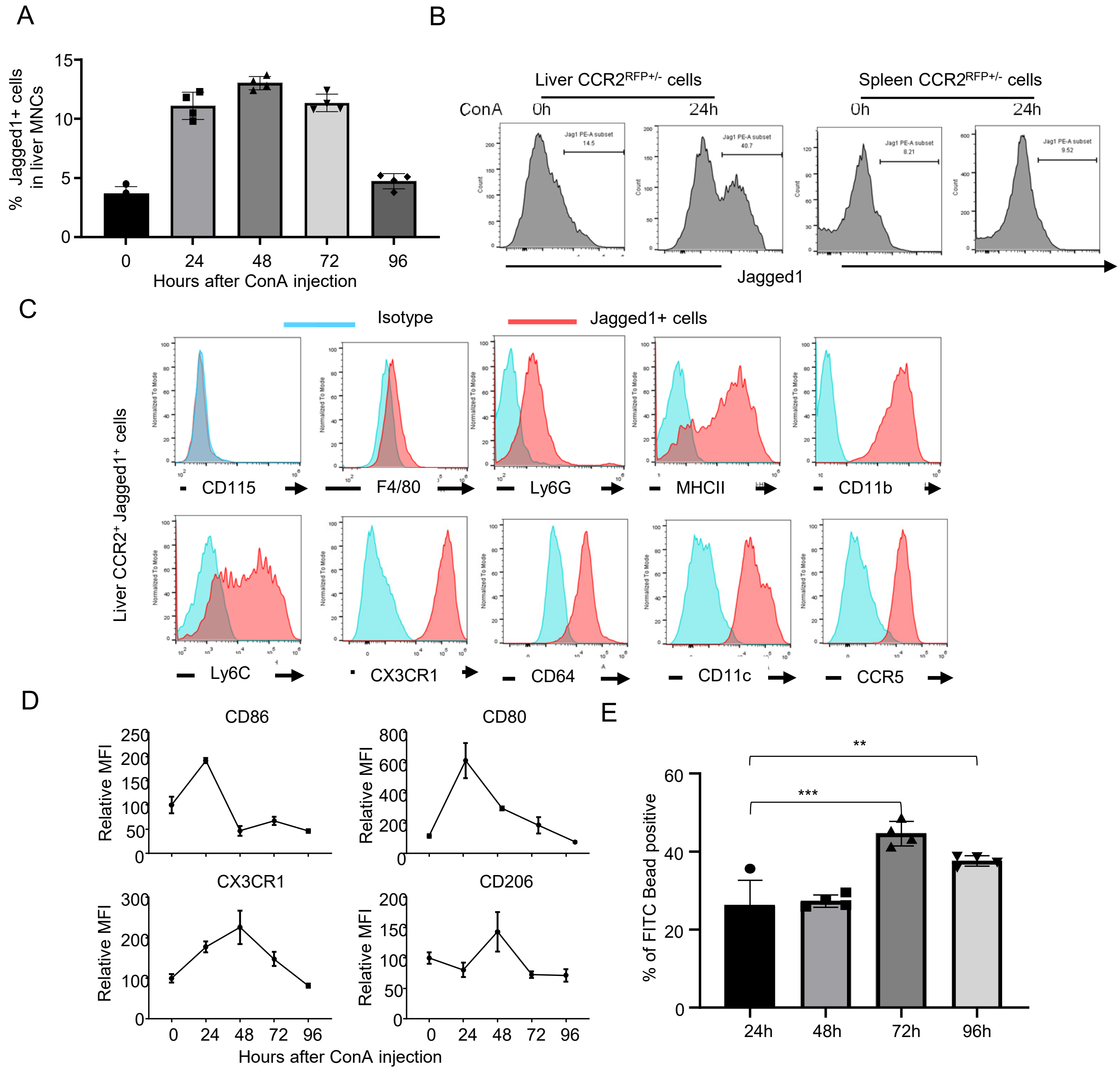

Supplemental Figure 6. **Characterization of JAG1 expressing monocytes in the liver after ConA injection.** (A) C57BL/6 mice were treated with ConA for 24-96 hours. Liver MNCs were isolated from liver 24-96 hours after ConA injection, the percentage of JAG1+ cells in liver and spleen MNCs were determined by flow cytometry analysis (n=4). (B) *Ccr2*$^{RFP+/-}$ mice were treated with ConA for 48 hours. Spleen MNCs and Liver MNCs were isolated, JAG1 expression was measured in RFP+ cells by flow cytometry (n=5). (C) Expressions of surface markers on liver CCR2 (RFP)+Jagged1+ cells were determined by flow cytometry (n=5). (D) M1 and M2 surface marker expressions (Mean fluorescence intensity, MFI) on CCR2+ liver MNCs were analyzed in different time points after ConA injection, n=4. (E) The phagocytic ability of CCR2+ liver MNCs were analyzed by FITC beads uptake assay in different time points after ConA injection (n=4). Values represent means ± SD. Statistical significance was assessed using 1-way ANOVA followed by Tukey's post hoc test for multiple groups (E). ***P<0.001. JAG1 was not detected in T, B, NK, neutrophils before or after ConA injection (data not shown).

Supplemental Figure 7:

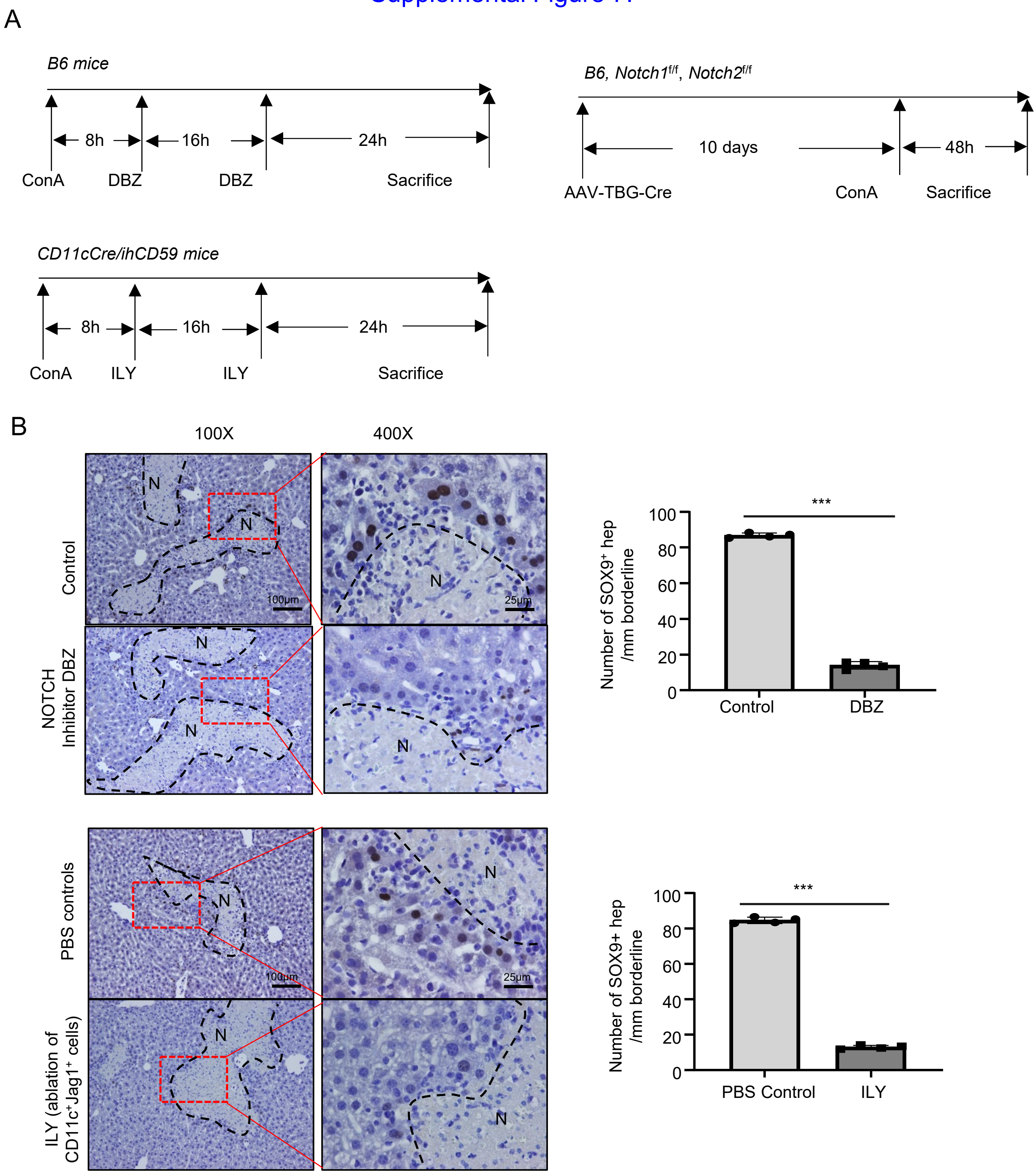

Supplemental Figure 7. **Blocking Notch signaling or ablation of Jagged1⁺ monocytes in mice.** (A) Schematic diagram for ConA and DBZ treatment for WT mice. Schematic diagram for ConA and ILY treatment for *CD11cCre/ihCD59* mice. Schematic diagram for AAV-TBG-Cre and ConA treatment for WT, Notch1$^{f/f}$ and Notch2$^{f/f}$ mice. (B) Mice were treated as in (A). Liver tissues were subjected to SOX9 staining. Representative images from four to five mice in each group are shown. The number of SOX9+ hepatocytes was quantified. Values represent means ± SD, n=4. Statistical significance was assessed using 2-tailed Student's t test for comparing 2 groups (B). ****P*<0.001.

ihCD59 mice were injected with ILY to deplete MoMFs as described previously (Feng et al. *J Clin Invest.* 2016;126:2321-33). Injection of ILY depleted Jagged1+MoMFs in *CD11cCre/ihCD59* mice as confirmed by flow cytometry (data not shown).

Supplemental Figure 8:

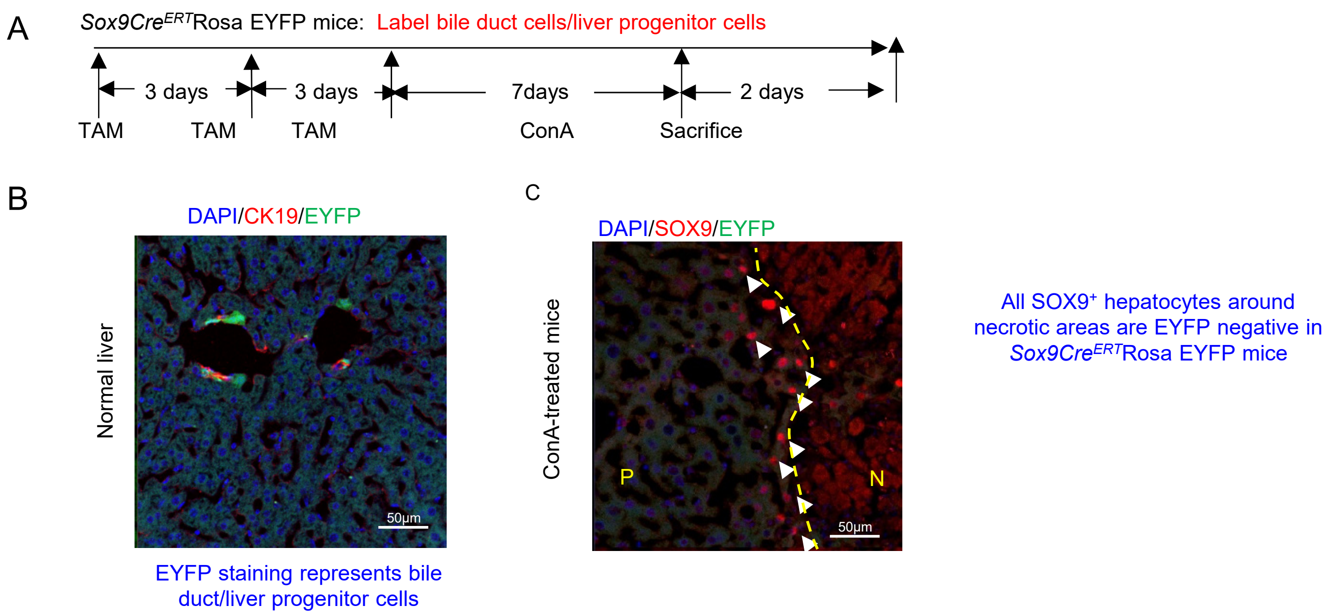

Supplemental Figure 8. **Efficient and specific labelling of bile duct/liver progenitor cells for fate tracing.** (A). Schematic diagram for fate tracing assay. Sox9CreERT/Rosa-EYFP mice received 3 tamoxifen treatments to label bile duct cells and liver progenitor cells. (B) Livers from these mice without ConA treatment were collected for staining with CK19, a marker for bile duct cells/liver progenitor cells. (C) These mice were treated with ConA for 48 hours. SOX9 and EYFP double staining of the liver tissues from panel A. Arrows indicate $SOX9^{+}$ hepatocytes. Representative images from 5 mice in each group are shown.

Supplemental Figure 9:

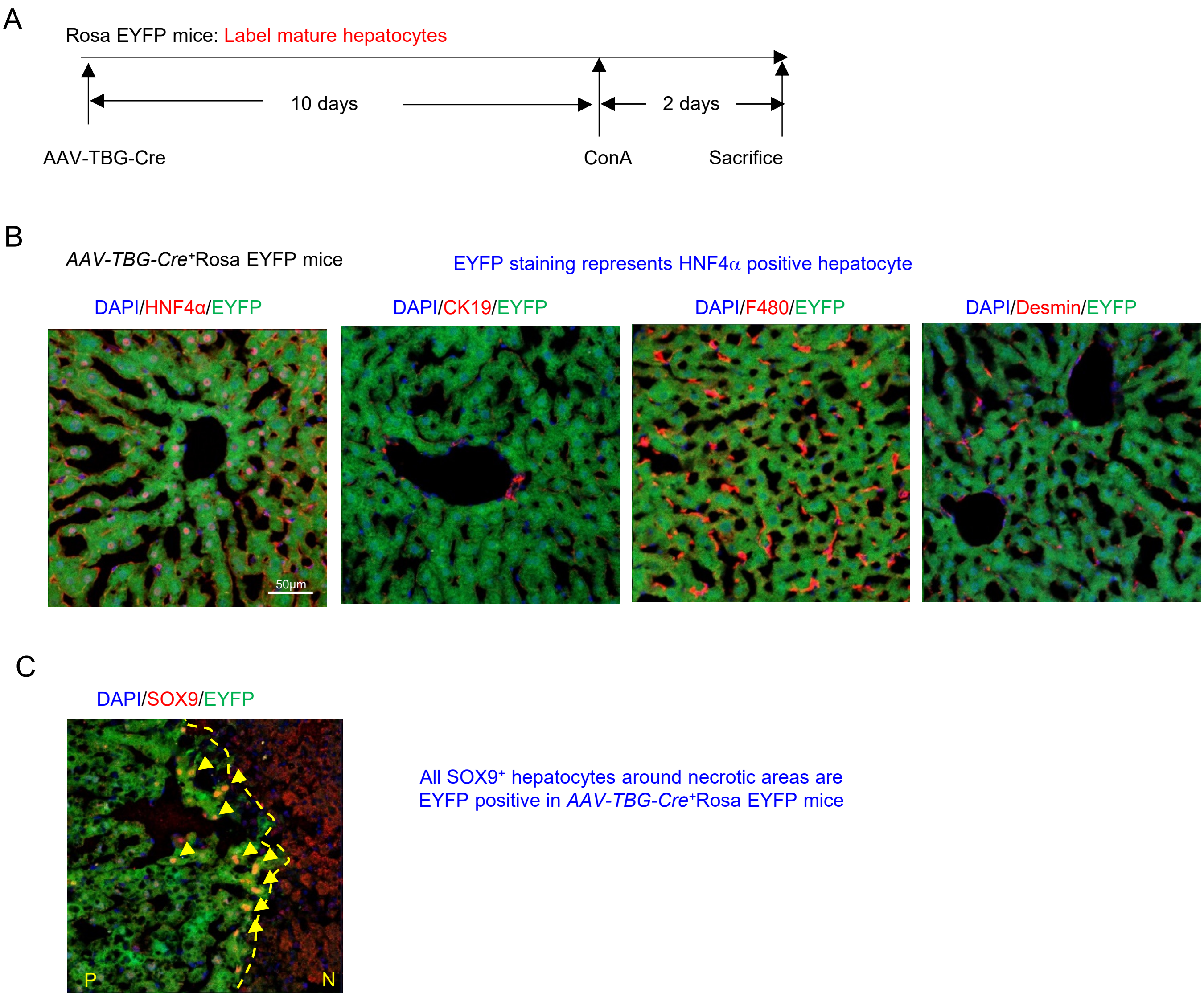

Supplemental Figure 9. **Efficient and specific labelling of mature hepatocytes for fate tracing.** (A) Rosa-EYFP mice received AAV-TBG-Cre treatment for 10 days to label hepatocytes. (B) Livers from normal mice with ConA treatment were collected for staining with HNF4α, CK19, F4/80 and desmin. (C) These mice were treated with ConA for 48 hours. SOX9 and EYFP double staining of the liver tissues from panel A. Arrows indicate $SOX9^{+}$ hepatocytes. Representative images from 5 mice in each group are shown.

Notes: $EYFP^{+}SOX9^{+}$ hepatocytes were detected in *AAV*-TBG-Cre^{+}Rosa-EYFP reporter mice (matured hepatocytes were labelled) but not in *Sox9*-Cre^{ERT}Rosa26-EYFP reporter mice (LPCs/BDCs were labelled), suggesting $SOX9^{+}$ hepatocytes were derived from mature hepatocytes.

Supplemental Figure 10:

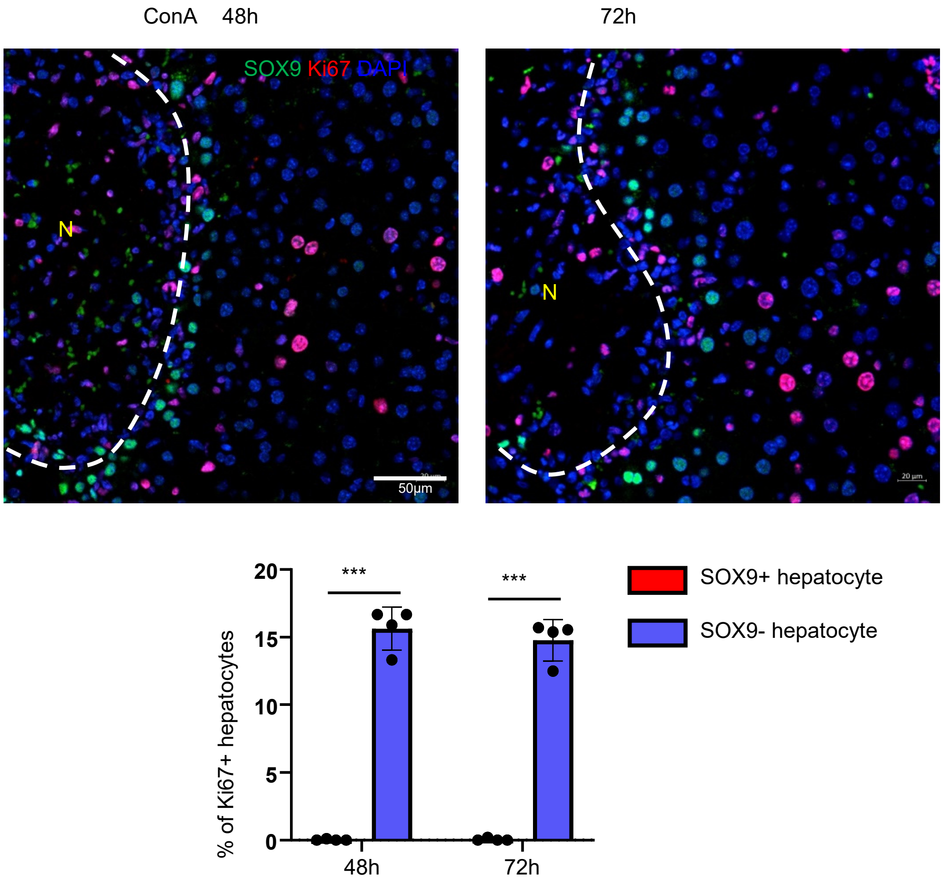

Supplemental Figure 10. **SOX9⁻ hepatocytes but not SOX9⁺ hepatocytes proliferate during the recovery stage of ConA-induced liver injury.** C57BL/6 mice were treated with ConA for 48 and 72 hours. Representative imaging of Ki67 and SOX9 double staining of liver tissues from 4 mice per group. The percentage of Ki67+ hepatocytes was quantified.

"N" indicates necrotic area. Values represent means ± SD n=4. Statistical significance was assessed using 2-tailed Student's t test for comparing 2 groups. ***P<0.001.

Supplemental Figure 11:

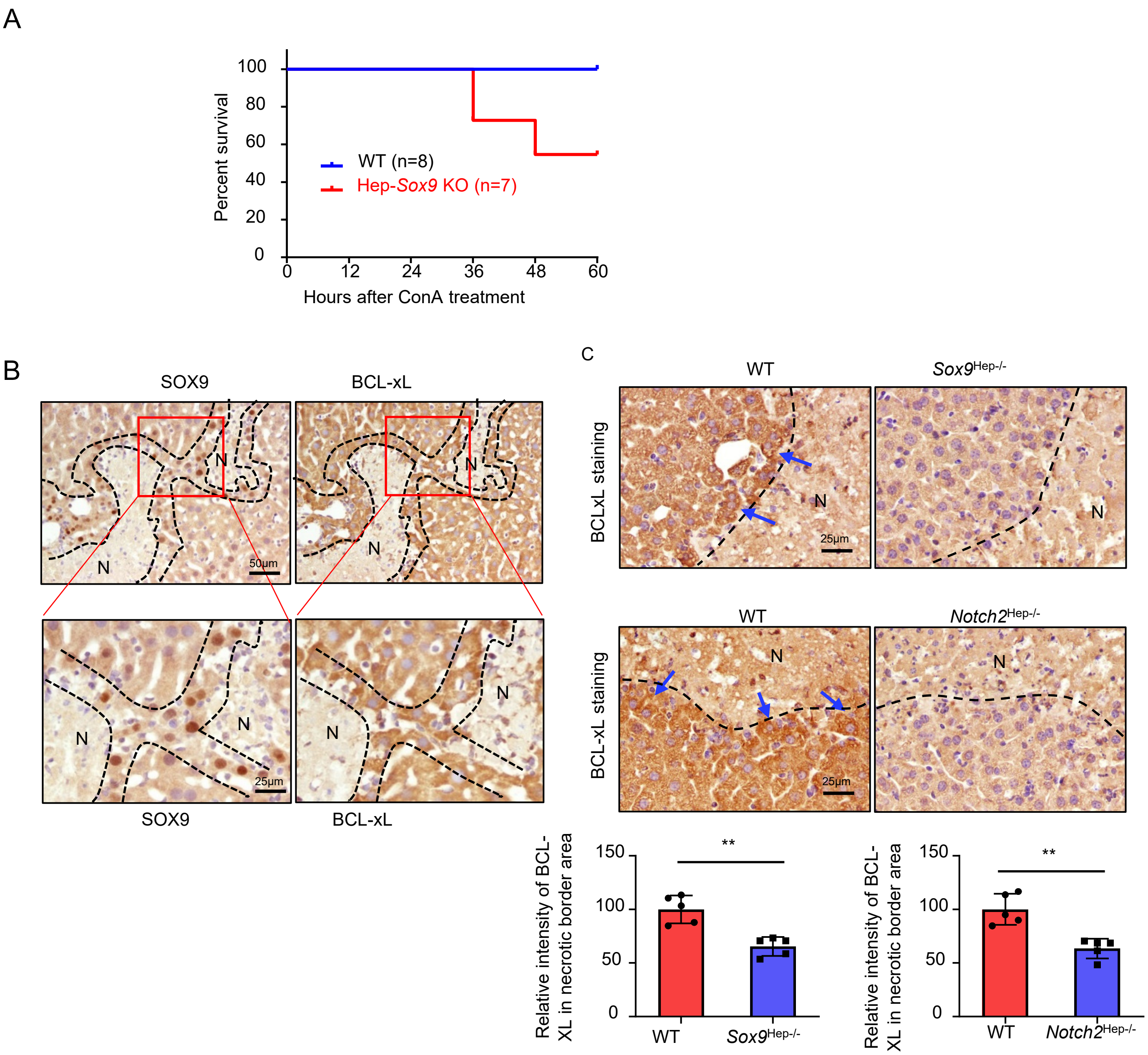

Supplemental Figure 11. **Hepatocyte-specific *Sox9* KO mice are more sensitive to liver injury and have impaired liver regeneration.** WT and *Sox9*$^{Hep-/-}$ mice were treated with ConA for different time points, BrdU (10mg/kg) was given 2 hours before sacrifice. (A) The survival rate until 60 hours was measured (n=7-8). (B) C57BL/6 mice were treated with ConA for 48 hours. SOX9 and BCL-xL staining of serial sections of liver tissues. (C) WT, *Sox9*$^{Hep-/-}$ and *Notch2* $^{Hep-/-}$ mice were treated with ConA for 48 hours. BCL-xL was stained for the liver tissues. Brown staining indicates positive staining. Representative images from 5 mice in each group are shown. The intensity of necrotic border area BCL-XL staining in (C) were quantified.

"N" indicates necrotic area. Values represent means ± SD, n=5. Statistical significance was assessed using 2-tailed Student's t test for comparing 2 groups (C). $**P<0.01$, $***P<0.001$.

Supplemental Figure 12:

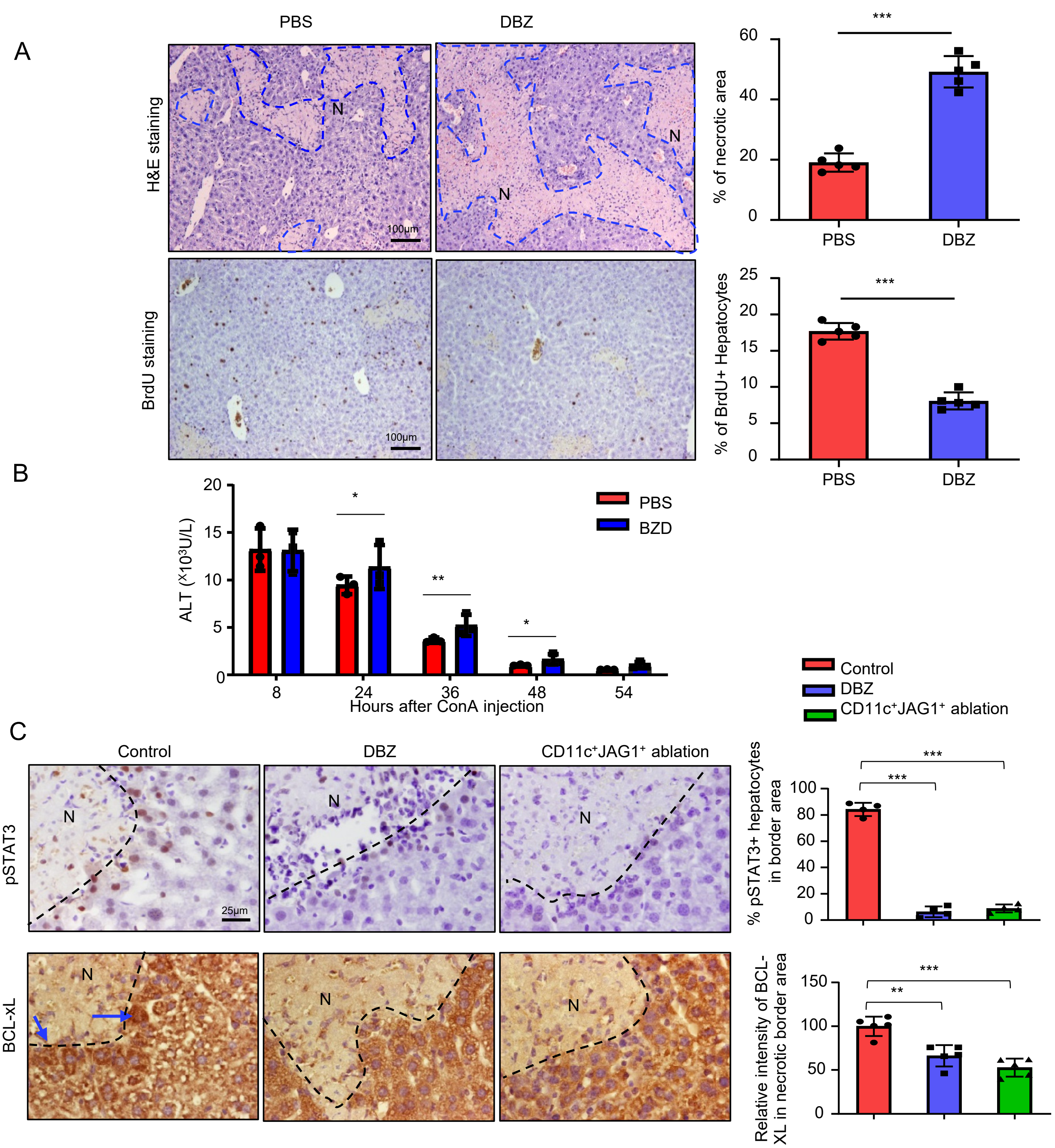

Supplemental Figure 12. **Notch inhibition delays liver repair.** (A, B) B6 mice were treated with ConA for different time points with or without Notch signaling inhibitor DBZ (8, 24 and 48 hours after ConA treatment), BrdU was given 2 hours before sacrifice. (A) Representative H&E staining and BrdU staining of liver tissues from mice 72 hours after ConA injection. The percentage of necrotic area and $BrdU^+$ hepatocytes were quantified. (B) Serum ALT levels. (C) B6 mice were treated with ConA with or without DBZ as Fig.S7A. CD11cCre/ihCD59 mice were treated with ConA and ILY as Fig. S7B to deplete CD11c+ cells. Representative images of pSTAT3 and Bcl-xL staining on liver tissues 48 hours after ConA injection from five mice in each group. The percentage of pSTAT3+ hepatocytes and the intensity of BCL-XL staining in necrotic border area were quantified. "N" indicates necrotic area. Values represent means ± SD. n=3-5. Statistical significance was assessed using 2-tailed Student's t test for comparing 2 groups (A and B) and 1-way ANOVA followed by Tukey's post hoc test for multiple groups (C). **P*<0.05, ***P*<0.01, ****P*<0.001.

Supplemental Figure 13:

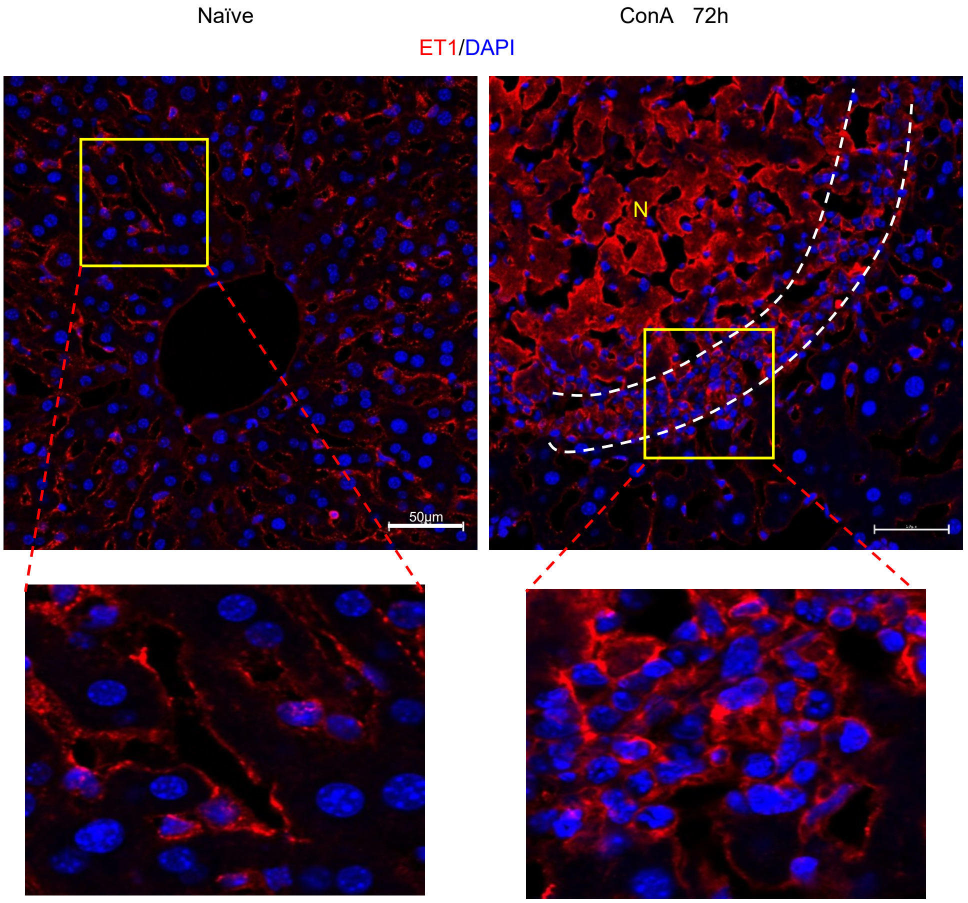

Supplemental Figure 13. **ET1 expression in border area.** C57BL/6 mice were treated with ConA for 72 hours. ET1 staining on liver tissues of naïve mice or ConA treated mice. Dash line indicates the border area of necrotic area. "N" indicates necrotic area. Representative images from 3 mice in each group are shown.

Notes: In naïve mice, ET1 staining showed an LSEC pattern which was in agreement with previous studies that ET1 is derived from LSECs. In ConA treated mice, in addition to LSECs, ET1 staining was also found in the border area (most likely macrophages). In addition, the strong ET1 staining inside necrotic area is probably due to non-specific staining of necrotic tissues.

Supplemental Figure 14:

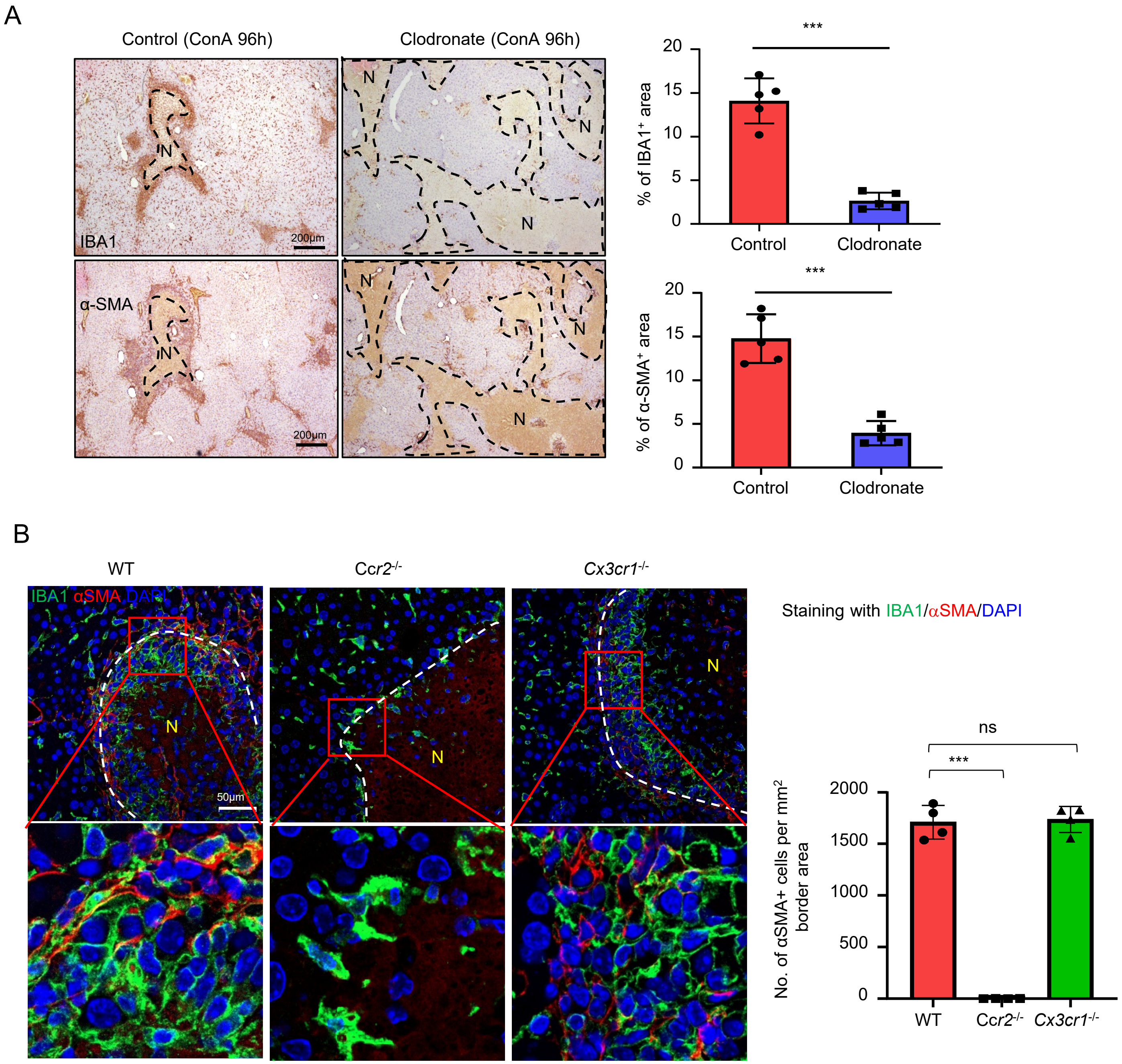

Supplemental Figure 14. **MoMFs support the activation of HSCs after ConA induced liver injury.** (A) C57BL/6 mice were treated with ConA for 96 hours. Clodronate liposome or control liposome were given to these mice 48 and 72 hours after ConA injections. IBA1 and α-SMA staining of serial sections of liver tissues. Representative images from 5 mice in each group are shown. The percentage of IBA1+ and α-SMA+ area were quantified. . "N" indicates necrotic area. Notes: In clodronate-treated group, both IBA and α-SMA staining were abolished. (B) WT, *Ccr2*$^{-/-}$ and *Cx3cr1*$^{-/-}$ mice were treated with ConA for 48 and 72 hours. Liver tissues were subjected to immunofluorescent staining with IBA1 and αSMA antibodies. Representative images from 4 mice per group and quantitation are shown. Values represent means ± SD, n=4-5. Statistical significance was assessed using 2-tailed Student's t test for comparing 2 groups (A) and 1-way ANOVA followed by Tukey's post hoc test for multiple groups (B). ***P<0.001.

Supplemental Figure 15

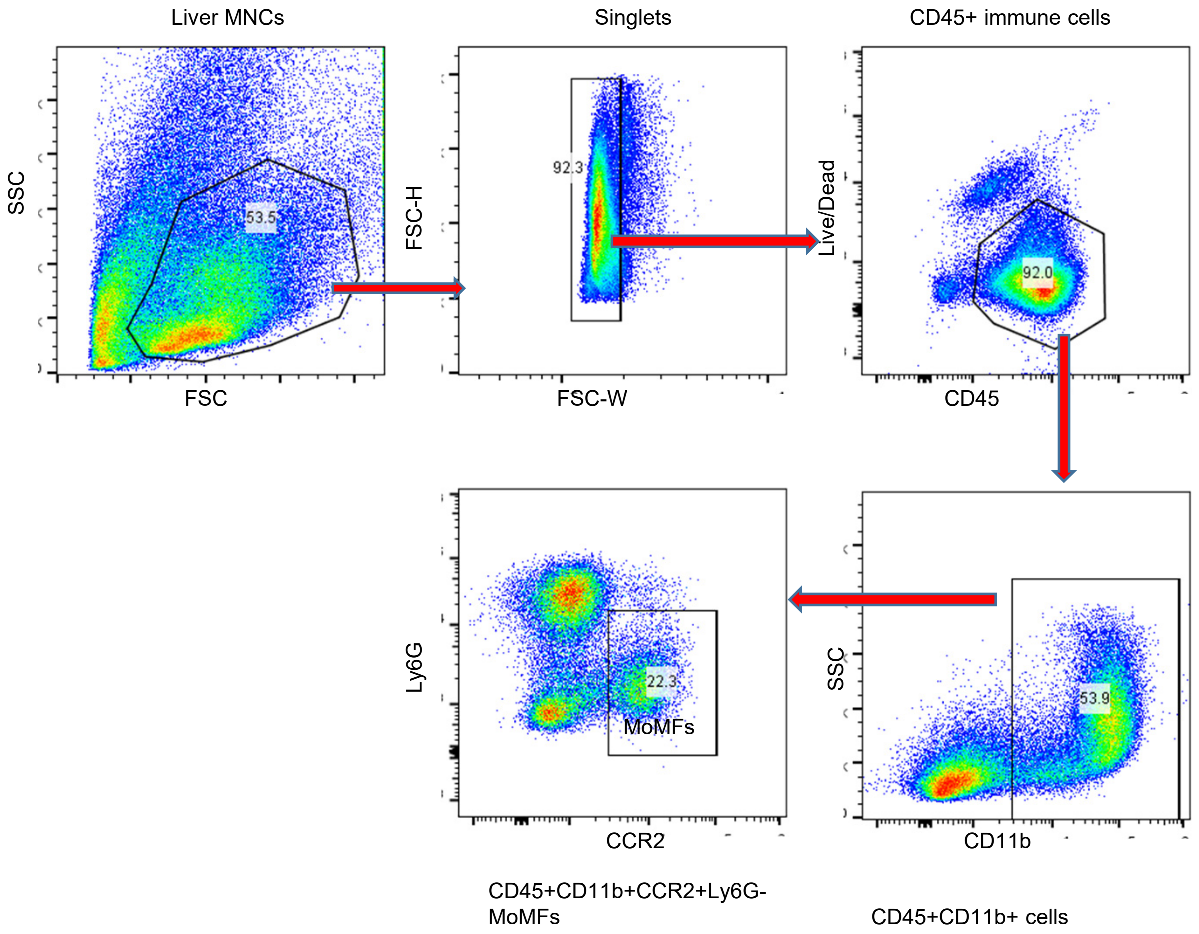

Supplemental Figure 15. **Liver MoMFs gating strategy. Liver MNCs were isolated from mice with or without ConA treatment.** These cells were stained with Zombie Yellow (live/dead dye) and surface markers: CD45, CD11b, Ly6G and CCR2. CD45+CD11b+CCR2+Ly6G^{-} cells were sorted as MoMFs.

Supplemental Figure 16A-E:

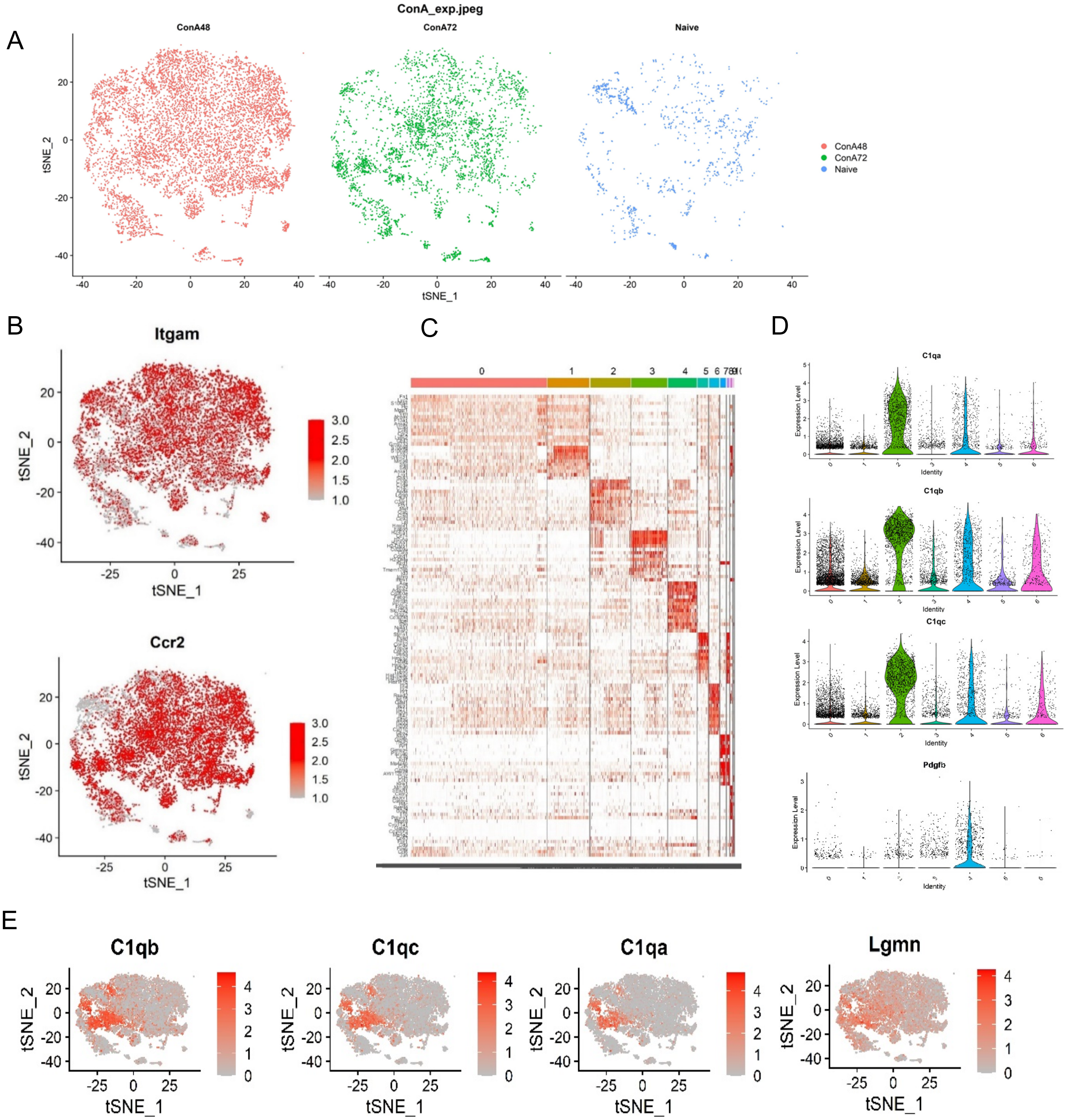

Supplemental Figure 16A-E. **Single cell RNA sequencing of MoMFs from livers after ConA treatment.** MoMFs were isolated from liver of C57BL/6 mice treated with ConA for 0, 48 and 72 hours. These cells were subjected to the 10X Genomics Chromium platform for single cell RNA sequencing. (A) t-SNE plots of cells from Naïve (1,106 cells), ConA 48 hrs (8,541 cells) and ConA 72 hrs (3,575 cells). (B) Feature plots for the gene expression of *CD11b* (*Itgam*) and *Ccr2* genes among all the cells. (C) Heatmap showing top 15 distinguishing genes per cluster. (D) Violin plots show the expression level of *C1q* genes and *Pdgfb* gene among the macrophage clusters. (E) Feature plots for the gene expression of *C1q* and *Lgmn* genes.

Supplemental Figure 16F:

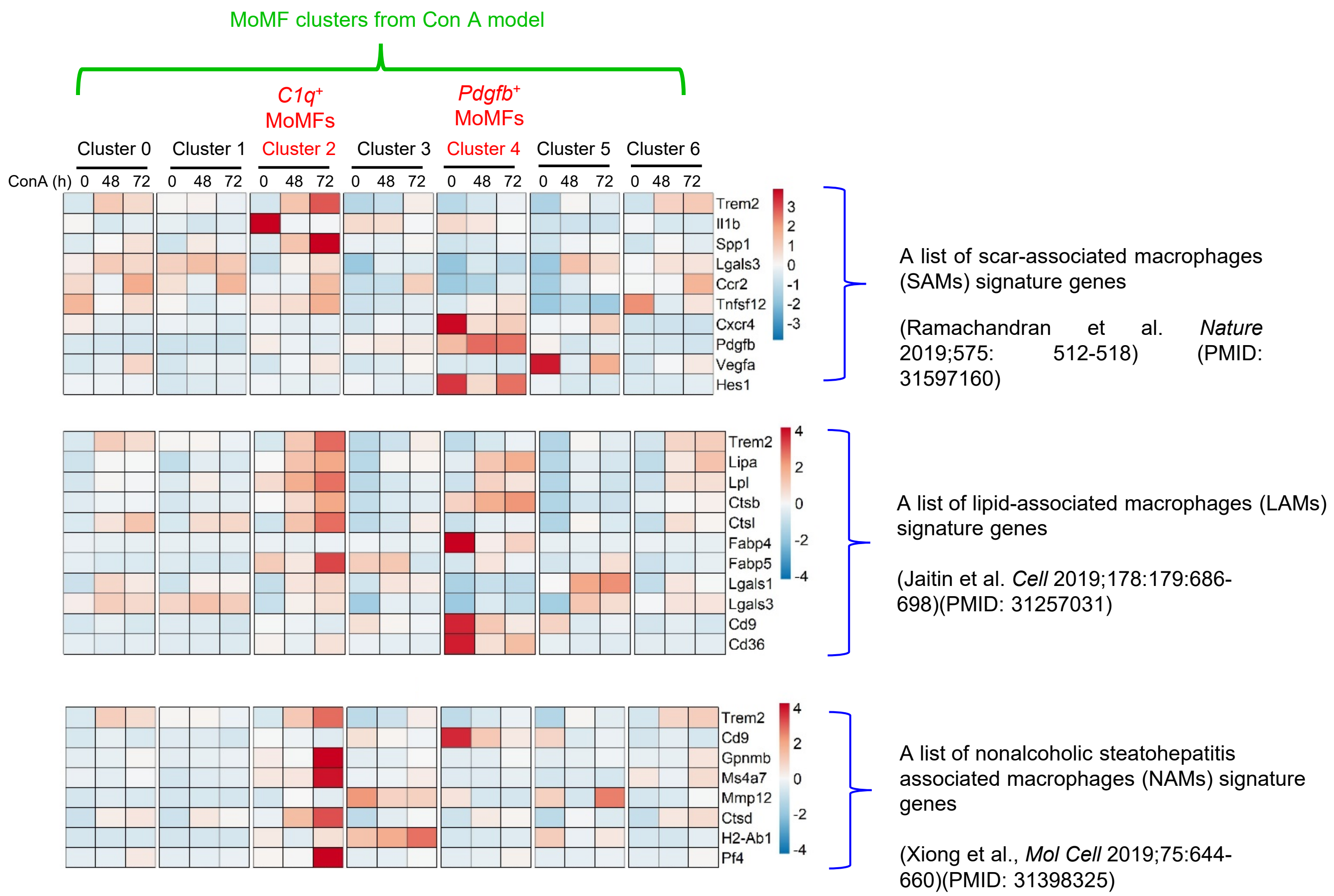

Supplemental Figure 16F. **Expression of SAM, NAM, LAM signature genes in MoMF clusters from livers after ConA treatment.** Heatmap of SAM, NAM, LAM signature genes in MoMF clusters from the livers of ConA-treated mice.

Supplemental Figure 17:

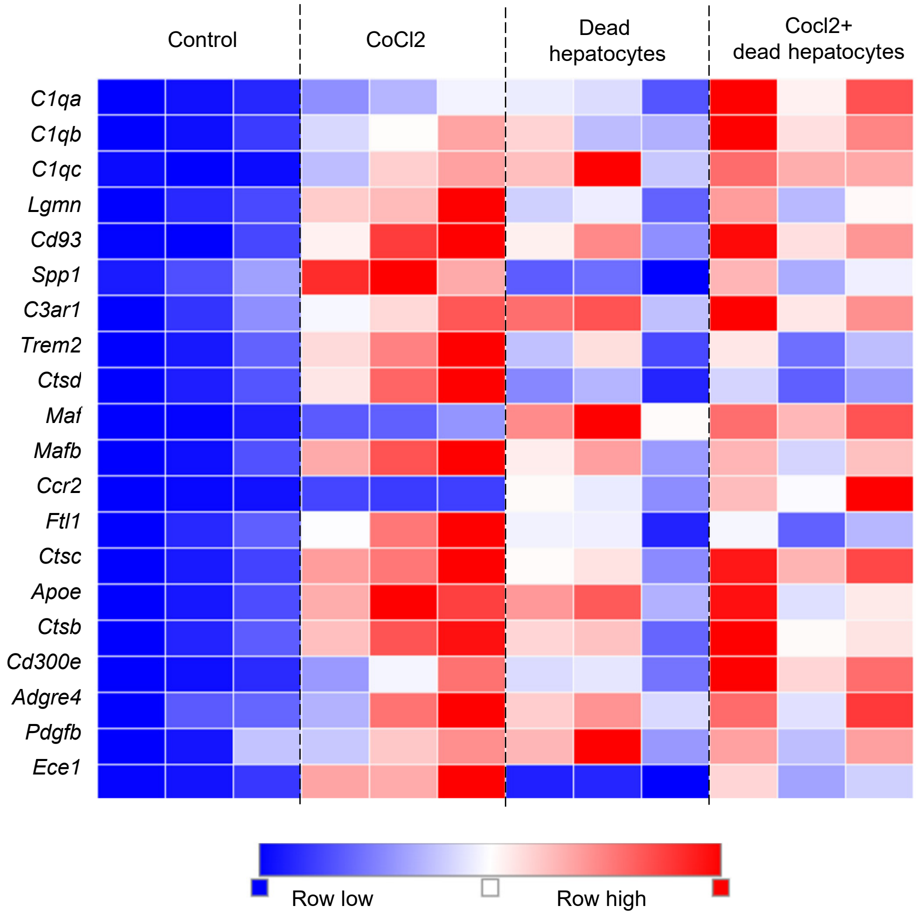

Supplemental Figure 17. **Hypoxia and dead hepatocytes trigger phenotype change in bone marrow derived macrophages (BMDMs) *in vitro*.** BMDMs from C57BL/6 mice with incubated with CoCl2 (100μM) and/or dead hepatocytes ($2X10^5$) for 24 hours, followed by RT-qPCR analysis of Cluster 2 and 4 signature genes. Heatmap of gene expression from 3 samples per group is shown.

Supplemental Figure 18

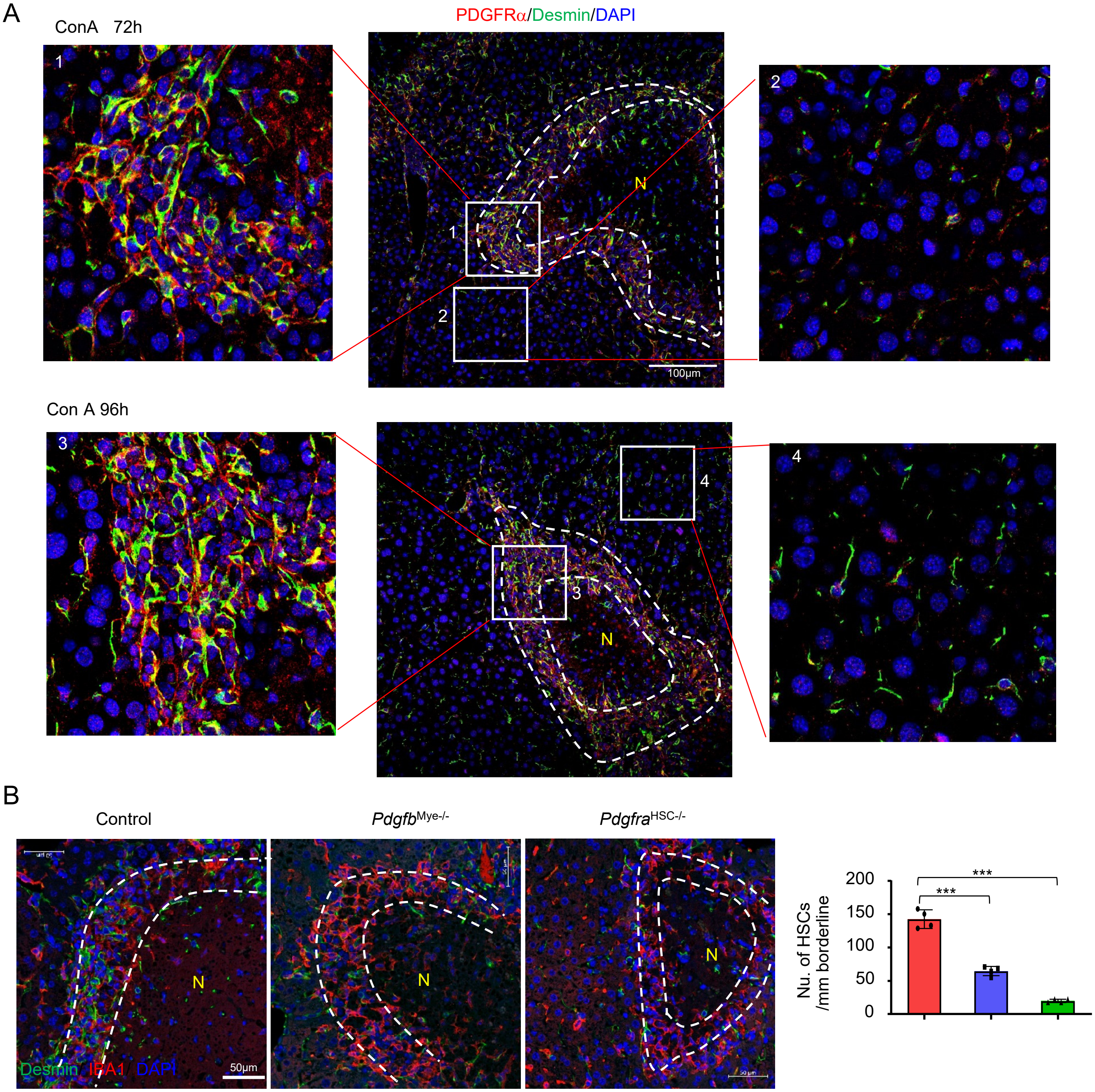

Supplemental Figure 18A. **Increased PDGFRα expression in HSCs in the border areas of necrosis in ConA-treated mice.** C57BL/6 mice were treated with ConA for 72 and 96 hours. PDGFRα and Desmin double staining on liver tissues. 1 and 3 indicated the border area of necrotic region, 2 and 4 indicated the non-injured area. Representative images from 4 mice in each group are shown.

Supplemental Figure 18B. **Reduced number of total HSCs in the border areas of necrosis in *Pdgfb*$^{mye-/-}$ and *Pdgfra*$^{HSC-/-}$ mice compared to WT mice post ConA injection.** WT, *Pdgfb*$^{mye-/-}$, *Pdgfra*$^{HSC-/-}$ mice were treated with ConA for 72 hours. Liver tissues were stained with Desmin and IBA1. Numbers of activated HSCs (aHSCs) in the border areas of necrosis were quantified and are shown on the right. Representative images from 4 mice in each group are shown. "N" indicates necrotic area. Values represent means ± SD, Statistical significance was assessed using 1-way ANOVA followed by Tukey's post hoc test for multiple groups (B). ***P<0.001.

Supplemental Figure 19

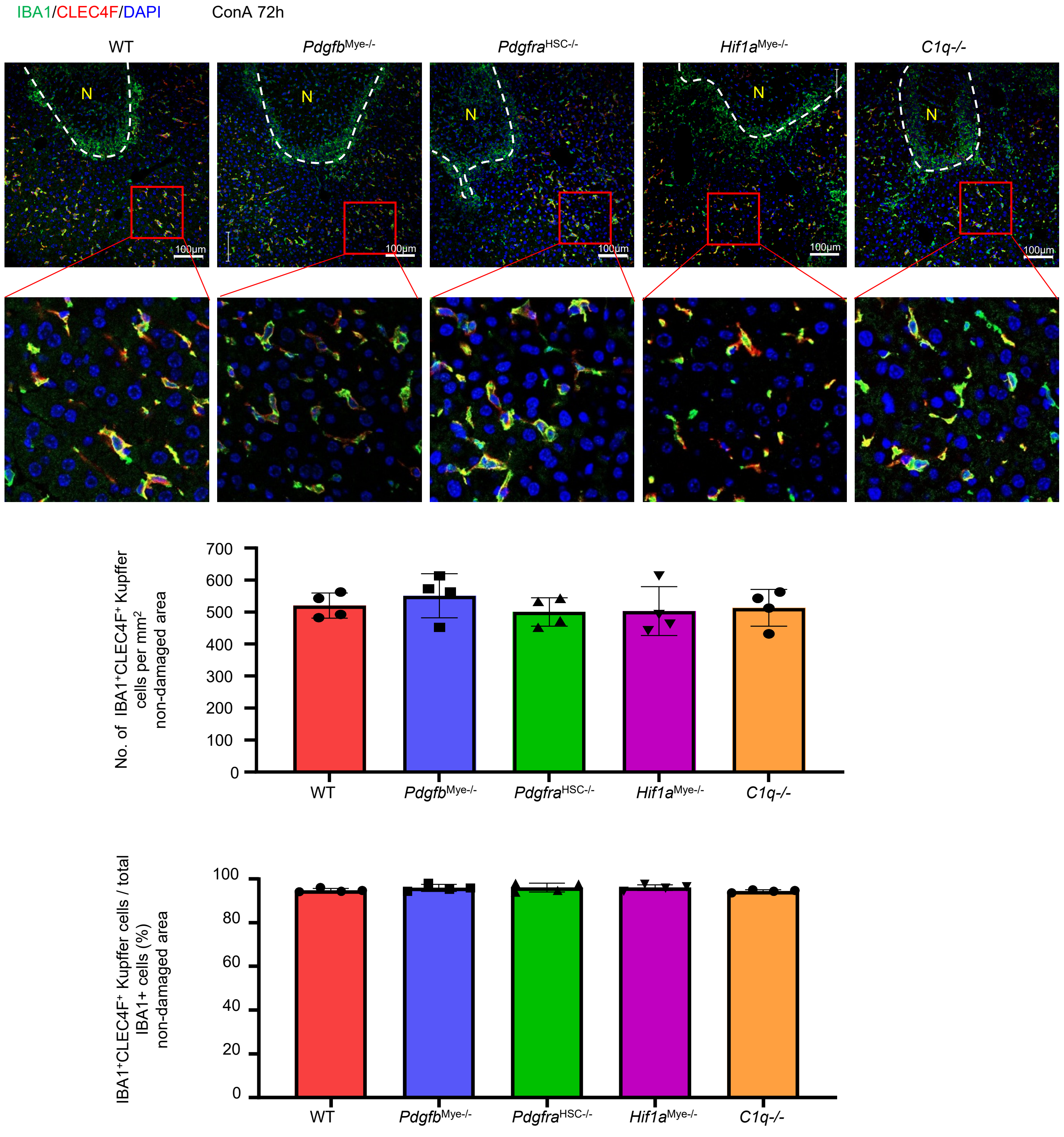

Supplemental Figure 19. **The number of IBA1+CLEC4F+ Kupffer cell number remain unchanged in non-damaged area after ConA induced liver injury in knockout mice used in this study compared to wild-type mice.** C57BL/6 (WT), *Pdgfb*$^{Mye-/-}$, *Pdgfra*$^{HSC-/-}$, *Hif1a*$^{Mye-/-}$ and *C1q*$^{-/-}$ mice were treated with ConA for 72 hours. Liver tissues were collected for double immunofluorescent staining of IBA1 and CLECF4. Enlarged images show non-damaged area (enlarged image). Quantitation of IBA and/or CLECF4 staining cells is shown in lower panel.

"N" in the images indicates necrotic area. Values represent means ± SD, n=4.

Supplemental Figure 20

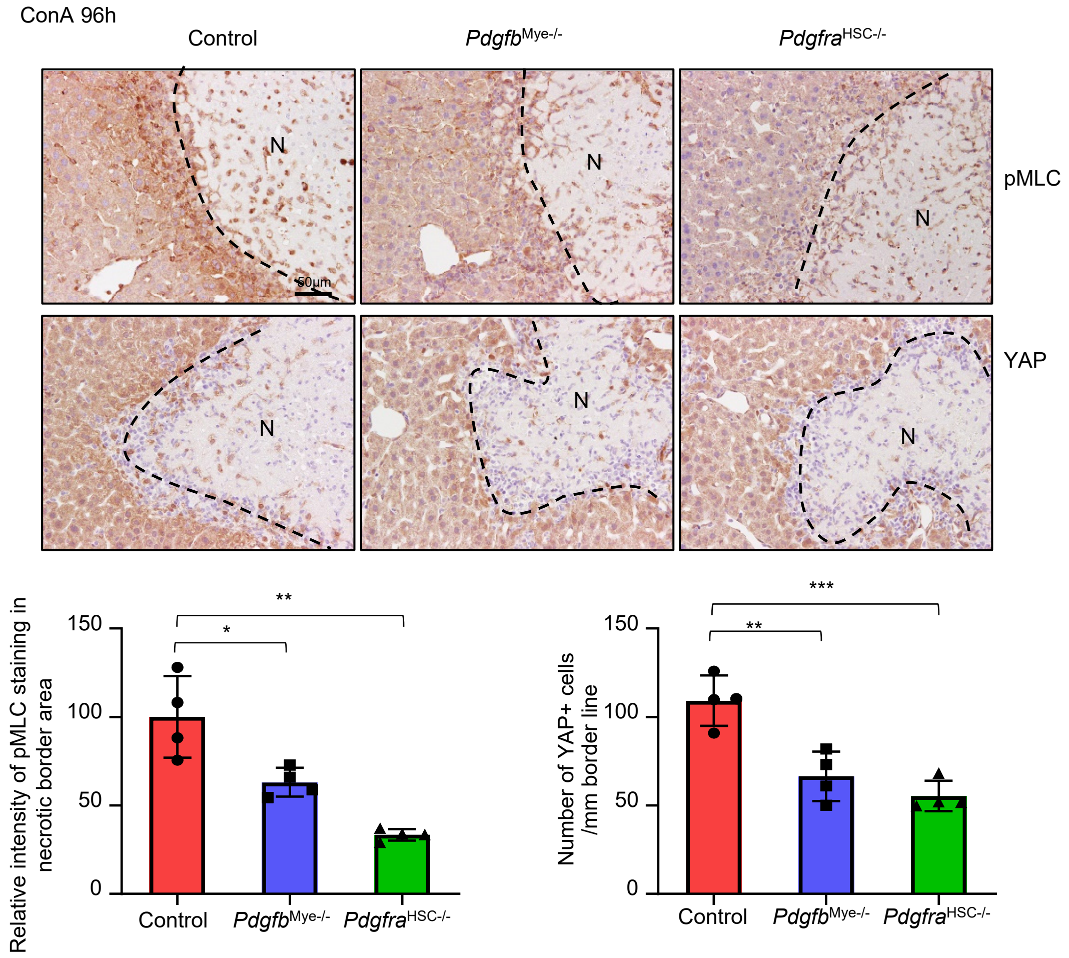

Supplemental Figure 20. **Reduced expression of the HSC contraction markers in the necrotic border areas from *Pdgfb*$^{mye-/-}$ *or Pdgfra*$^{HSC-/-}$ mice post ConA treatment.** WT, *Pdgfb*$^{mye-/-}$, *Pdgfra*$^{HSC-/-}$ mice were treated with ConA for 96 hours. pMLC and YAP staining of liver tissues. Dash line indicates the border area of necrotic area. “N” indicates necrotic area. Representative images from four mice in each group are shown. The pMLC intensity and number of YAP+ cells in necrotic border line were quantified as bar graph. Values represent means ± SD, n=4. Statistical significance was assessed using 1-way ANOVA followed by Tukey’s post hoc test for multiple groups (B). *P<0.05, **P<0.01, ***P<0.001.

Supplemental Figure 21

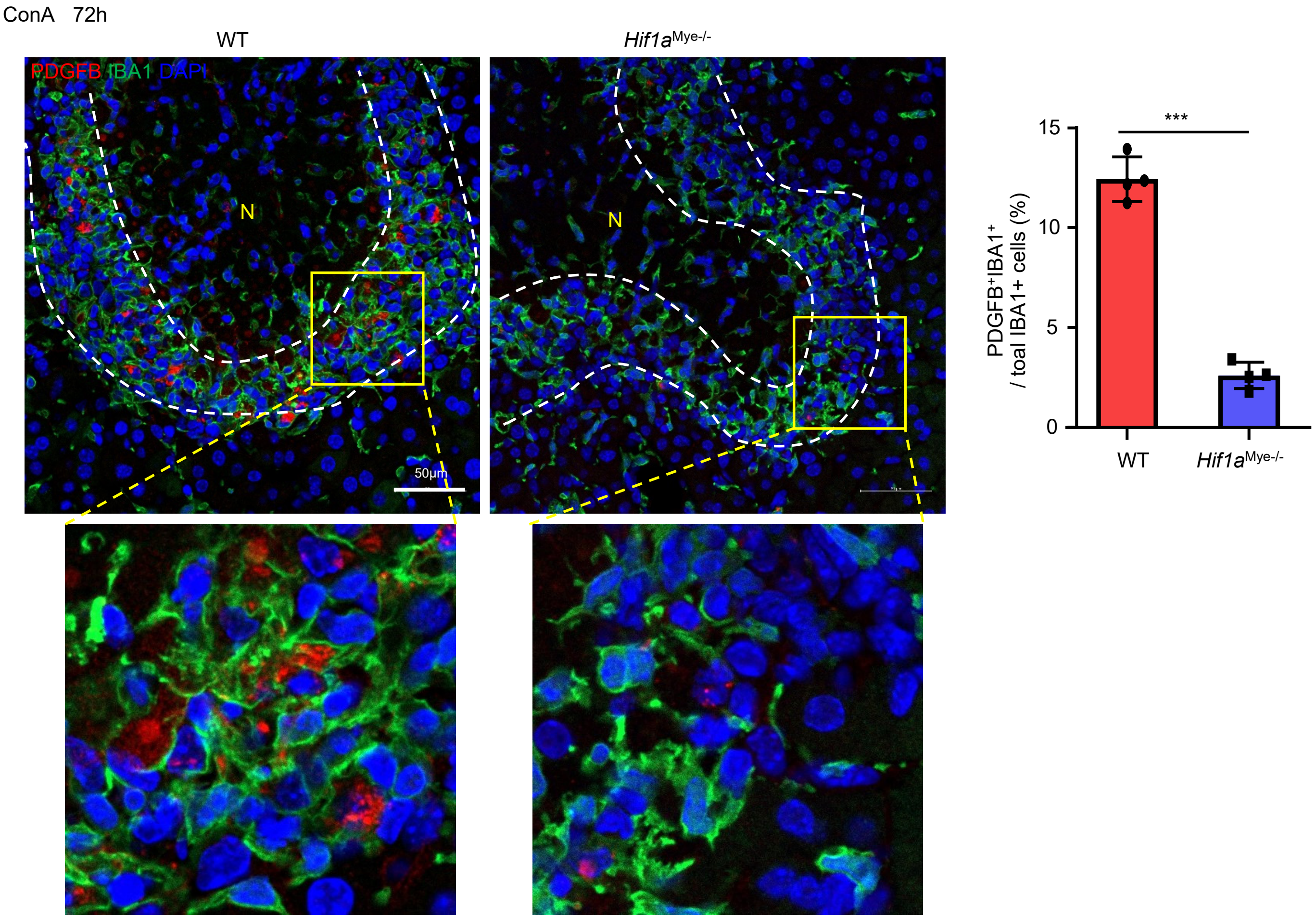

Supplemental Figure 21. **Reduced PDGFb expression in *Hif1a* deficient MoMFs from border area. *Hif1a*$^{Mye-/-}$ and WT mice were treated with ConA for 72 hours.** PDGFb and IBA1 double staining on liver tissues. Dash line indicates the border area of necrotic area. "N" indicates necrotic area. Representative images from four mice in each group are shown. The percentage of PDGFB+IBA1+ cells in all IBA1+ cells were quantified. Values represent means ± SD, n=4. Statistical significance was assessed using 2-tailed Student's t test for comparing 2 groups.****P*<0.001.

Supplemental Figure 22

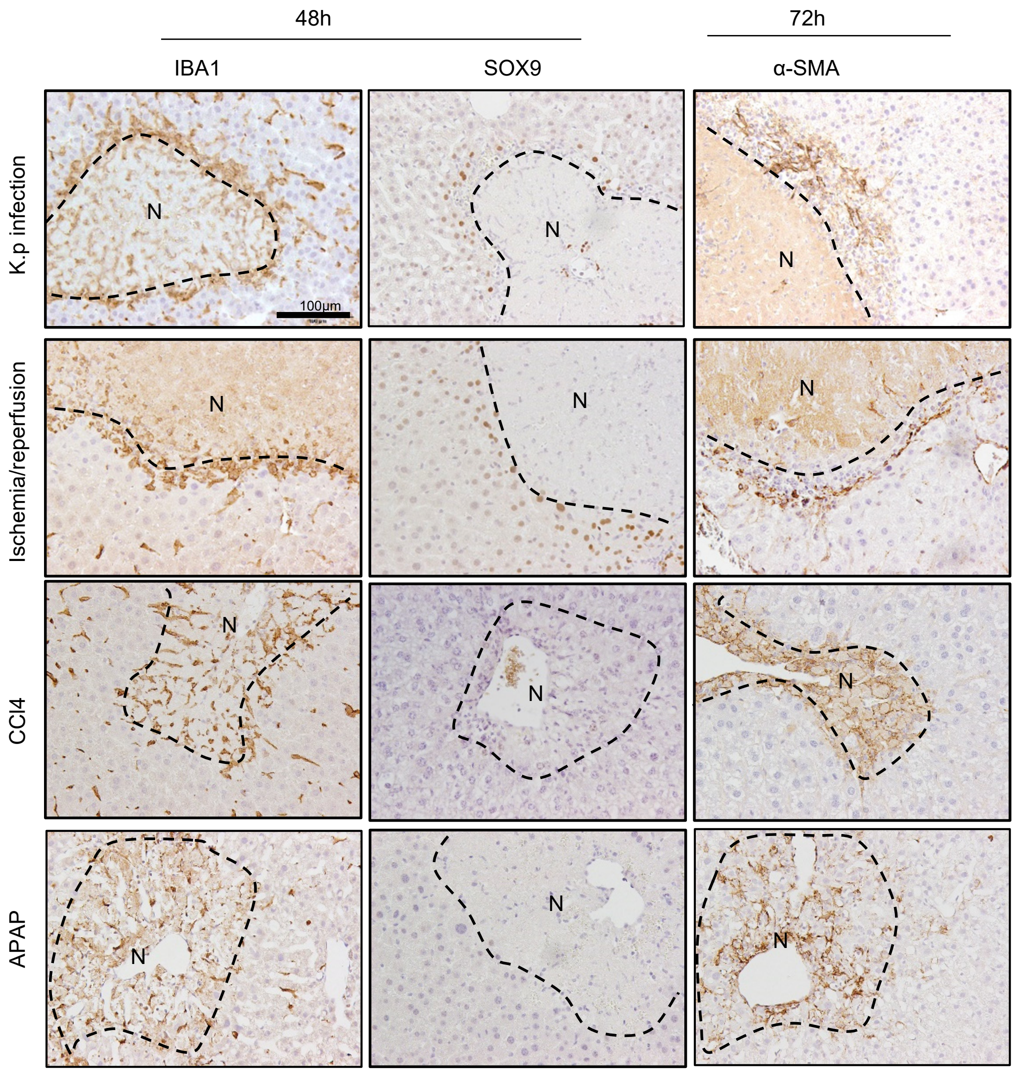

Supplemental Figure 22. **Macrophages, SOX9⁺ cells and activated HSCs in several liver injury models.** C57BL/6 mice were treated with *Klebsiella pneumoniae* (*K.p, 5000* CFU/mouse), CCl4 (0.2ml/kg), or APAP (250mg/kg), or were subjected to hepatic ischemia/reperfusion (I/R). Liver samples were collected 48 and 72 hours after treatment or surgery. IBA1, SOX9 and α-SMA staining were performed. Representative images from 3-4 in each group are shown.

"N" in the images indicates necrotic area.

Supplemental Figure 23

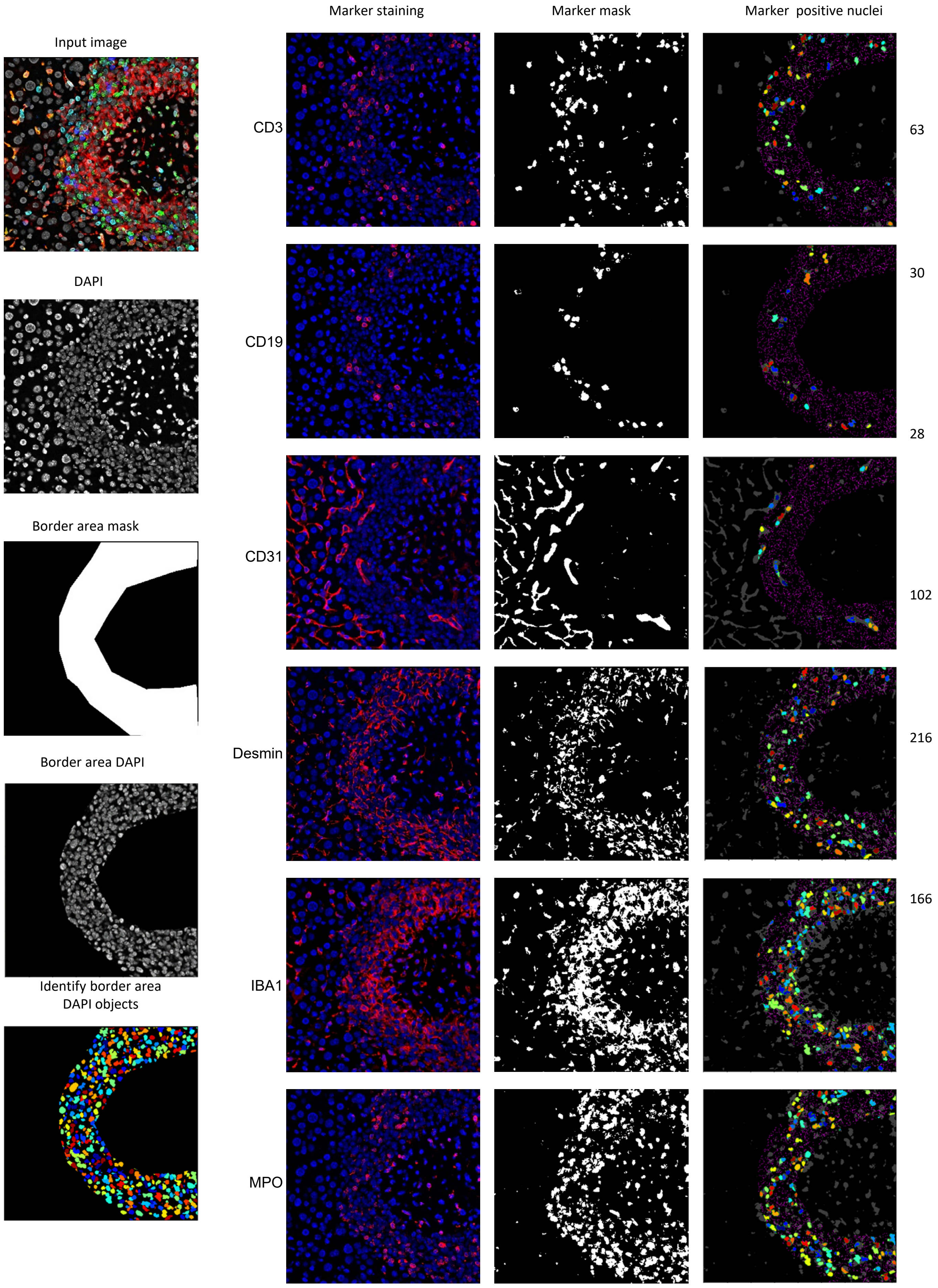

Supplemental Figure 23. Method for quantification of different cell types. Acquired images were analyzed by three free software tools including ImageJ, Ilastik and CellProfiler as described previously (Guillot,A et.al *Cancer* 12(9)2449). Briefly, border area masks were defined by morphology. T cells, B cells, liver sinusoidal endothelial cells (LSECs), hepatic stellate cells (HSCs), macrophages and neutrophils mask were generated by Ilastik, a trainable segmentation software, based on cell marker staining. Different cell numbers were identified and counted based on masked DAPI image using CellProfiler.

Table S1:

Antibody	Vendor	Cat.No	Dilution
Sox9	Sigma	HPA001758	1:200
IBA1	Wako	019-19741	1:1000
IBA1	Sigma	MABN92	1:500
pSTAT3	CST	9145	1:500
Jagged1	Abcam	ab109536	1:200
CD45	CST	70257	1:200
Hes1	CST	11988	1:200
Hif1α	CST	36169	1:200
EpCAM	Thermo Fisher Scientific	12-5791-82	1:200
CD3	CST	78588	1:200
CD19	Thermo Fisher Scientific	14-0194-82	1:200
Desmin	Abcam	ab15200	1:500
αSMA	CST	56856	1:200
MPO	Biocare Medical	PP023AA	prediluted
CD31	CST	77699	1:200
β-Catenin	BD Biosciences	610153	1:200
HNF4α	Abcam	ab41898	1:200
Bcl-xL	CST	2764	1:200
BrdU	BD Biosciences	551321	1:20
F4/80	CST	70076	1:200
CK19	Developmental Studies Hybridoma Bank	TROMA-III	1:200
p-MLC	CST	3675	1:200
PDGFRa	CST	3174	1:200
YAP	CST	14074	1:200
PDGFb	Sigma	SAB4502136	1:200

RFP	Rockland	600-401-379	1:200
Ki67	BD Biosciences	556003	1:200
Clec4F	R&D	MAB2784	1:200

Table S2:

**Target gene**	**Primer sequence**
Sox9	Forward Primer: AGTACCCGCATCTGCACAAC<br>Reverse Primer: ACGAAGGGTCTCTTCTCGCT
Hes1	Forward Primer: TCAACACGACACCGGACAAAC<br>Reverse Primer: ATGCCGGGAGCTATCTTTCTT
Hes5	Forward Primer: AGTCCCAAGGAGAAAAACCGA<br>Reverse Primer: GCTGTGTTTCAGGTAGCTGAC
Jag1	Forward Primer: ATGCAGAACGTGAATGGAGAG<br>Reverse Primer: GCGGGACTGATACTCCTTGAG
IGF1	Forward Primer: CACATCATGTCGTCTTCACACC<br>Reverse Primer:GGAAGCAACACTCATCCACAATG
BMP2	Forward Primer : GGGACCCGCTGTCTTCTAGT<br>Reverse Primer: TCAACTCAAATTCGCTGAGGAC
Ihh	Forward Primer : CTCTTGCCTACAAGCAGTTCA<br>Reverse Primer: CCGTGTTCTCCTCGTCCTT
Dhh	Forward Primer : CTTGGCACTCTTGGCACTATC<br>Reverse Primer: CAGAGGCACAAGTTGCTTGC
Fgf10	Forward Primer : TCAGCGGGACCAAGAATGAAG<br>Reverse Primer: CGGCAACAACTCCGATTTCC
Shh	Forward Primer: AAAGCTGACCCCTTTAGCCTA<br>Reverse Primer: TGAGTTCCTTAAATCGTTCGGAG
Nos2	Forward Primer: GTTCTCAGCCCAACAATACAAGA<br>Reverse Primer: GTGGACGGGTCGATGTCAC
Cd86	Forward Primer: TCAATGGGACTGCATATCTGCC<br>Reverse Primer: GCCAAAATACTACCAGCTCACT
Chil3	Forward Primer: CAGGTCTGGCAATTCTTCTGAA<br>Reverse Primer: GTCTTGCTCATGTGTGTAAGTGA
Arg1	Forward Primer: CTCCAAGCCAAAGTCCTTAGAG<br>Reverse Primer: GGAGCTGTCATTAGGGACATCA
Fcrla	Forward Primer: GATGATGGCGATATGACCCAAT<br>Reverse Primer: GCAGAACCAATGTGTCTCCTTC

Gpnmb	Forward Primer: TGCCAAGCGATTTCGTGATGT<br>Reverse Primer: GCCACGTAATTGGTTGTGCTC
Marco	Forward Primer: CCTCCAGGGACTTACGGGT<br>Reverse Primer: CCAGTGAGACCTATGTCACCT
Cd81	Forward Primer: GCTCTTCGTCTTCAATTTCGTCT<br>Reverse Primer: TGTTGGGTGCCGGTTTGTT
Cd5l	Forward Primer: GGGGTTGACTGCAACGGAA<br>Reverse Primer: GGCCATCTACTAGACGCACA
Stab2	Forward Primer: GTTGCTTGTGCAAAATGCCTG<br>Reverse Primer: GCACTCCGTCTTGATGGTTAGAG
Havcr2	Forward Primer: TCAGGTCTTACCCTCAACTGTG<br>Reverse Primer: GGCATTCTTACCAACCTCAAACA
C1qa	Forward Primer: TTCGGCAGAACCCAATGACG<br>Reverse Primer: TGGTATGGACTCTCCTGGTTG
C1qb	Forward Primer: CGTCGGCCCTAAGGGTACT<br>Reverse Primer: GGGGCTGTTGATGGTCCTC
C1qc	Forward Primer: GGACGGGCATGATGGACTC<br>Reverse Primer: TTCTGTTTGTATCGGCCCTCC
Lgmn	Forward Primer: TGGACGATCCCGAGGATGG<br>Reverse Primer: CGGTGGATGATCTGGTAGGC
Cd93	Forward Primer: GCCATCTCAACTGGTTTGTTCC<br>Reverse Primer: ACTCTTCACGGTGGCAAGATT
Spp1	Forward Primer: ATCTCACCATTCGGATGAGTCT<br>Reverse Primer: TGTAGGGACGATTGGAGTGAAA
C3ar1	Forward Primer: TCGATGCTGACACCAATTCAA<br>Reverse Primer: AGTCCCAATAGACAAGTGAGACC
Trem2	Forward Primer: CTGGAACCGTCACCATCACTC<br>Reverse Primer: CGAAACTCGATGACTCCTCGG
Ctsd	Forward Primer: GCTTCCGGTCTTTGACAACCT<br>Reverse Primer: CACCAAGCATTAGTTCTCCTCC
Ctsb	Forward Primer: CAGGCTGGACGCAACTTCTAC<br>Reverse Primer: TCACCGAACGCAACCCTTC
Maf	Forward Primer: GGAGACCGACCGCATCATC<br>Reverse Primer: TCATCCAGTAGTAGTCTTCCAGG
Mafb	Forward Primer: TTCGACCTTCTCAAGTTCGACG<br>Reverse Primer: GAGATGGGTCTTCGGTTCAGT
Ftl1	Forward Primer: CGTCAGAATTATTCCACCGAGG<br>Reverse Primer: GCCACGTCATCCCGATCAAA
Ctsc	Forward Primer: GTTCCCGAAGCGACATTAACT<br>Reverse Primer: TCGTAGGCAGTATCCAACTTCTT
Apoe	Forward Primer: CTCCCAAGTCACACAAGAACTG<br>Reverse Primer: CCAGCTCCTTTTTGTAAGCCTTT
Cd300e	Forward Primer: TGGGTCTTACTGGTGCAAGAT<br>Reverse Primer: CTTACACTGACCGATGGATCAC
Adgre4	Forward Primer: TGCAATAGCTGGCCACAAGA<br>Reverse Primer: CAAGATAATGGCTGCCGCTG

Ccnd1	Forward Primer: GCGTACCCTGACACCAATCTC<br>Reverse Primer: ACTTGAAGTAAGATACGGAGGGC
Wfdc21	Forward Primer: TGTGGGCCAGAGGAACAATG<br>Reverse Primer: ACTCCACTGTGCTGCTTGTA
Lcn2	Forward Primer: TGGCCCTGAGTGTCATGTG<br>Reverse Primer: CTCTTGTAGCTCATAGATGGTGC
S100a8	Forward Primer: ACAATGCCGTCTGAACTGGA<br>Reverse Primer: CATCGCAAGGAACTCCTCGAA
Pdgfb	Forward Primer: CATCCGCTCCTTTGATGATCTT<br>Reverse Primer: GTGCTCGGGTCATGTTCAAGT
Il6	Forward Primer: TAGTCCTTCCTACCCCAATTTCC<br>Reverse Primer: TTGGTCCTTAGCCACTCCTTC
18S	Forward Primer: AACTTTCGATGGTAGTCGCCGT<br>Reverse Primer: TCCTTGGATGTGGTAGCCGTTT
